# Experimental data showing the thermal behavior of a flat roof with phase change material

**DOI:** 10.1016/j.dib.2015.09.019

**Published:** 2015-10-22

**Authors:** Ayça Tokuç, Tahsin Başaran, S. Cengiz Yesügey

**Affiliations:** a Department of Architecture, Dokuz Eylül University, DEÜ Tınaztepe Campus, 35160 Buca, İzmir, Turkey; b Department of Architecture, İzmir Institute of Technology, İYTE Gülbahçe Campus, 35430 Urla, İzmir, Turkey

**Keywords:** Thermal energy storage, Latent energy storage, Phase change material, Roof, Thermal behavior

## Abstract

The selection and configuration of building materials for optimal energy efficiency in a building require some assumptions and models for the thermal behavior of the utilized materials. Although the models for many materials can be considered acceptable for simulation and calculation purposes, the work for modeling the real time behavior of phase change materials is still under development. The data given in this article shows the thermal behavior of a flat roof element with a phase change material (PCM) layer. The temperature and energy given to and taken from the building element are reported. In addition the solid–liquid behavior of the PCM is tracked through images. The resulting thermal behavior of the phase change material is discussed and simulated in [1] A. Tokuç, T. Başaran, S.C. Yesügey, An experimental and numerical investigation on the use of phase change materials in building elements: the case of a flat roof in Istanbul, Build. Energy, vol. 102, 2015, pp. 91–104.

**Specifications table**

**Table:** 

Subject area	*Physics*
More specific subject area	*Thermal Energy Storage*
Type of data	*.xls data, .m matlab script, .jpg images*
How data was acquired	*Temperature and flow measurements were taken with thermocouples (T-type), thermal resistance thermometer (PT-*100*), electromagnetic flowmeter (LD-*10*) and datalogger (Agilent* 34972*). The images were taken with a low resolution web camera*
Data format	*Processed experimental data, raw images,.m file is for calibration*
Experimental factors	*The layers of the building element were set at* 25 °C *temperature before starting the experiment*
Experimental features	*Thermal energy is given to and taken from the flat roof element with a phase change material layer*
Data source location	*Turkey*
Data accessibility	*Data is available within this article*

**Value of the data**

• *The data contain thermal measurements from a laboratory experiment of a flat roof element with a phase change material layer.*
• *The data provide a reference and may serve to benchmark thermal models for the used phase change material by other groups.*
• *In addition, the phase change material was observed in minute intervals and these images can be used to further the understanding of the the real time thermal behavior of a paraffin based phase change material* and the effect of natural convection during phase change.

## 1 Data, experimental design, materials and methods

Three types of data are presented and given in Supplementary files of this data article. First contain the raw measurements from the experimental design. This data represents the amount of energy given and taken the experimental system and the thermal changes inside the layers of a flat roof with a phase change material (PCM) layer. Second, the files used to calibrate the measurements are given. They include the calculation script for the raw data and the coefficients for the calibration curves. The third data shows the minute changes in the PCM layer with photographs taken during the experiment.

### 1.1 Experimental Procedure

The Supplementary data shows the thermal behavior inside a flat roof with phase change material (PCM) with a stable indoor surface temperature of 25 °C and parametric outdoor surface conditions. The experimental work was performed on a typical flat roof that corresponds to the Turkish thermal insulation code, TS-825 [2]. The roof element has a surface area of 50 cm by 50 cm and is composed of; 10 cm reinforced concrete, 6 cm extruded polystyrene thermal insulation, 5 cm phase change material, 1 cm water insulation layer, 5 cm leveling concrete, 0.8 cm binding mortar and 0.7 cm ceramic layers.

During the experiment, the roof element was put into a box with 15 cm thermal insulation on all sides. The surface temperatures were stabilized by copper serpentines, through which conditioned water runs. Therefore they give energy to and take energy from the upper and lower surfaces of the roof that represent the outdoor surface and indoor surface respectively. The thermal changes within the roof element are measured and the energy given to and taken from a surface, and the energy stored inside the roof are calculated to ensure the validity of the experiment. The experimental method is discussed in more detail in [1]. The outdoor surface target temperature is 50 °C for the data given in this article.

### 1.2 The Measurements

**Fig. 1:**
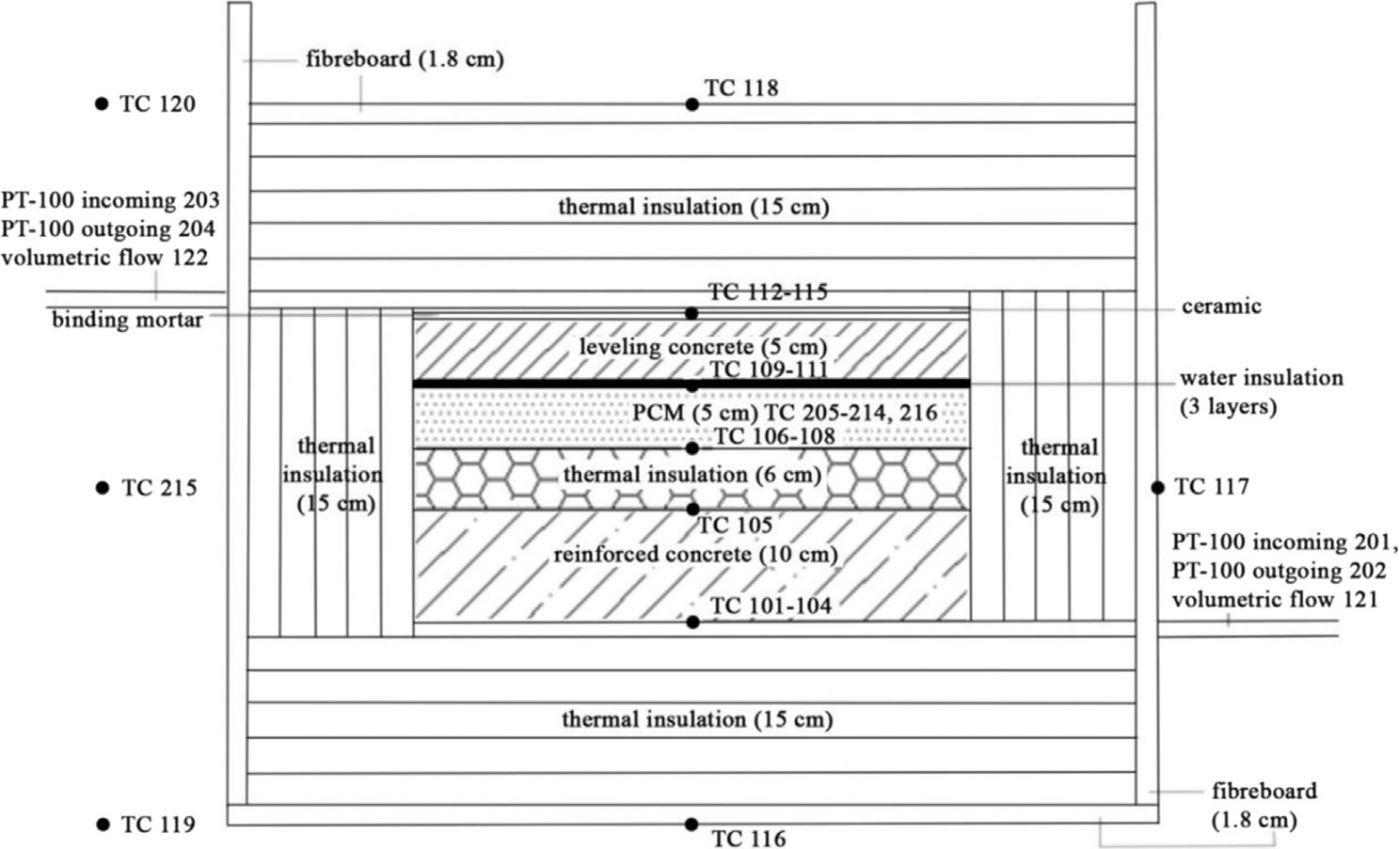
Data acquisition points in the .xls.

All of the data acquisition points are shown in Fig. 1 and the data taken from the acquisition points are given in Supplementary Table 1. For sensitivity analysis of the experiment see Table 2 in [1]. The methodology of the sensitivity analysis is given in [3].

The thermocouples and PT-100 were calibrated at EGEKALMEM laboratory. The 95% sensitivity of the PT-100 measurement during calibration is calculated by Eq. (1).

(1) $$\text{Sensitivity}\;\text{with}\;\text{95\%}\;\text{confidence}=2\sqrt{{\left(\frac{{\mathrm{Ref}}_{\mathrm{Pt}}}{2}\right)}^{2}+{\left(\frac{\frac{{\text{T}}_{\text{d}}}{2}}{\sqrt{3}}\right)}^{2}+{\left(\frac{{\mathrm{Ref}}_{\mathrm{mm}}}{2}\right)}^{2}+{\mathrm{St}}^{2}+{{\text{B}}_{\text{s}}}^{2}}$$

*T*_*d*_ shows the resolution of the datalogger and is taken as 0.001 [4]. *Ref*_*mm*_ is the reference value of the multimeter and is 0.0024 [5]. The standard deviation (*St*) is 0.01 at the maximum. The thermal stability of the constant temperature baths (*B*_*s*_) is 0.01 [5]. The reference value of PT-100 (*Ref*_*Pt*_) is taken as 0.01 at 0 °C and 0.02 at 100 °C, therefore the sensitivity of PT-100 measurements is ±0.035 with 95% confidence.

The calibration curves of the thermocouples are calculated with 95% confidence with matlab software. The calculations can be found in [6]. The raw data was filtered according to the calibration functions via the Supplementary Script 1. The calibration function, *f*(*x*) is as given in Eq. (2).

(2) $$f\left(x\right)=ax^{3}+bx^{2}+cx+d$$

**Fig. 2:**
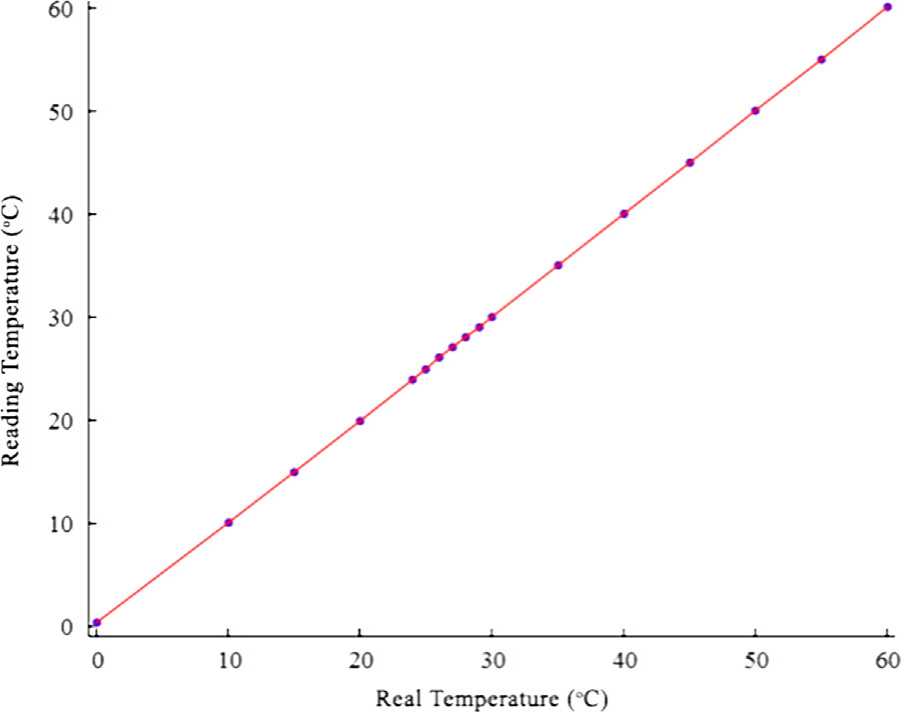
Calibration curve of TC210.

For example, TC210 measures the temperature in the middle of the PCM layer. The calibration curve for TC210 is given in Fig. 2 and found as in Eq. (3) with 95% confidence level.

(3) $$f\left(x\right)=9.23E-06x^{3}–0.00104x^{2}+1.03x–0.329$$

The a, b, c, and d coefficients derived for calibration curves are given in Supplementary Table 2

### 1.3 Phase change

**Fig. 3:**
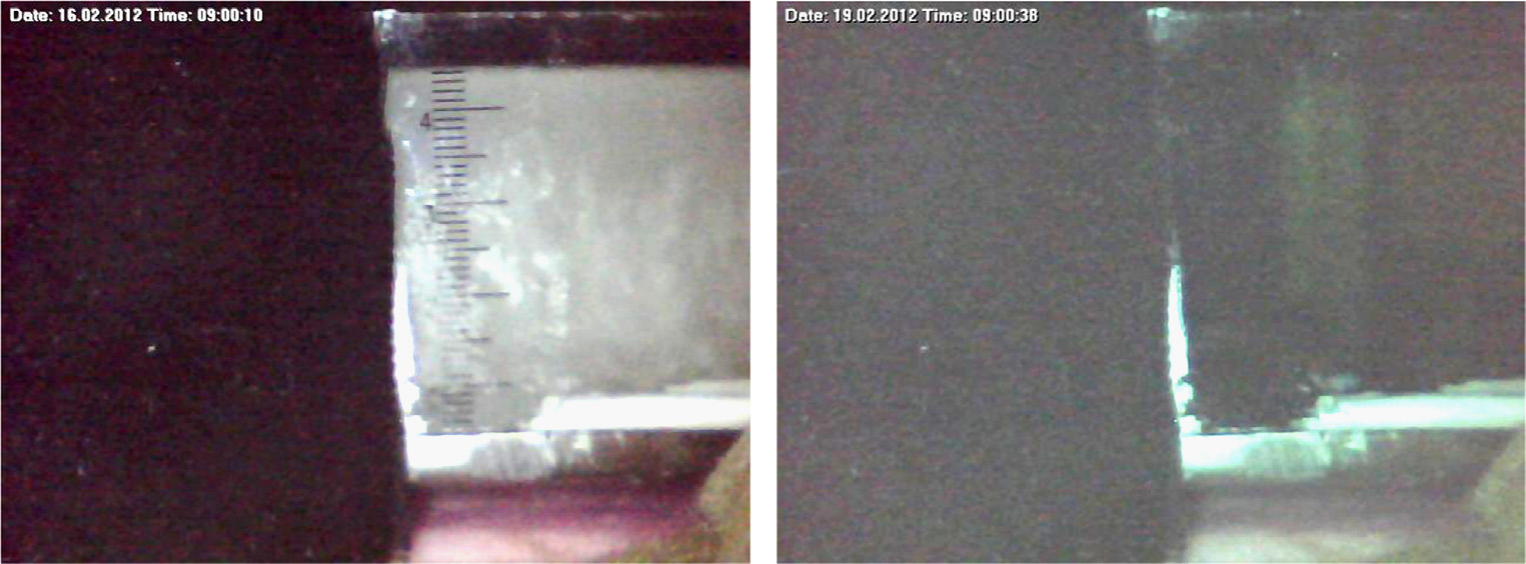
*Photos showing* the PCM at the start (left) and the end (right) of the experiment.

In addition to measurement of the temperature changes inside the PCM, the behavior of the PCM was also observed by taking photographs of the PCM layer during the experiment. The left side of Fig. 3 shows the solid state of the PCM at the start of the experiment, while the right side of the Fig. 3 shows the liquid state at the end of the experiment, at 72 h. These photos are taken at 1 min intervals and are also given in the supplementary material (Supplementary Fig. 1–4319).

## Figures and Tables

**Figure:**
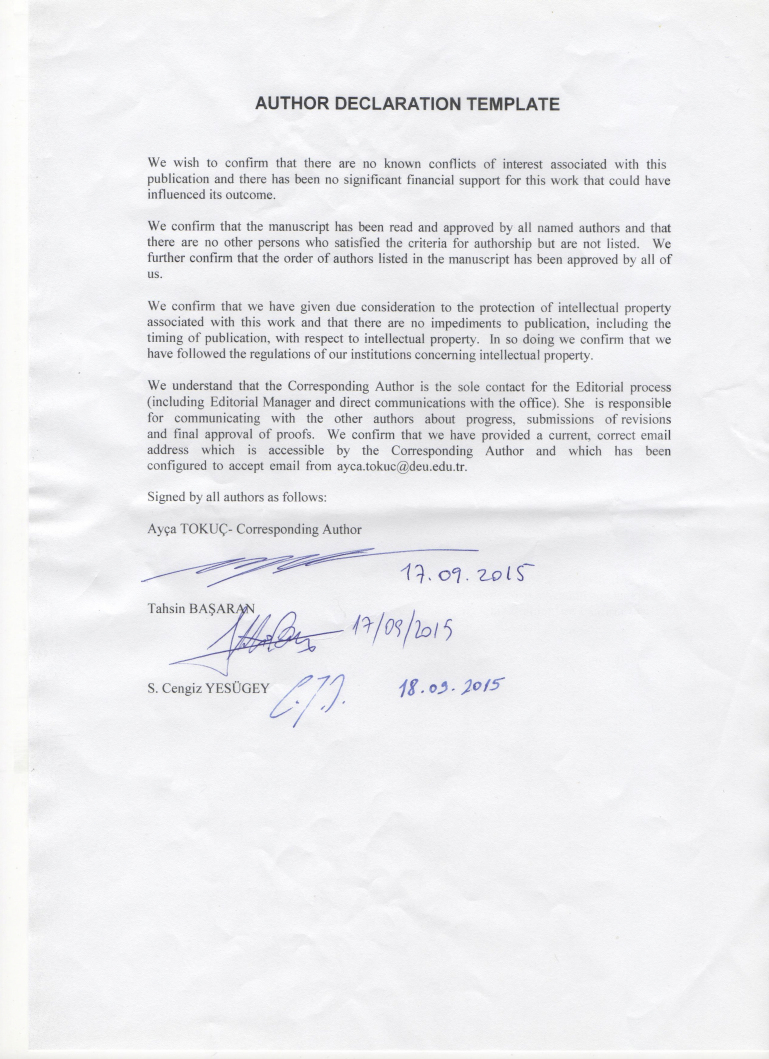


## Footnotes

Appendix A Supplementary data associated with this article can be found in the online version at doi:10.1016/j.dib.2015.09.019.
