# Supplementary figures and images for: Experimental data showing the thermal behavior of a flat roof with phase change material (part 2 of 2)
Source: Data Brief. 2015 Oct 22;5:476–80. doi: 10.1016/j.dib.2015.09.019 (PMC4631864; doi:10.1016/j.dib.2015.09.019)

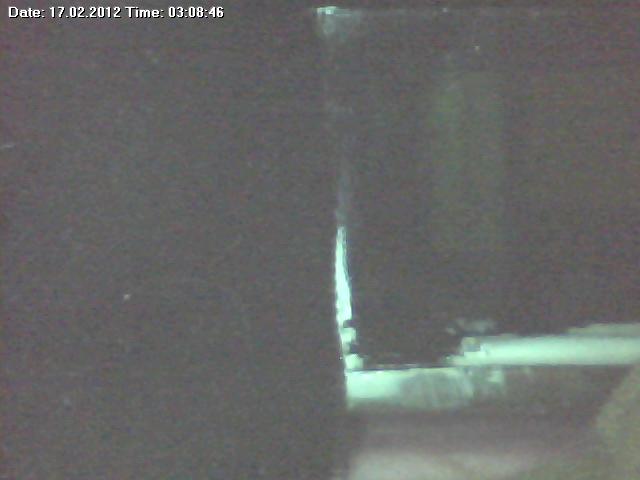

Supplement: Supplementary file 1 — Supplementary material [file mmc1.zip › Supplementary files/Supplementary Figure 1089.jpg]

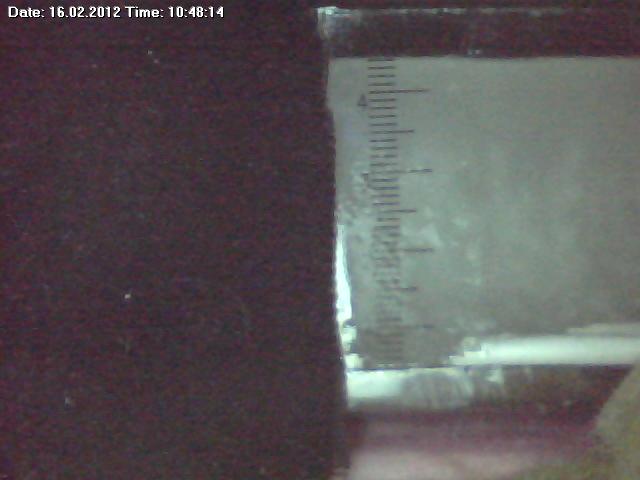

Supplement: Supplementary file 1 — Supplementary material [file mmc1.zip › Supplementary files/Supplementary Figure 109.jpg]

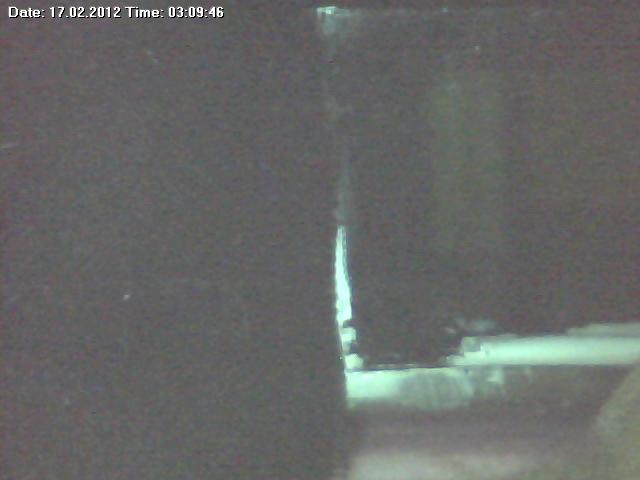

Supplement: Supplementary file 1 — Supplementary material [file mmc1.zip › Supplementary files/Supplementary Figure 1090.jpg]

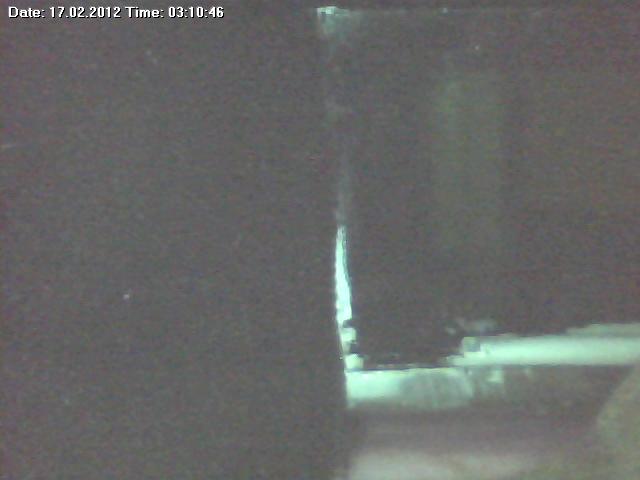

Supplement: Supplementary file 1 — Supplementary material [file mmc1.zip › Supplementary files/Supplementary Figure 1091.jpg]

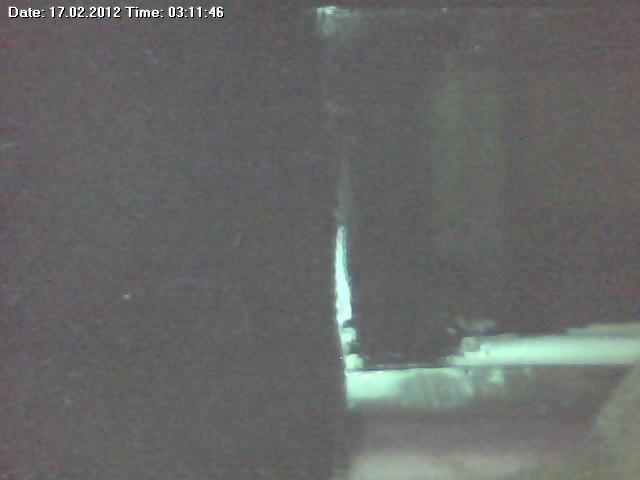

Supplement: Supplementary file 1 — Supplementary material [file mmc1.zip › Supplementary files/Supplementary Figure 1092.jpg]

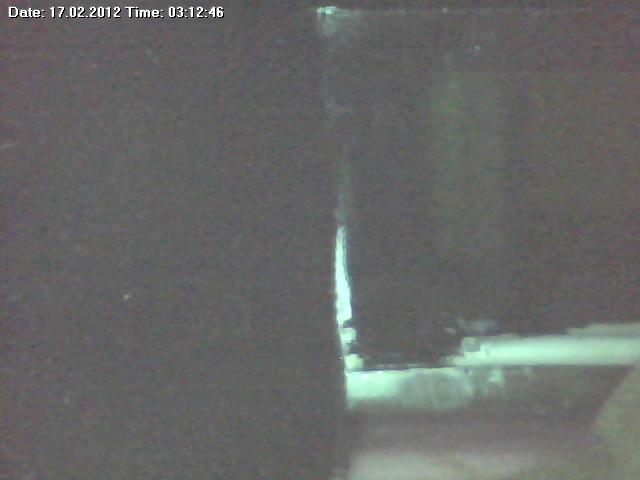

Supplement: Supplementary file 1 — Supplementary material [file mmc1.zip › Supplementary files/Supplementary Figure 1093.jpg]

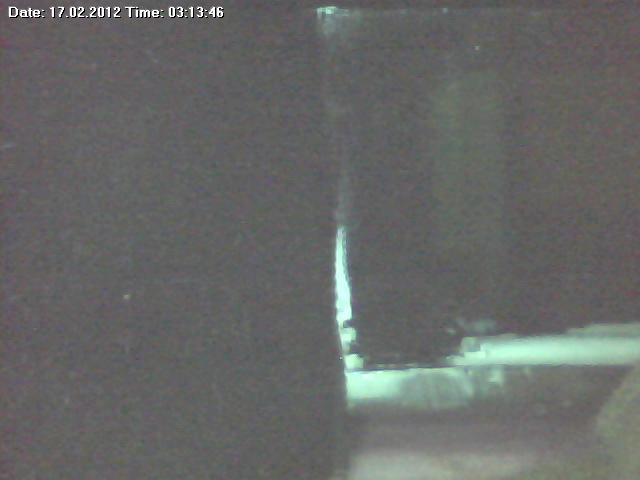

Supplement: Supplementary file 1 — Supplementary material [file mmc1.zip › Supplementary files/Supplementary Figure 1094.jpg]

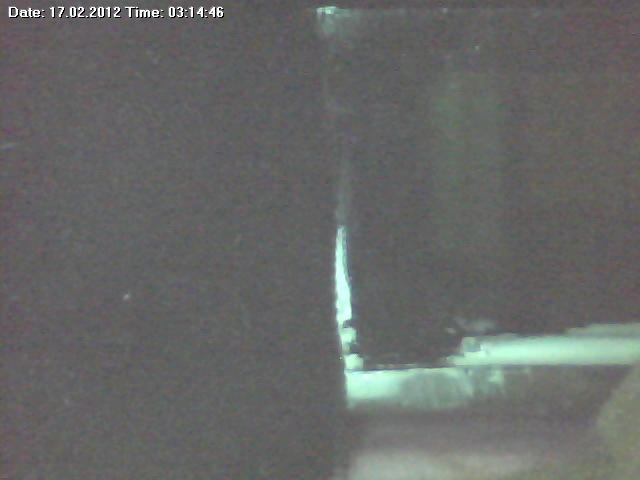

Supplement: Supplementary file 1 — Supplementary material [file mmc1.zip › Supplementary files/Supplementary Figure 1095.jpg]

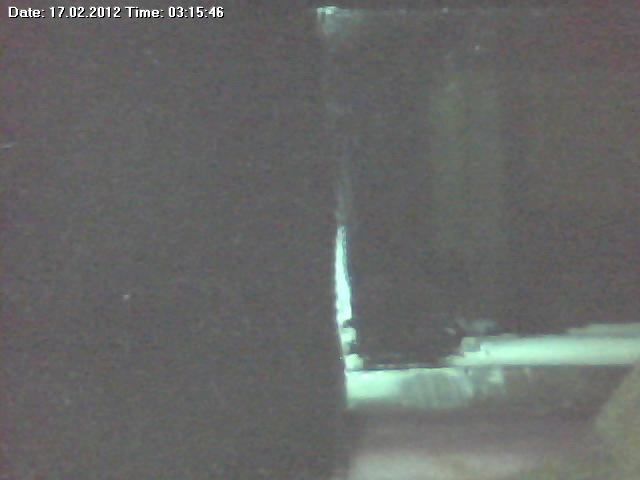

Supplement: Supplementary file 1 — Supplementary material [file mmc1.zip › Supplementary files/Supplementary Figure 1096.jpg]

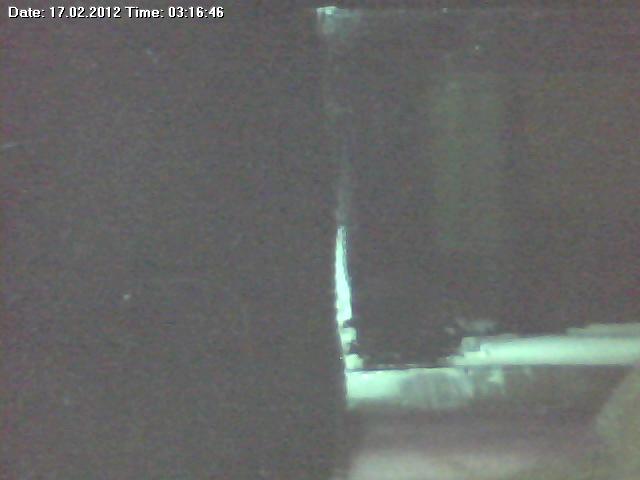

Supplement: Supplementary file 1 — Supplementary material [file mmc1.zip › Supplementary files/Supplementary Figure 1097.jpg]

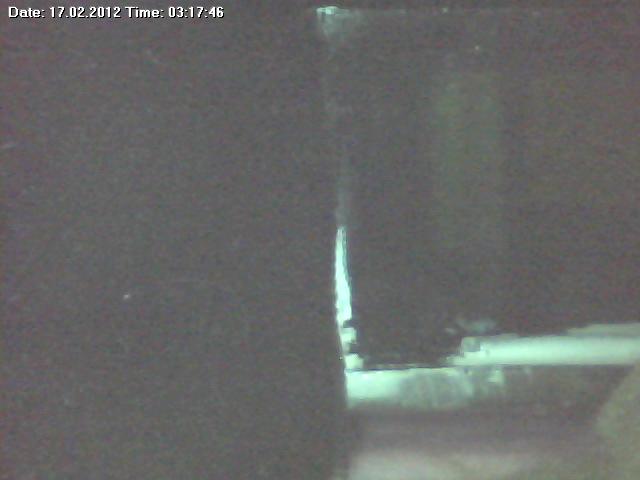

Supplement: Supplementary file 1 — Supplementary material [file mmc1.zip › Supplementary files/Supplementary Figure 1098.jpg]

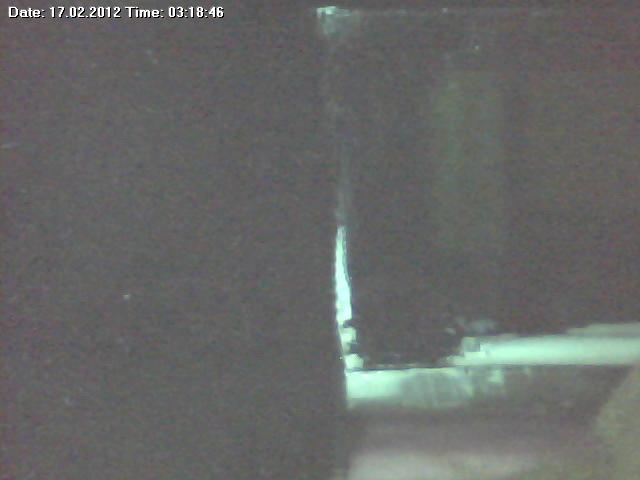

Supplement: Supplementary file 1 — Supplementary material [file mmc1.zip › Supplementary files/Supplementary Figure 1099.jpg]

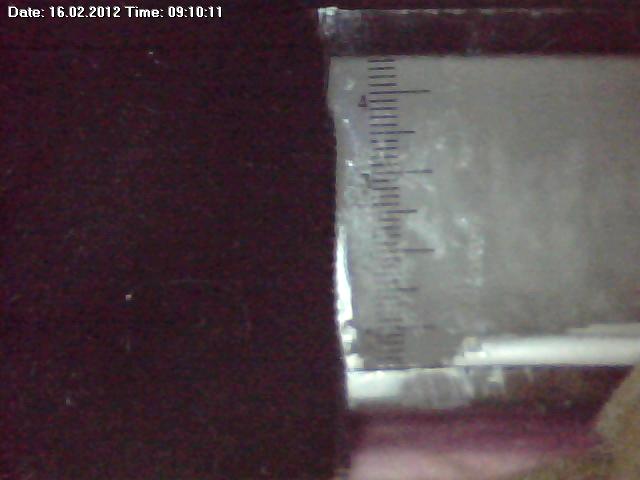

Supplement: Supplementary file 1 — Supplementary material [file mmc1.zip › Supplementary files/Supplementary Figure 11.jpg]

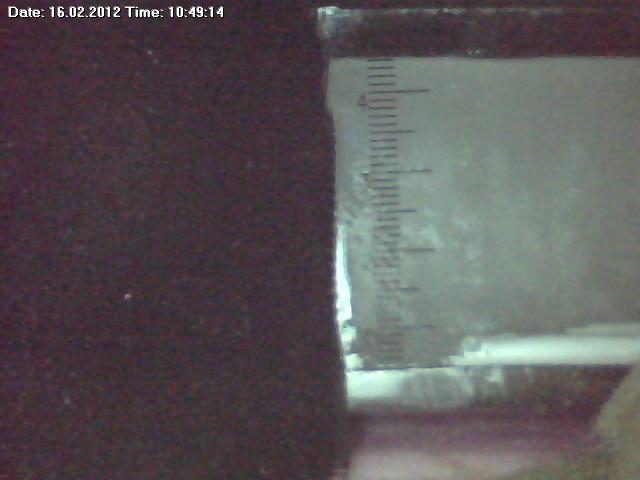

Supplement: Supplementary file 1 — Supplementary material [file mmc1.zip › Supplementary files/Supplementary Figure 110.jpg]

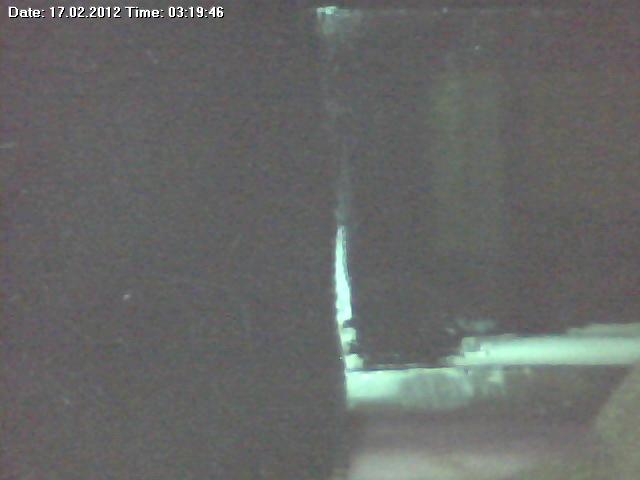

Supplement: Supplementary file 1 — Supplementary material [file mmc1.zip › Supplementary files/Supplementary Figure 1100.jpg]

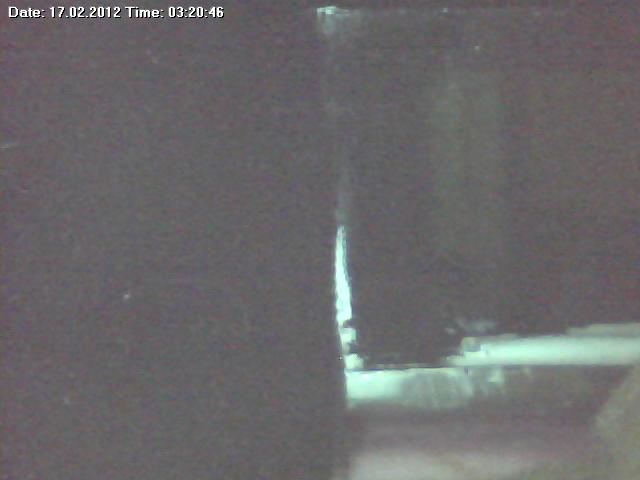

Supplement: Supplementary file 1 — Supplementary material [file mmc1.zip › Supplementary files/Supplementary Figure 1101.jpg]

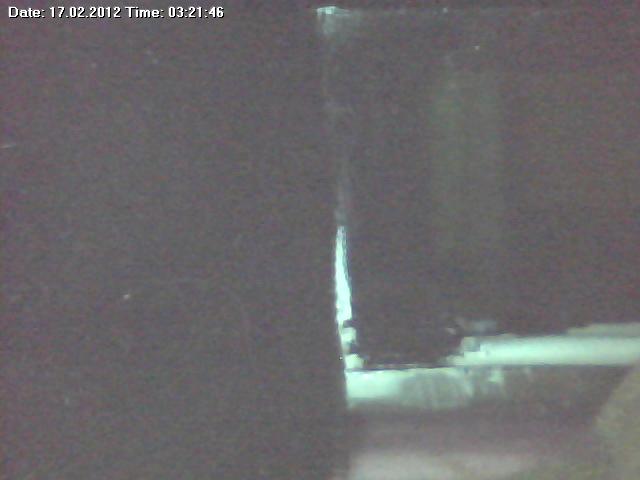

Supplement: Supplementary file 1 — Supplementary material [file mmc1.zip › Supplementary files/Supplementary Figure 1102.jpg]

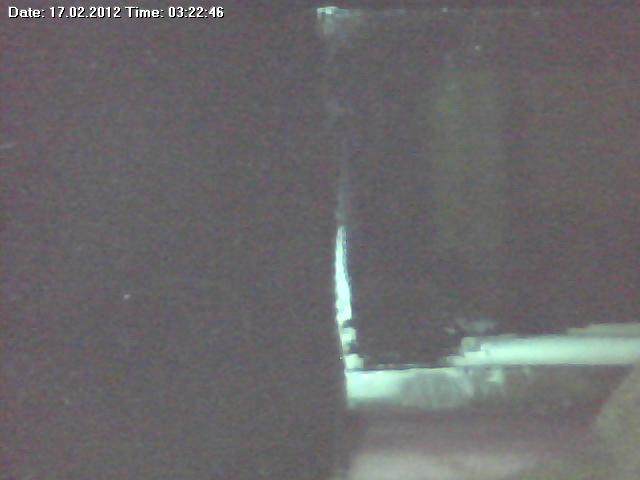

Supplement: Supplementary file 1 — Supplementary material [file mmc1.zip › Supplementary files/Supplementary Figure 1103.jpg]

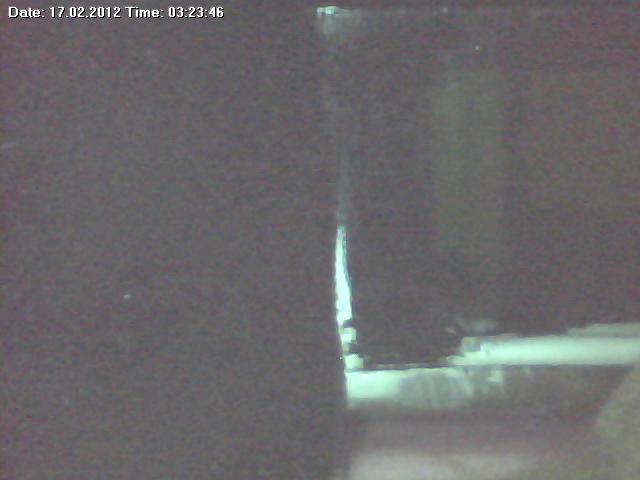

Supplement: Supplementary file 1 — Supplementary material [file mmc1.zip › Supplementary files/Supplementary Figure 1104.jpg]

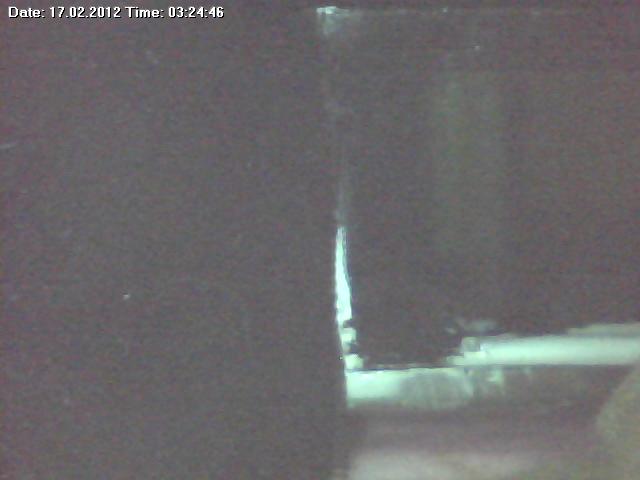

Supplement: Supplementary file 1 — Supplementary material [file mmc1.zip › Supplementary files/Supplementary Figure 1105.jpg]

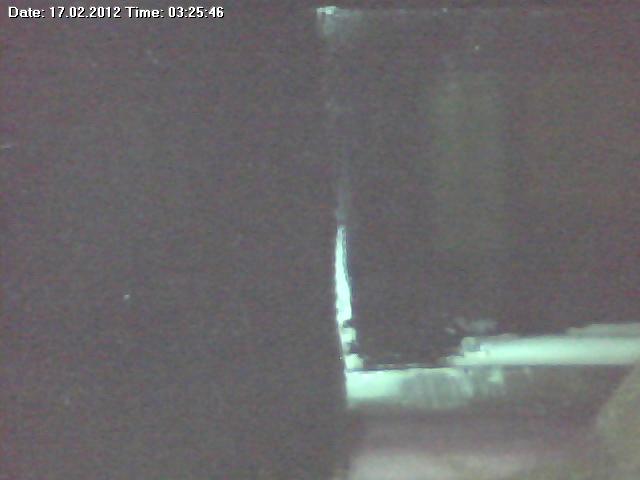

Supplement: Supplementary file 1 — Supplementary material [file mmc1.zip › Supplementary files/Supplementary Figure 1106.jpg]

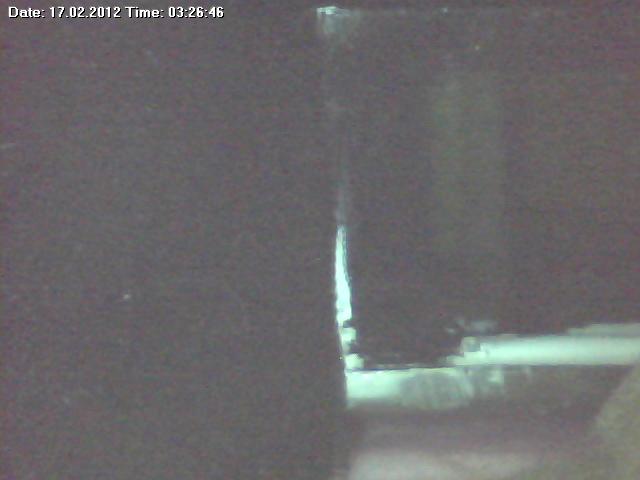

Supplement: Supplementary file 1 — Supplementary material [file mmc1.zip › Supplementary files/Supplementary Figure 1107.jpg]

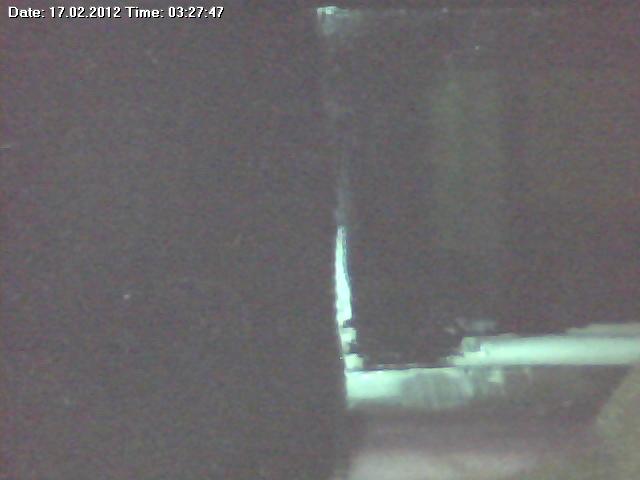

Supplement: Supplementary file 1 — Supplementary material [file mmc1.zip › Supplementary files/Supplementary Figure 1108.jpg]

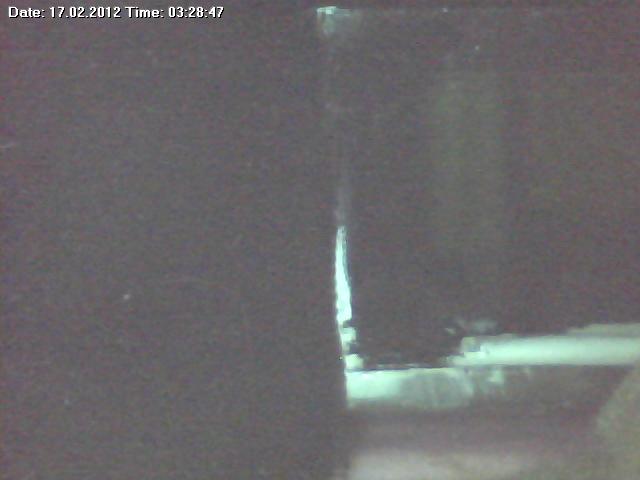

Supplement: Supplementary file 1 — Supplementary material [file mmc1.zip › Supplementary files/Supplementary Figure 1109.jpg]

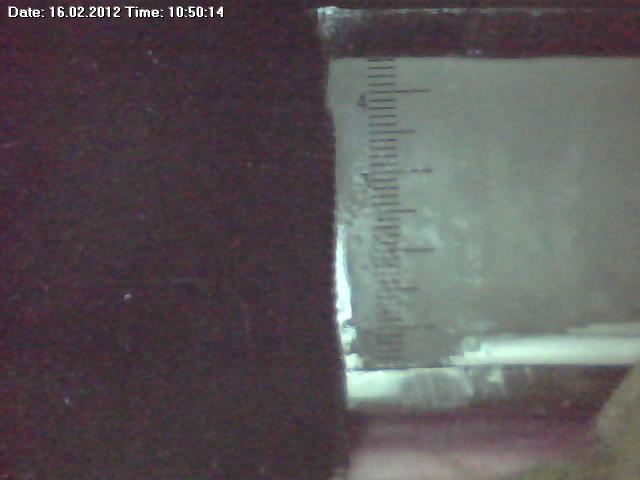

Supplement: Supplementary file 1 — Supplementary material [file mmc1.zip › Supplementary files/Supplementary Figure 111.jpg]

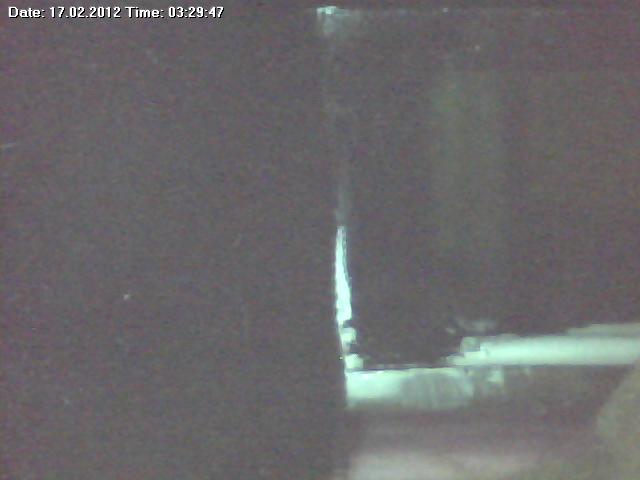

Supplement: Supplementary file 1 — Supplementary material [file mmc1.zip › Supplementary files/Supplementary Figure 1110.jpg]

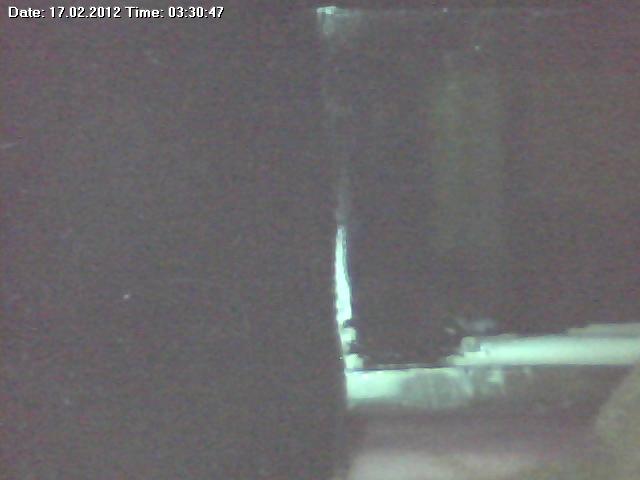

Supplement: Supplementary file 1 — Supplementary material [file mmc1.zip › Supplementary files/Supplementary Figure 1111.jpg]

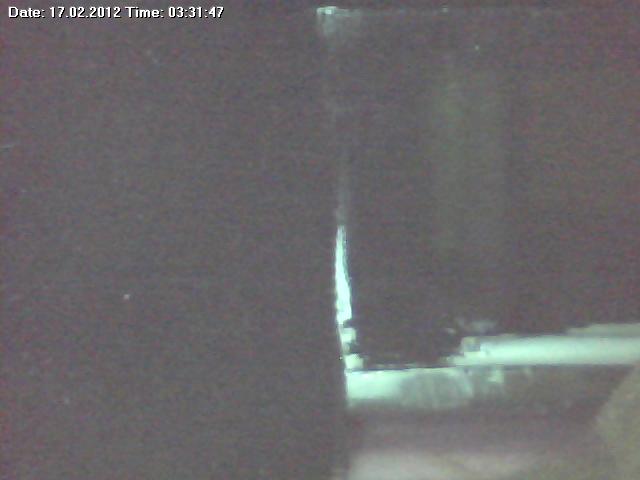

Supplement: Supplementary file 1 — Supplementary material [file mmc1.zip › Supplementary files/Supplementary Figure 1112.jpg]

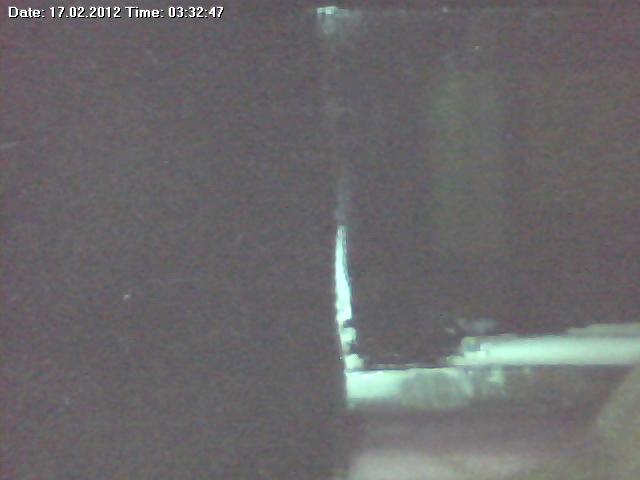

Supplement: Supplementary file 1 — Supplementary material [file mmc1.zip › Supplementary files/Supplementary Figure 1113.jpg]

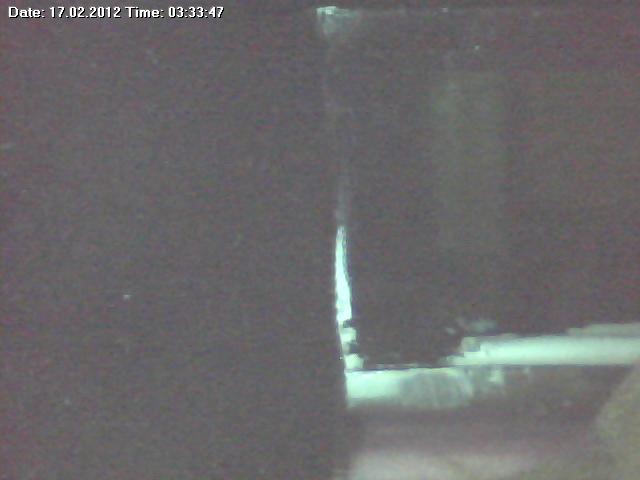

Supplement: Supplementary file 1 — Supplementary material [file mmc1.zip › Supplementary files/Supplementary Figure 1114.jpg]

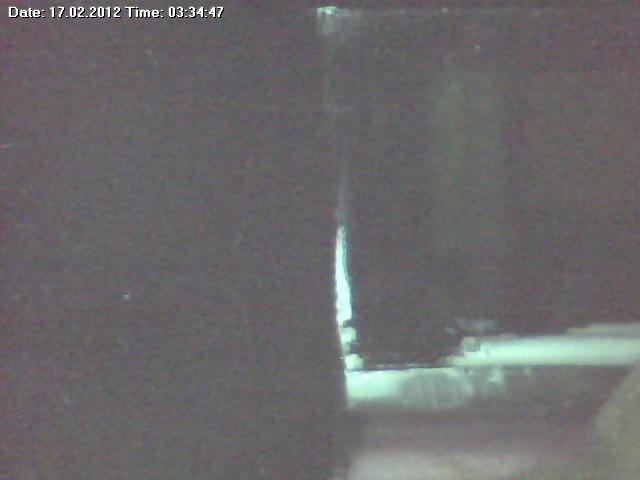

Supplement: Supplementary file 1 — Supplementary material [file mmc1.zip › Supplementary files/Supplementary Figure 1115.jpg]

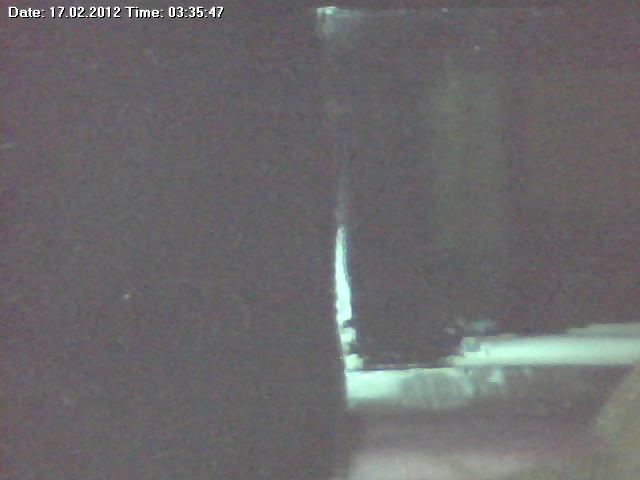

Supplement: Supplementary file 1 — Supplementary material [file mmc1.zip › Supplementary files/Supplementary Figure 1116.jpg]

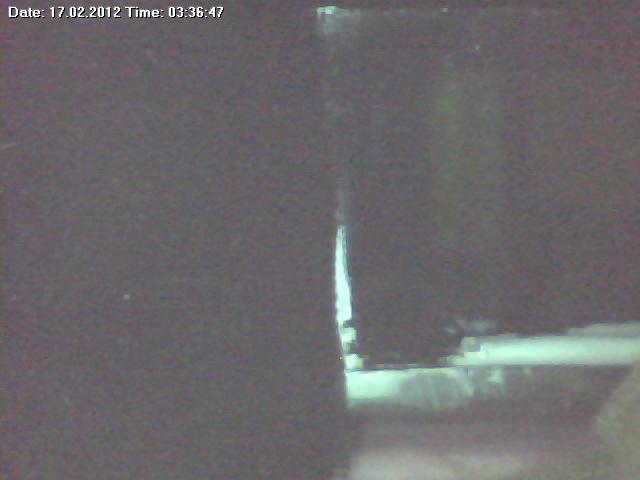

Supplement: Supplementary file 1 — Supplementary material [file mmc1.zip › Supplementary files/Supplementary Figure 1117.jpg]

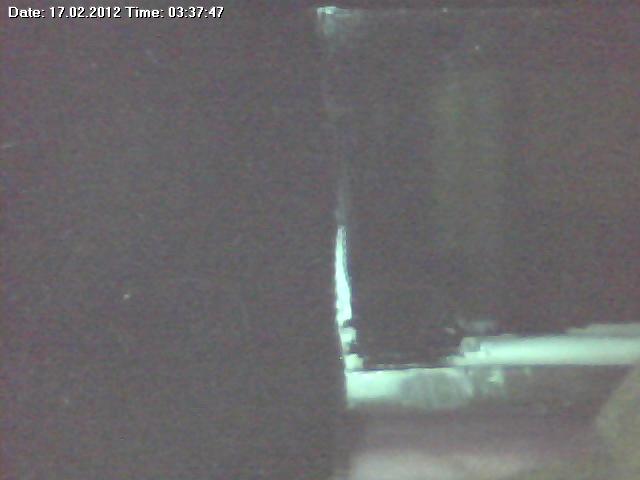

Supplement: Supplementary file 1 — Supplementary material [file mmc1.zip › Supplementary files/Supplementary Figure 1118.jpg]

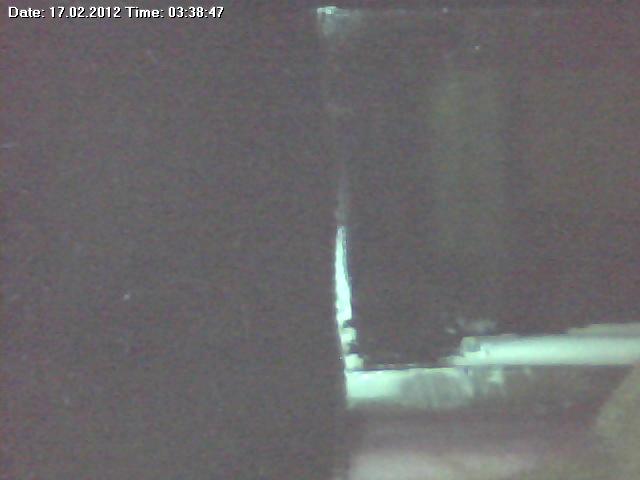

Supplement: Supplementary file 1 — Supplementary material [file mmc1.zip › Supplementary files/Supplementary Figure 1119.jpg]

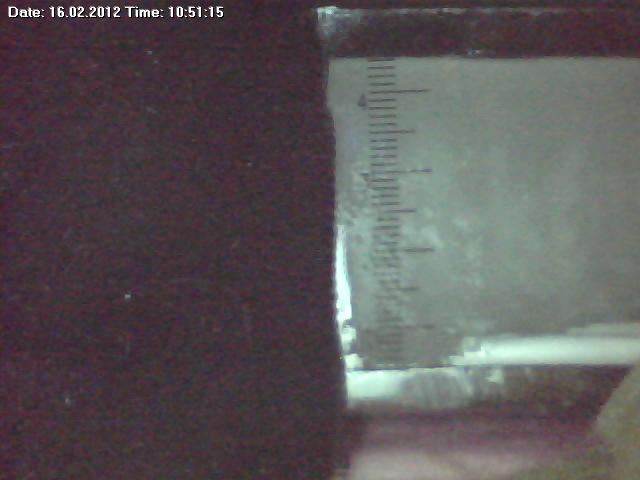

Supplement: Supplementary file 1 — Supplementary material [file mmc1.zip › Supplementary files/Supplementary Figure 112.jpg]

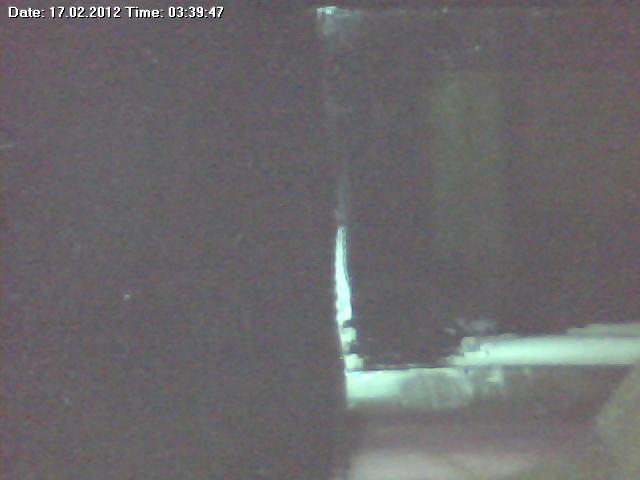

Supplement: Supplementary file 1 — Supplementary material [file mmc1.zip › Supplementary files/Supplementary Figure 1120.jpg]

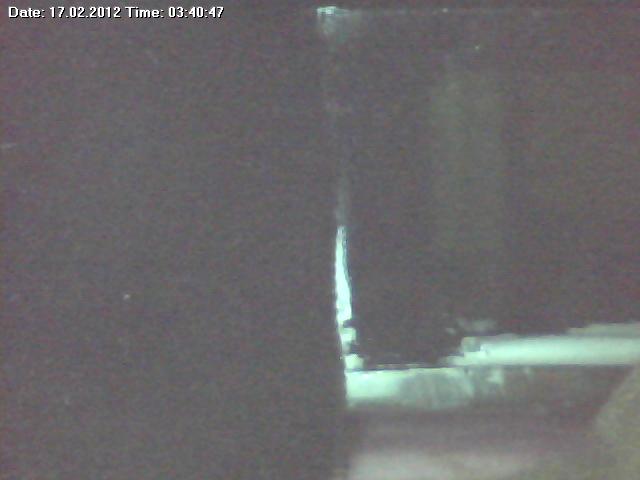

Supplement: Supplementary file 1 — Supplementary material [file mmc1.zip › Supplementary files/Supplementary Figure 1121.jpg]

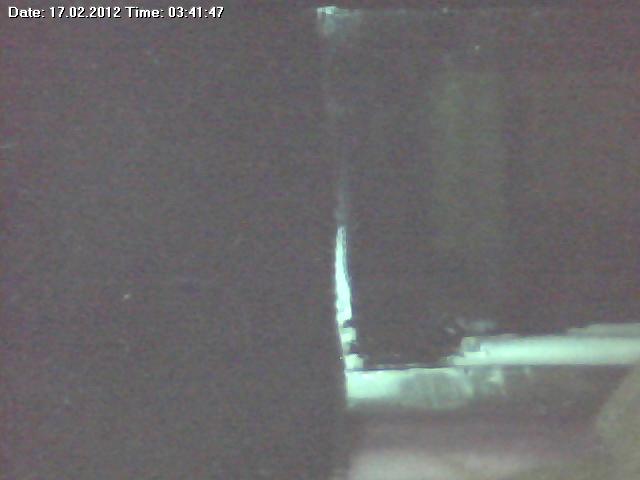

Supplement: Supplementary file 1 — Supplementary material [file mmc1.zip › Supplementary files/Supplementary Figure 1122.jpg]

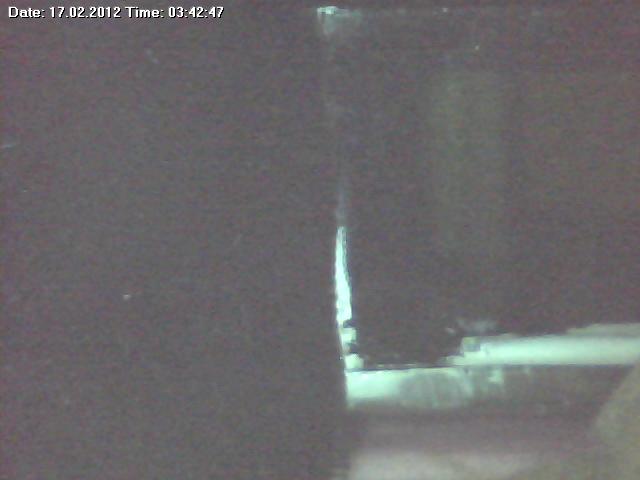

Supplement: Supplementary file 1 — Supplementary material [file mmc1.zip › Supplementary files/Supplementary Figure 1123.jpg]

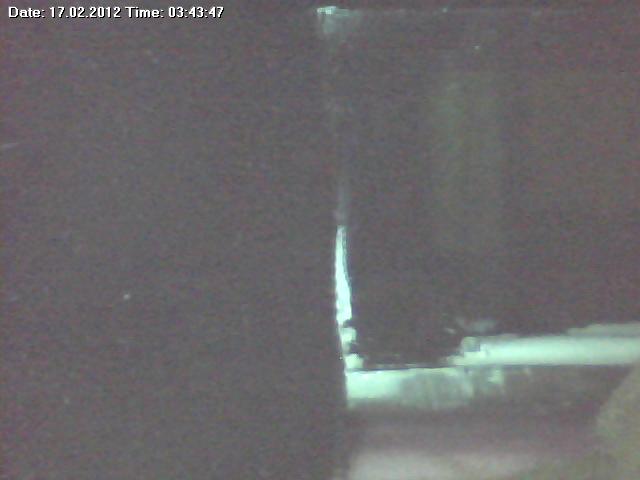

Supplement: Supplementary file 1 — Supplementary material [file mmc1.zip › Supplementary files/Supplementary Figure 1124.jpg]

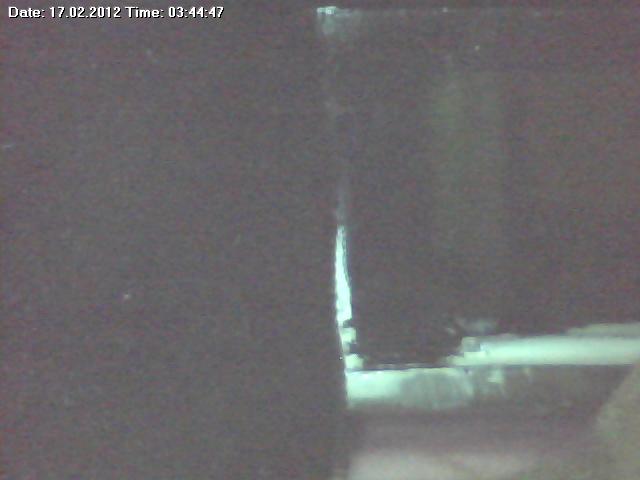

Supplement: Supplementary file 1 — Supplementary material [file mmc1.zip › Supplementary files/Supplementary Figure 1125.jpg]

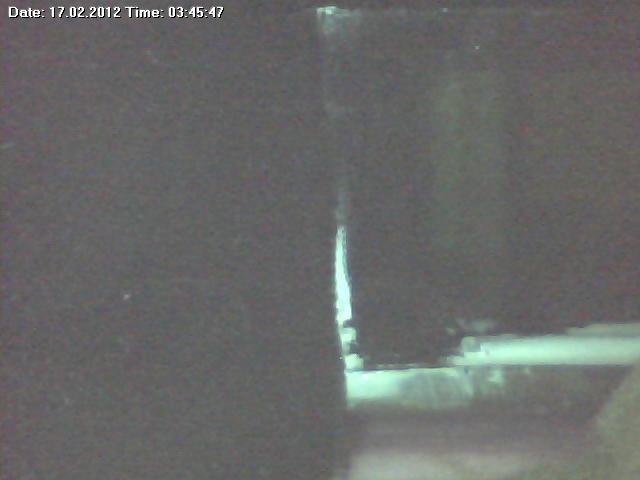

Supplement: Supplementary file 1 — Supplementary material [file mmc1.zip › Supplementary files/Supplementary Figure 1126.jpg]

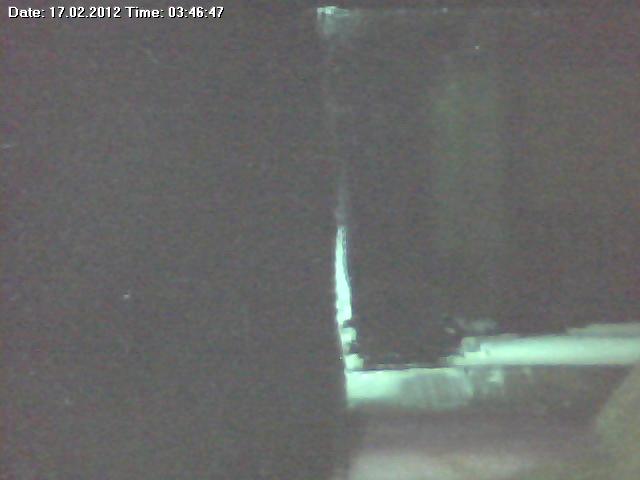

Supplement: Supplementary file 1 — Supplementary material [file mmc1.zip › Supplementary files/Supplementary Figure 1127.jpg]

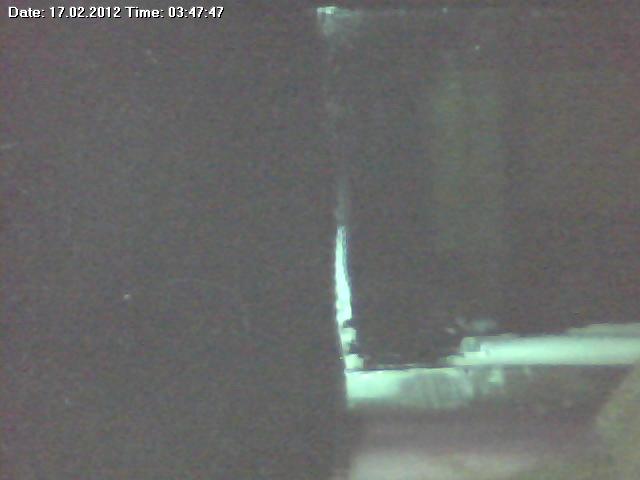

Supplement: Supplementary file 1 — Supplementary material [file mmc1.zip › Supplementary files/Supplementary Figure 1128.jpg]

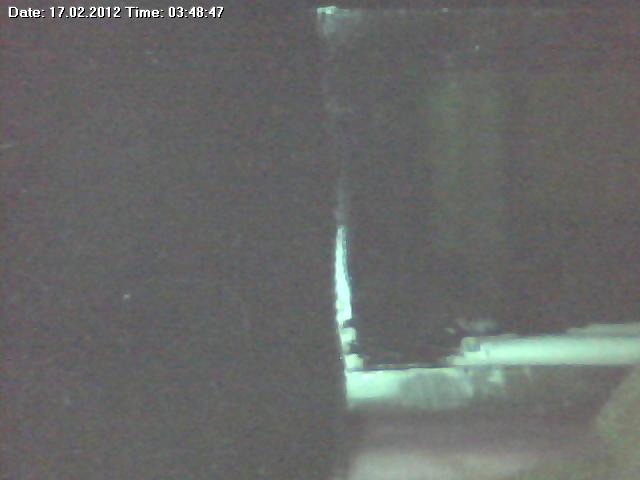

Supplement: Supplementary file 1 — Supplementary material [file mmc1.zip › Supplementary files/Supplementary Figure 1129.jpg]

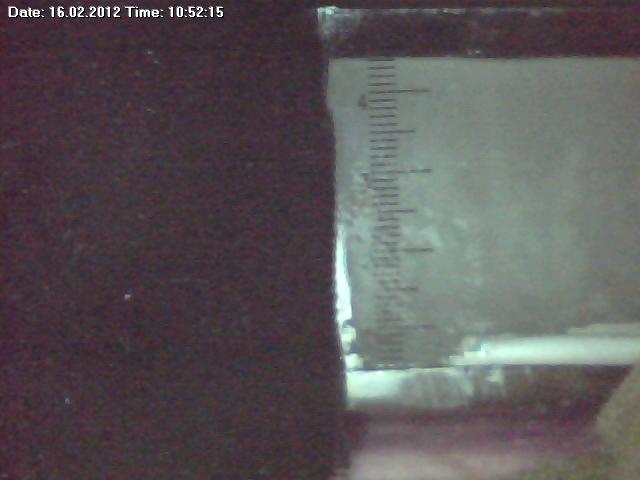

Supplement: Supplementary file 1 — Supplementary material [file mmc1.zip › Supplementary files/Supplementary Figure 113.jpg]

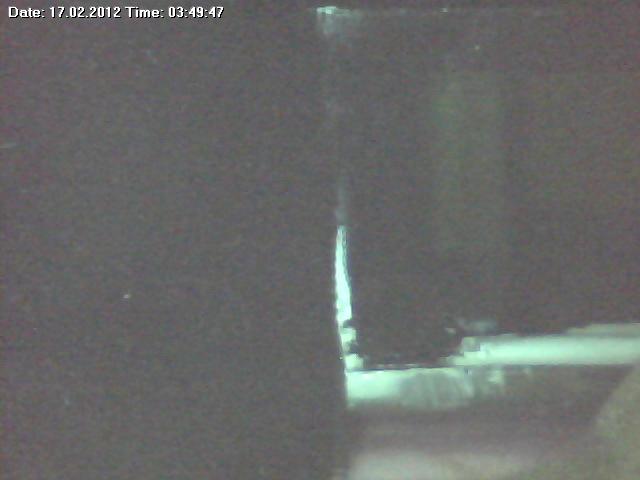

Supplement: Supplementary file 1 — Supplementary material [file mmc1.zip › Supplementary files/Supplementary Figure 1130.jpg]

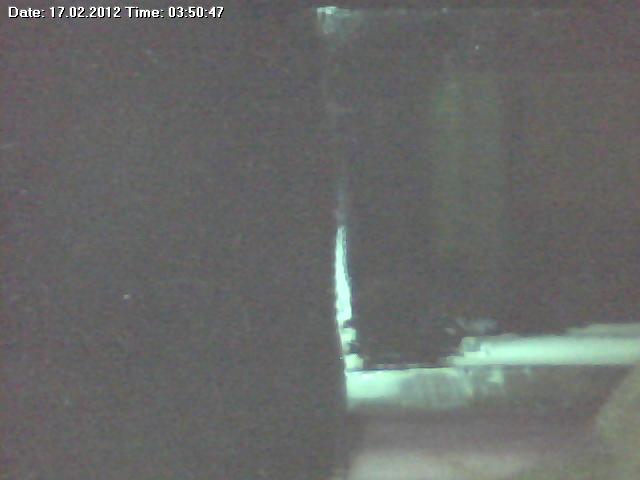

Supplement: Supplementary file 1 — Supplementary material [file mmc1.zip › Supplementary files/Supplementary Figure 1131.jpg]

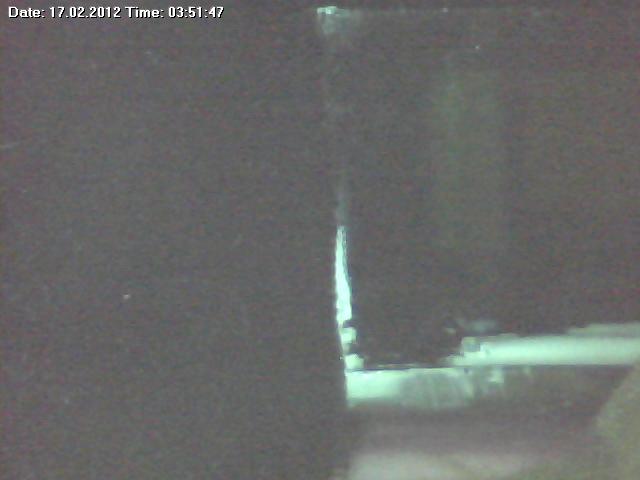

Supplement: Supplementary file 1 — Supplementary material [file mmc1.zip › Supplementary files/Supplementary Figure 1132.jpg]

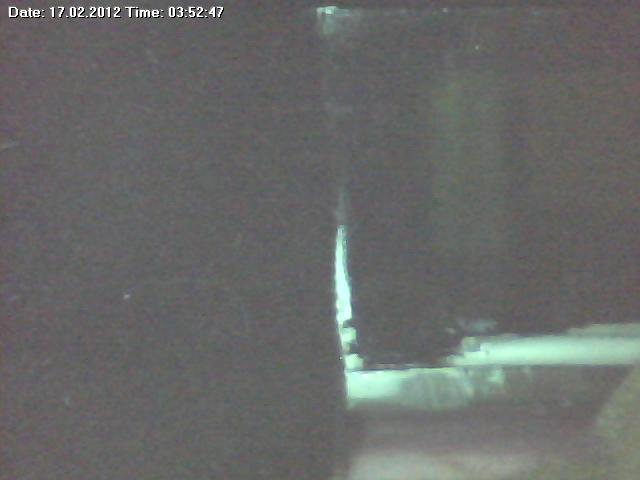

Supplement: Supplementary file 1 — Supplementary material [file mmc1.zip › Supplementary files/Supplementary Figure 1133.jpg]

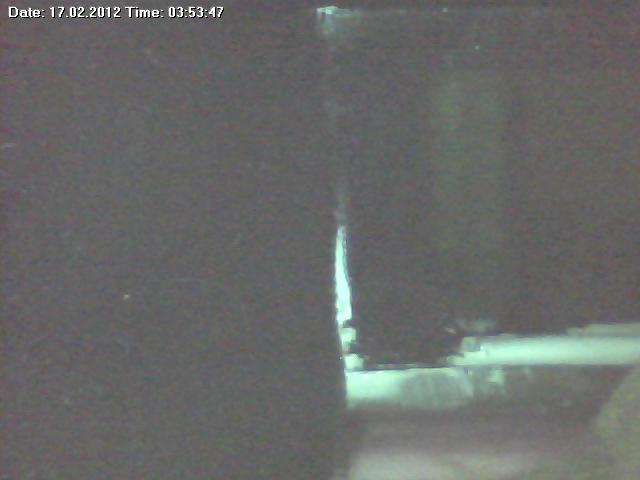

Supplement: Supplementary file 1 — Supplementary material [file mmc1.zip › Supplementary files/Supplementary Figure 1134.jpg]

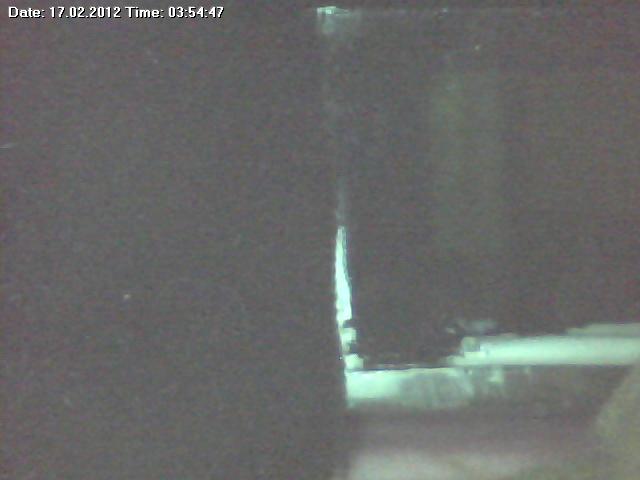

Supplement: Supplementary file 1 — Supplementary material [file mmc1.zip › Supplementary files/Supplementary Figure 1135.jpg]

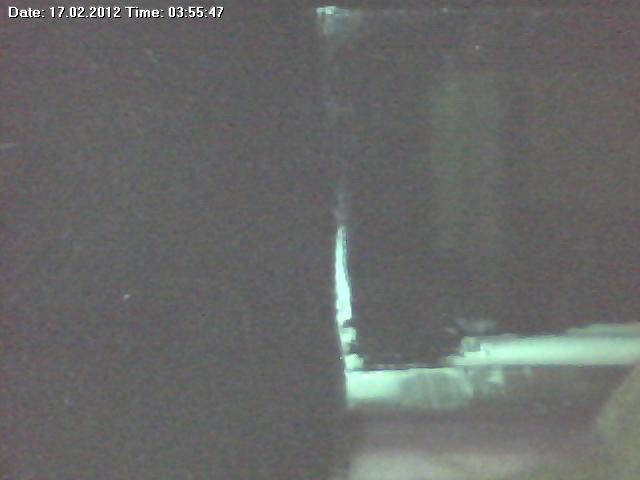

Supplement: Supplementary file 1 — Supplementary material [file mmc1.zip › Supplementary files/Supplementary Figure 1136.jpg]

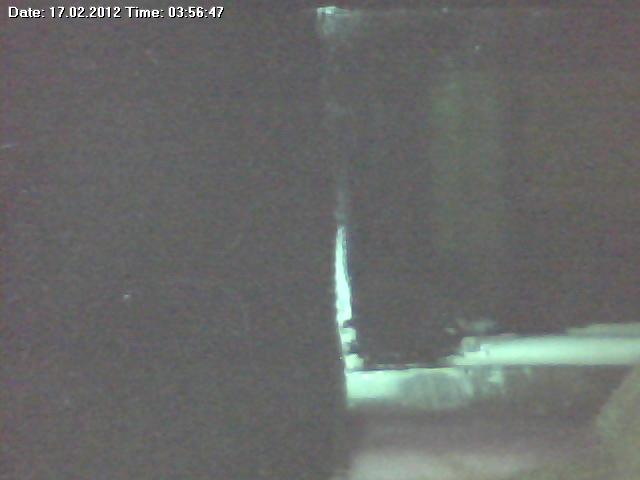

Supplement: Supplementary file 1 — Supplementary material [file mmc1.zip › Supplementary files/Supplementary Figure 1137.jpg]

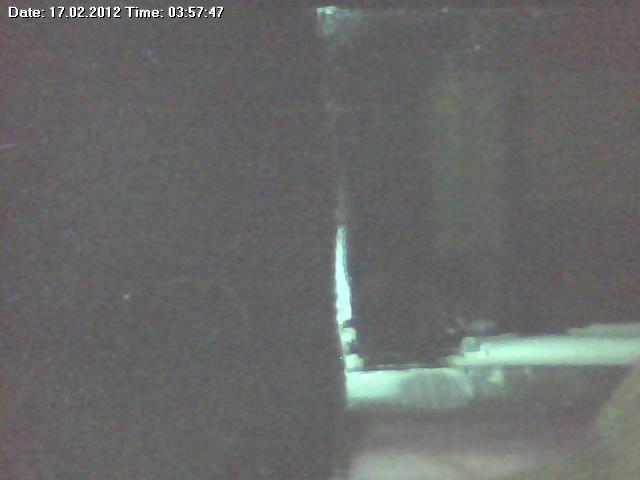

Supplement: Supplementary file 1 — Supplementary material [file mmc1.zip › Supplementary files/Supplementary Figure 1138.jpg]

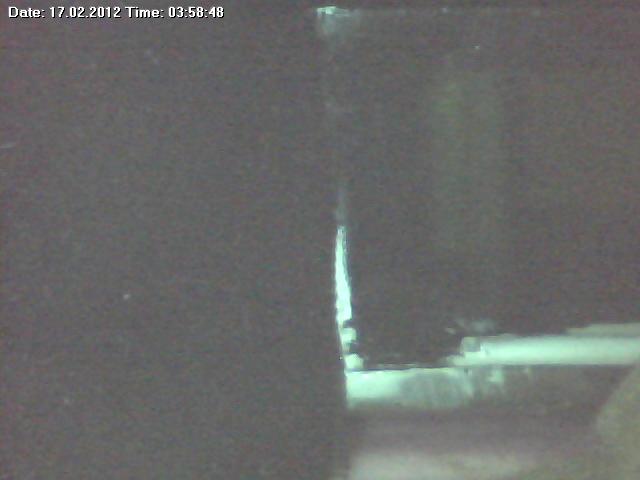

Supplement: Supplementary file 1 — Supplementary material [file mmc1.zip › Supplementary files/Supplementary Figure 1139.jpg]

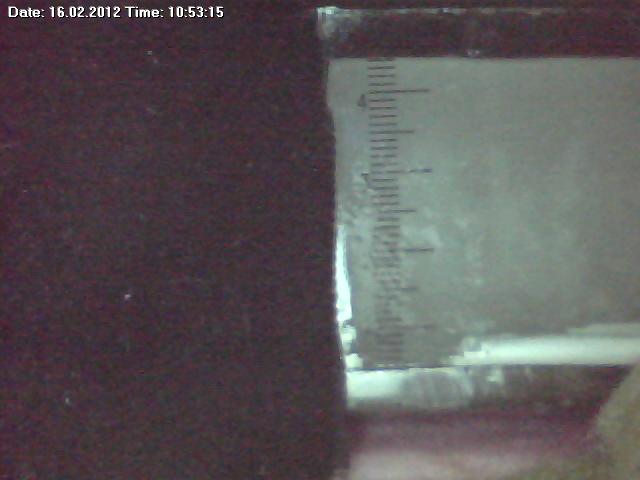

Supplement: Supplementary file 1 — Supplementary material [file mmc1.zip › Supplementary files/Supplementary Figure 114.jpg]

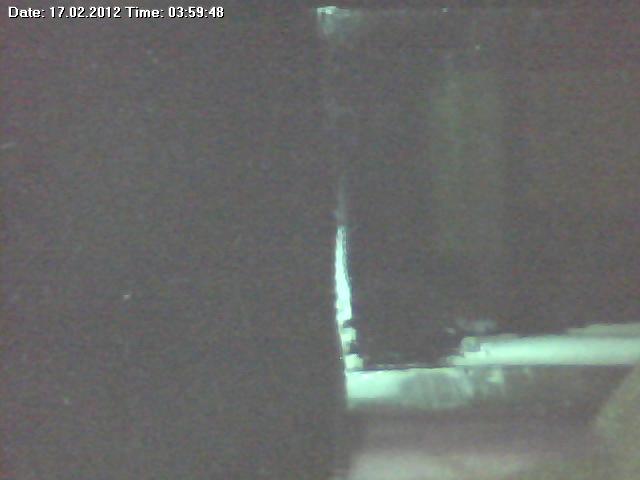

Supplement: Supplementary file 1 — Supplementary material [file mmc1.zip › Supplementary files/Supplementary Figure 1140.jpg]

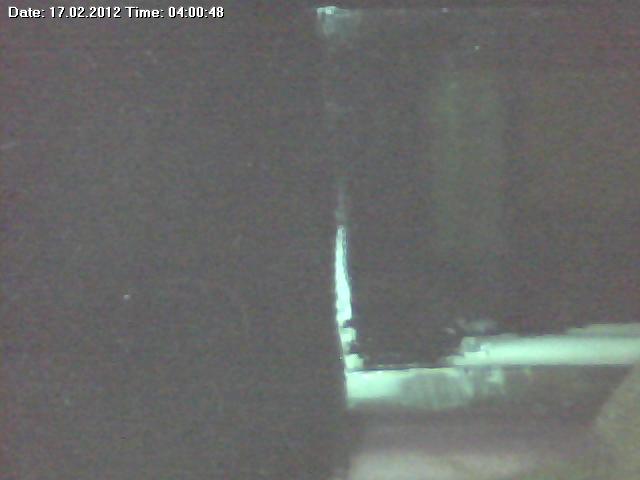

Supplement: Supplementary file 1 — Supplementary material [file mmc1.zip › Supplementary files/Supplementary Figure 1141.jpg]

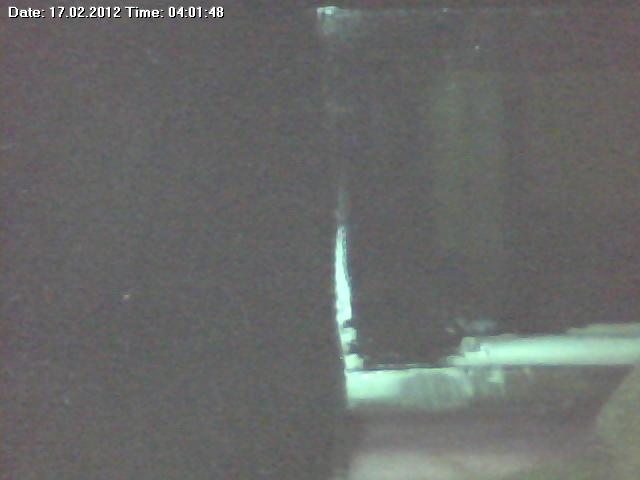

Supplement: Supplementary file 1 — Supplementary material [file mmc1.zip › Supplementary files/Supplementary Figure 1142.jpg]

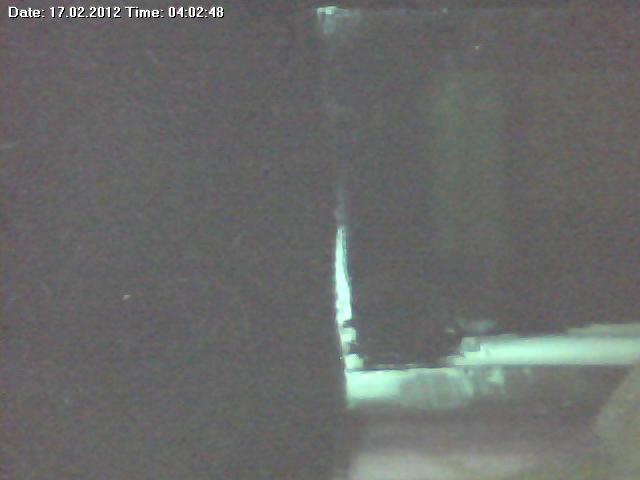

Supplement: Supplementary file 1 — Supplementary material [file mmc1.zip › Supplementary files/Supplementary Figure 1143.jpg]

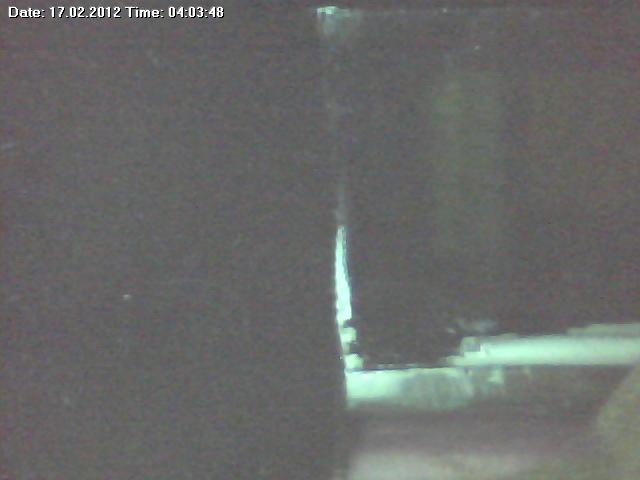

Supplement: Supplementary file 1 — Supplementary material [file mmc1.zip › Supplementary files/Supplementary Figure 1144.jpg]

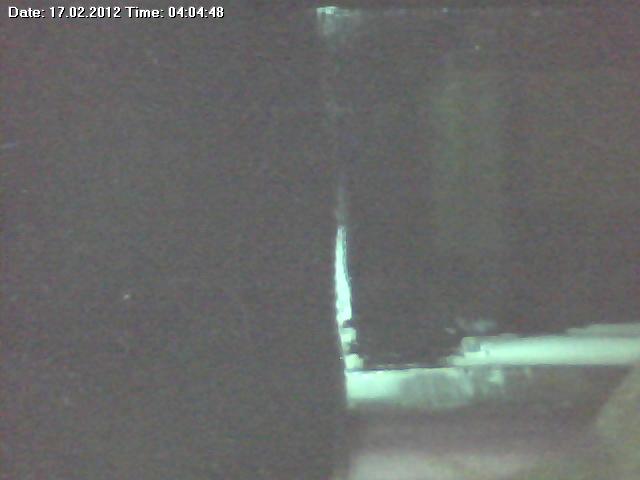

Supplement: Supplementary file 1 — Supplementary material [file mmc1.zip › Supplementary files/Supplementary Figure 1145.jpg]

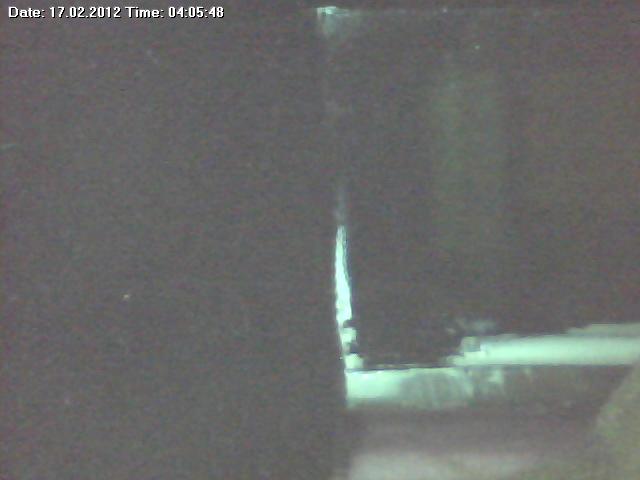

Supplement: Supplementary file 1 — Supplementary material [file mmc1.zip › Supplementary files/Supplementary Figure 1146.jpg]

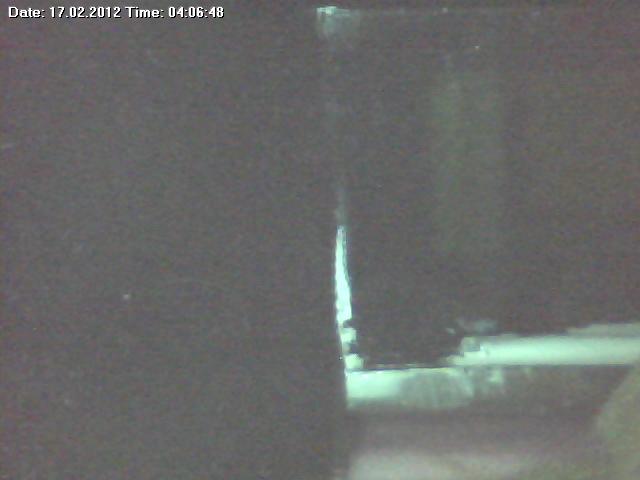

Supplement: Supplementary file 1 — Supplementary material [file mmc1.zip › Supplementary files/Supplementary Figure 1147.jpg]

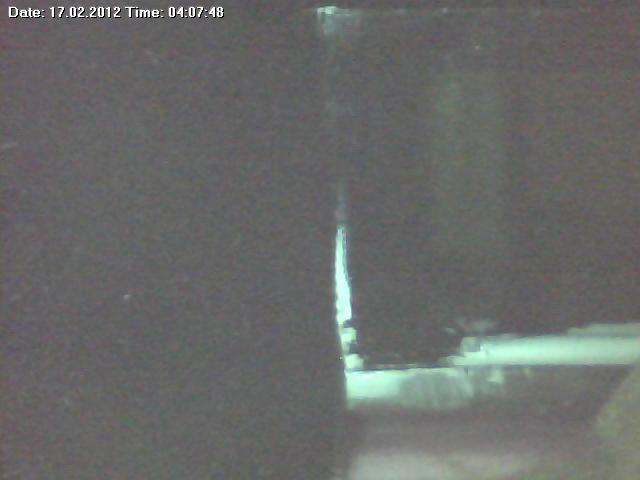

Supplement: Supplementary file 1 — Supplementary material [file mmc1.zip › Supplementary files/Supplementary Figure 1148.jpg]

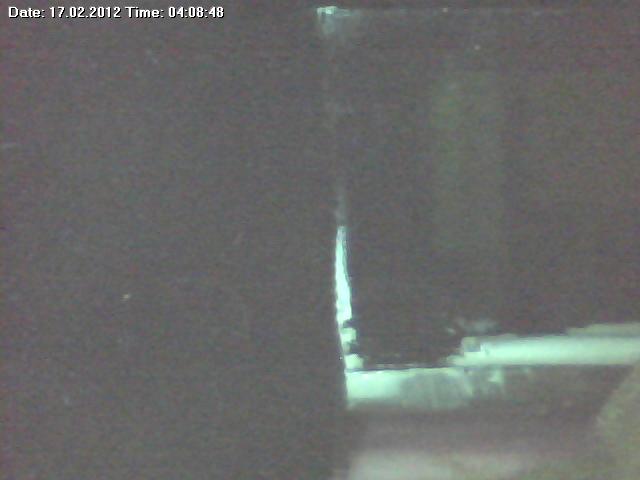

Supplement: Supplementary file 1 — Supplementary material [file mmc1.zip › Supplementary files/Supplementary Figure 1149.jpg]

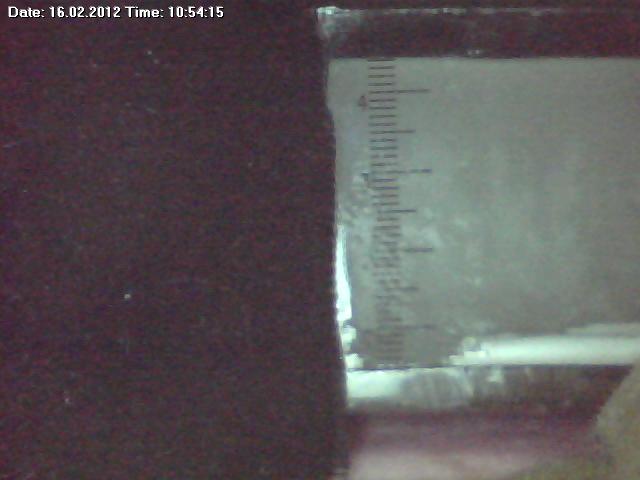

Supplement: Supplementary file 1 — Supplementary material [file mmc1.zip › Supplementary files/Supplementary Figure 115.jpg]

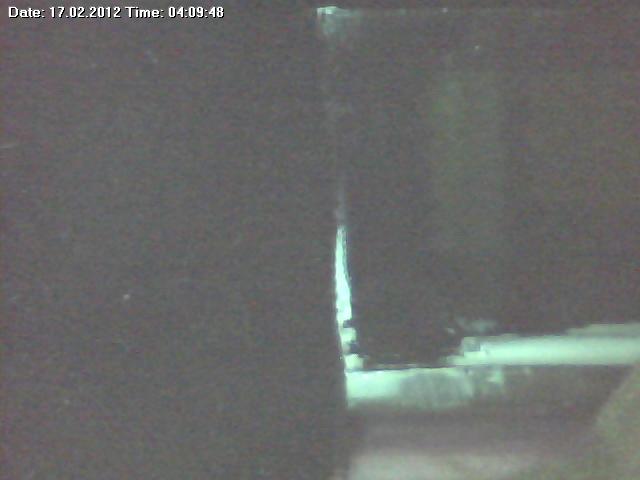

Supplement: Supplementary file 1 — Supplementary material [file mmc1.zip › Supplementary files/Supplementary Figure 1150.jpg]

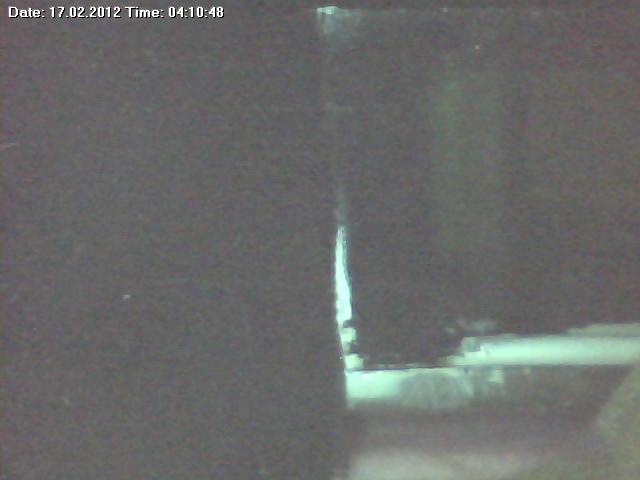

Supplement: Supplementary file 1 — Supplementary material [file mmc1.zip › Supplementary files/Supplementary Figure 1151.jpg]

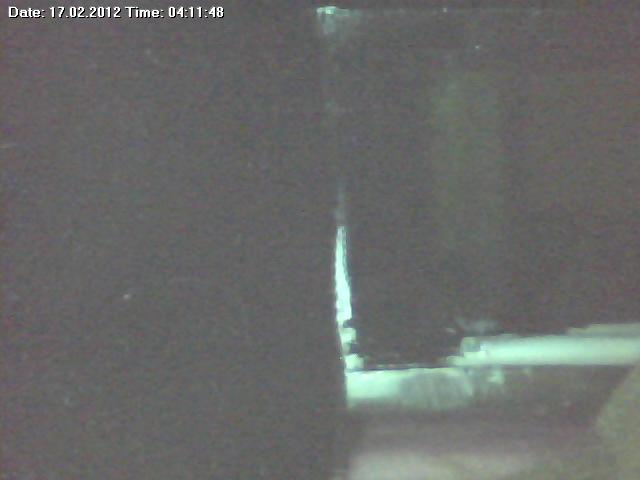

Supplement: Supplementary file 1 — Supplementary material [file mmc1.zip › Supplementary files/Supplementary Figure 1152.jpg]

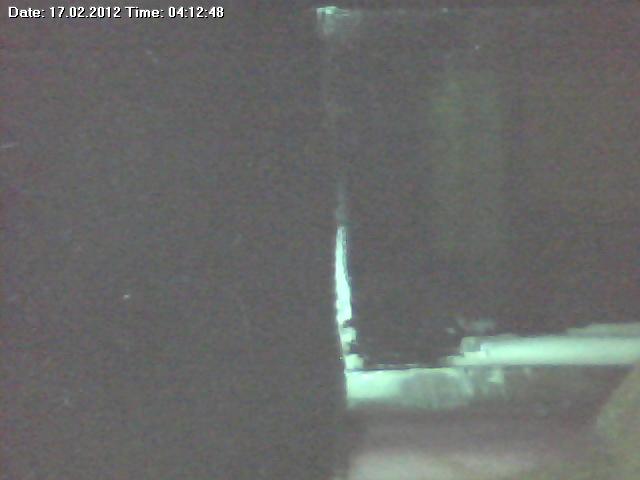

Supplement: Supplementary file 1 — Supplementary material [file mmc1.zip › Supplementary files/Supplementary Figure 1153.jpg]

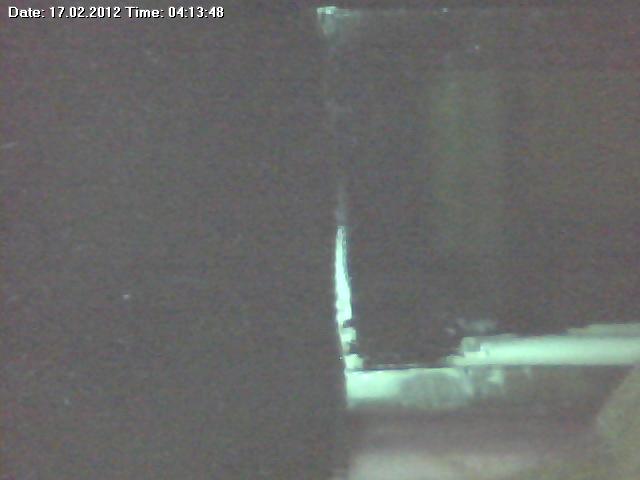

Supplement: Supplementary file 1 — Supplementary material [file mmc1.zip › Supplementary files/Supplementary Figure 1154.jpg]

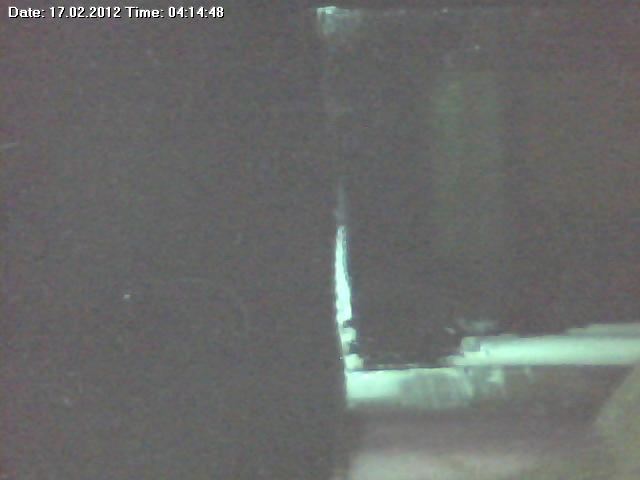

Supplement: Supplementary file 1 — Supplementary material [file mmc1.zip › Supplementary files/Supplementary Figure 1155.jpg]

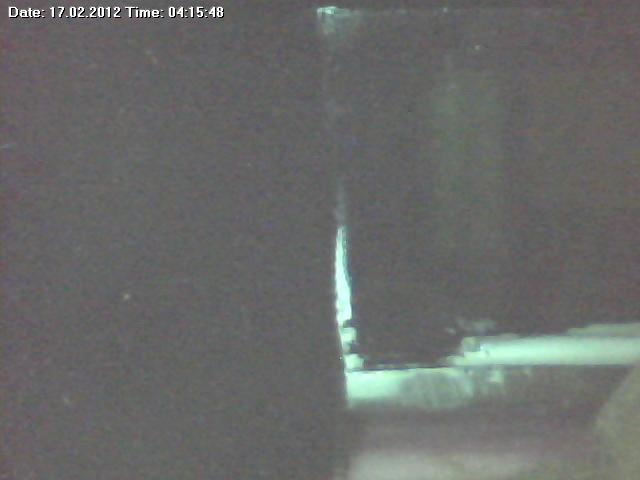

Supplement: Supplementary file 1 — Supplementary material [file mmc1.zip › Supplementary files/Supplementary Figure 1156.jpg]

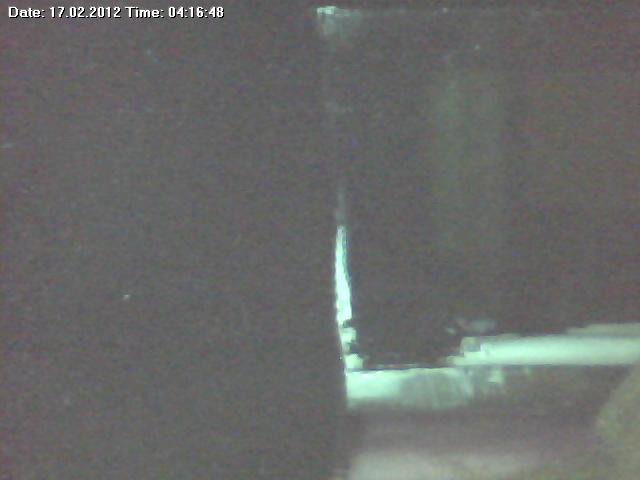

Supplement: Supplementary file 1 — Supplementary material [file mmc1.zip › Supplementary files/Supplementary Figure 1157.jpg]

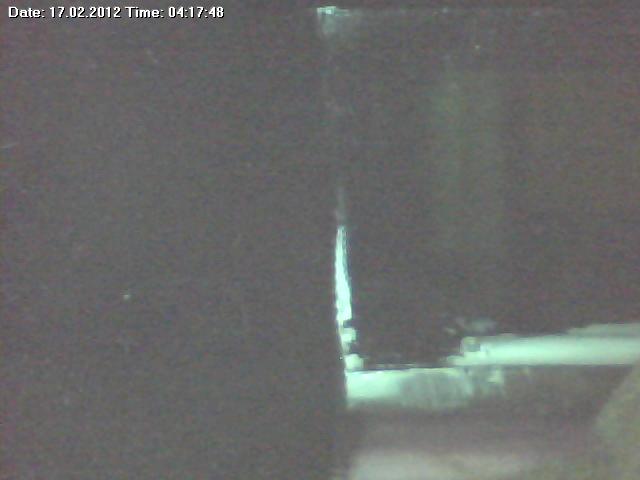

Supplement: Supplementary file 1 — Supplementary material [file mmc1.zip › Supplementary files/Supplementary Figure 1158.jpg]

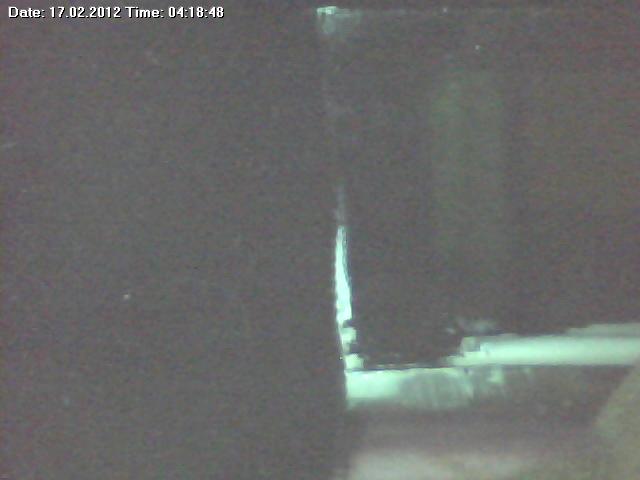

Supplement: Supplementary file 1 — Supplementary material [file mmc1.zip › Supplementary files/Supplementary Figure 1159.jpg]

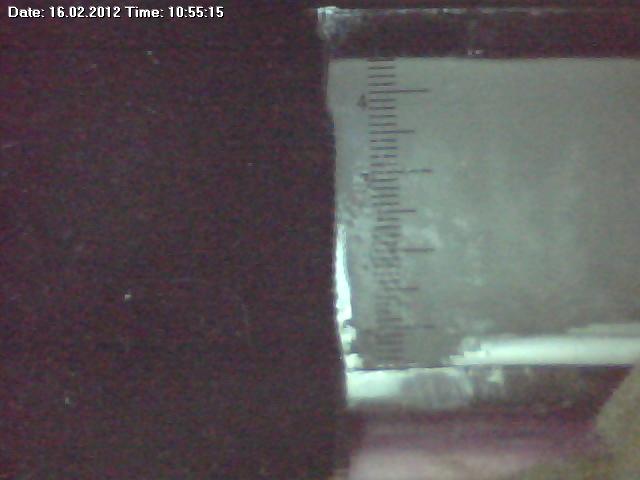

Supplement: Supplementary file 1 — Supplementary material [file mmc1.zip › Supplementary files/Supplementary Figure 116.jpg]

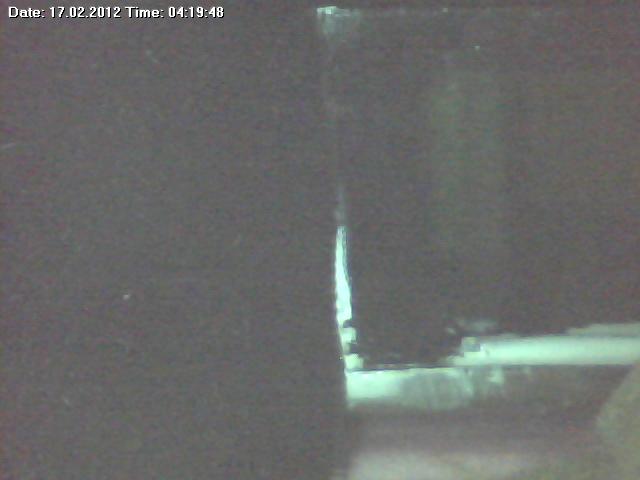

Supplement: Supplementary file 1 — Supplementary material [file mmc1.zip › Supplementary files/Supplementary Figure 1160.jpg]

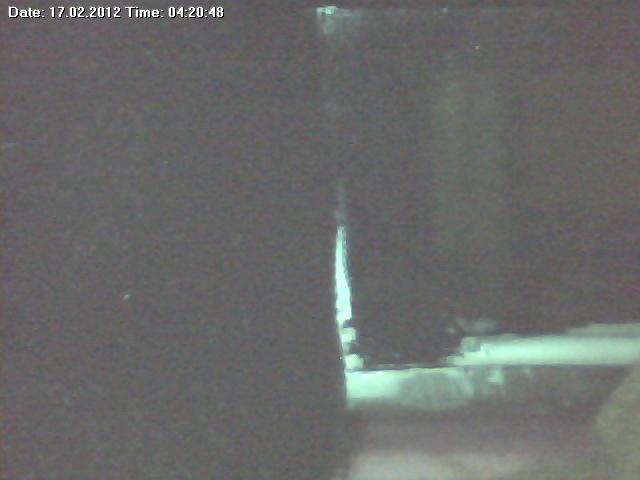

Supplement: Supplementary file 1 — Supplementary material [file mmc1.zip › Supplementary files/Supplementary Figure 1161.jpg]

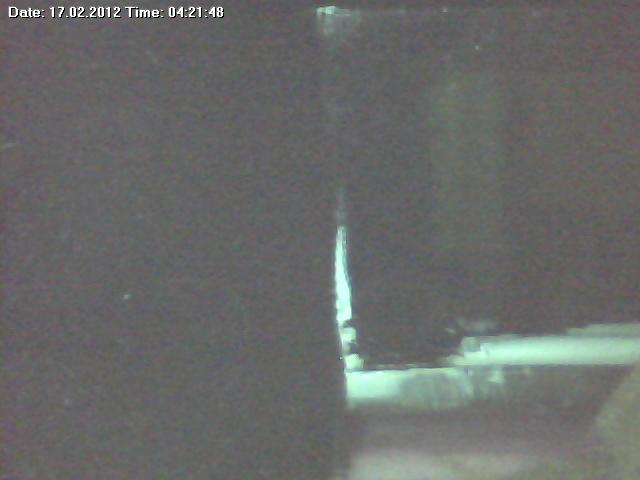

Supplement: Supplementary file 1 — Supplementary material [file mmc1.zip › Supplementary files/Supplementary Figure 1162.jpg]

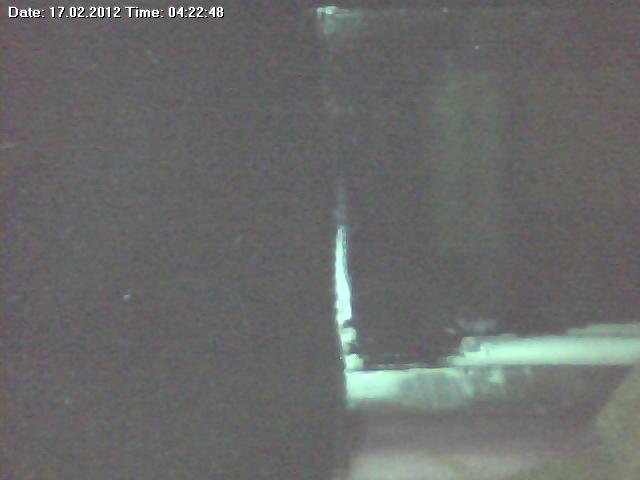

Supplement: Supplementary file 1 — Supplementary material [file mmc1.zip › Supplementary files/Supplementary Figure 1163.jpg]

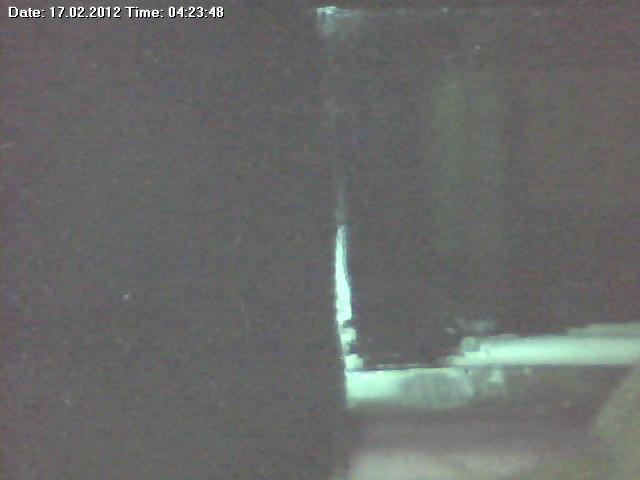

Supplement: Supplementary file 1 — Supplementary material [file mmc1.zip › Supplementary files/Supplementary Figure 1164.jpg]

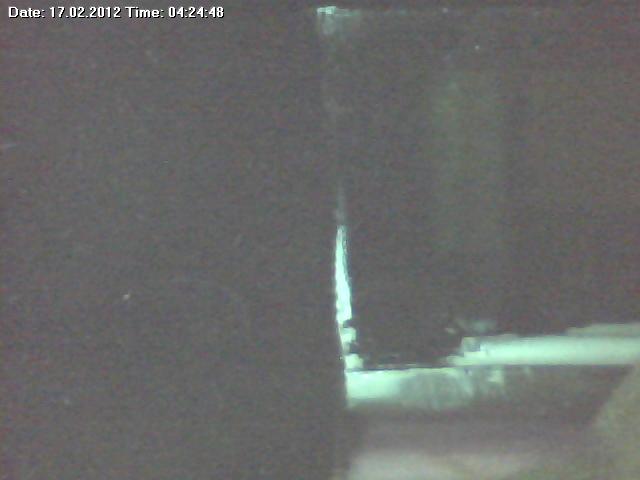

Supplement: Supplementary file 1 — Supplementary material [file mmc1.zip › Supplementary files/Supplementary Figure 1165.jpg]

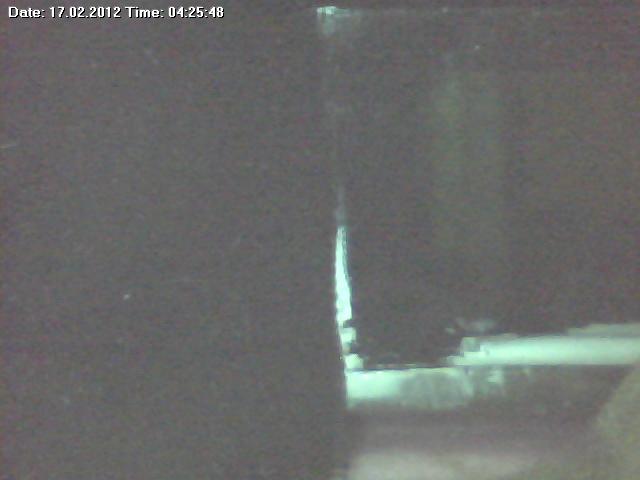

Supplement: Supplementary file 1 — Supplementary material [file mmc1.zip › Supplementary files/Supplementary Figure 1166.jpg]

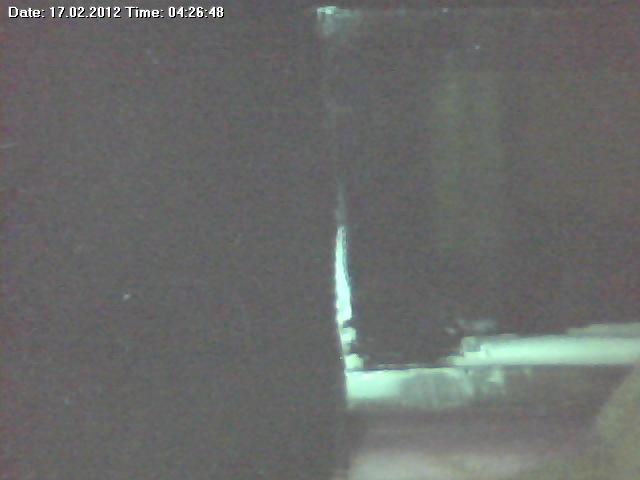

Supplement: Supplementary file 1 — Supplementary material [file mmc1.zip › Supplementary files/Supplementary Figure 1167.jpg]

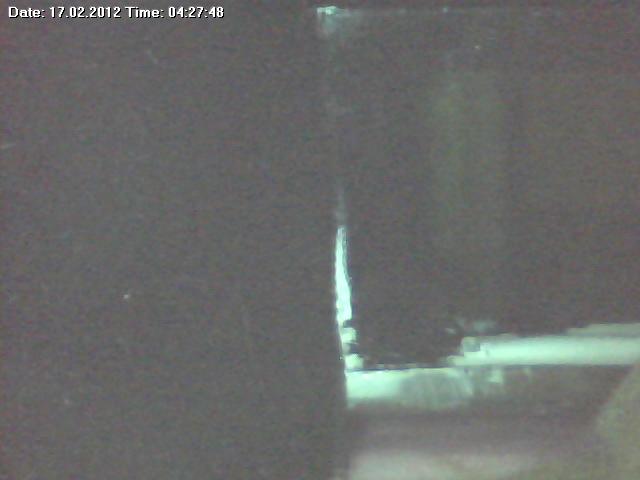

Supplement: Supplementary file 1 — Supplementary material [file mmc1.zip › Supplementary files/Supplementary Figure 1168.jpg]

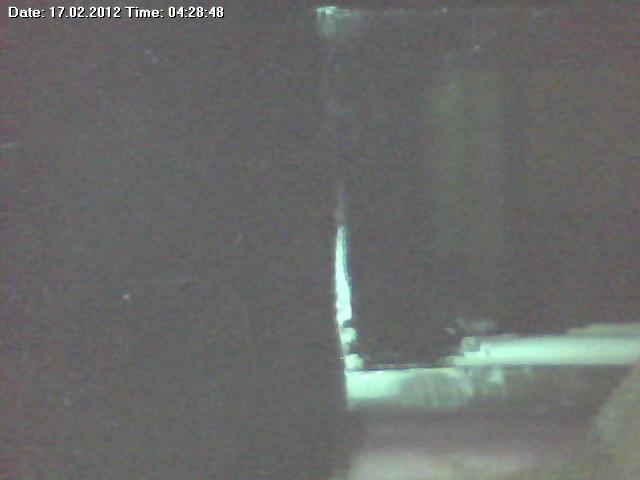

Supplement: Supplementary file 1 — Supplementary material [file mmc1.zip › Supplementary files/Supplementary Figure 1169.jpg]

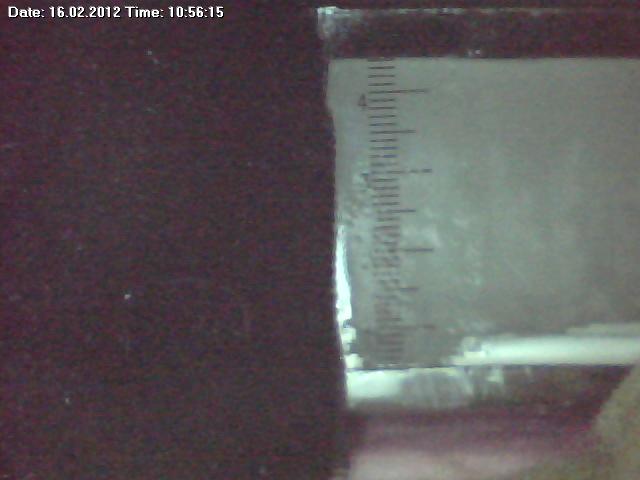

Supplement: Supplementary file 1 — Supplementary material [file mmc1.zip › Supplementary files/Supplementary Figure 117.jpg]

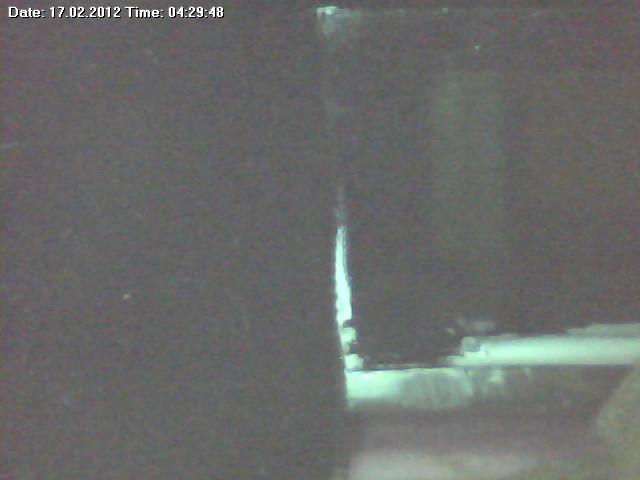

Supplement: Supplementary file 1 — Supplementary material [file mmc1.zip › Supplementary files/Supplementary Figure 1170.jpg]

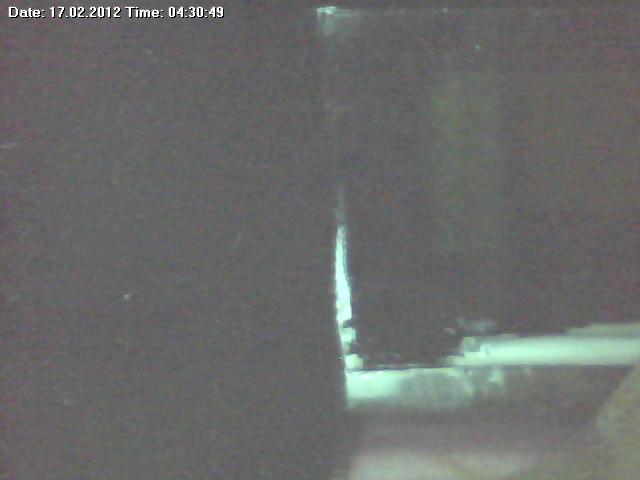

Supplement: Supplementary file 1 — Supplementary material [file mmc1.zip › Supplementary files/Supplementary Figure 1171.jpg]

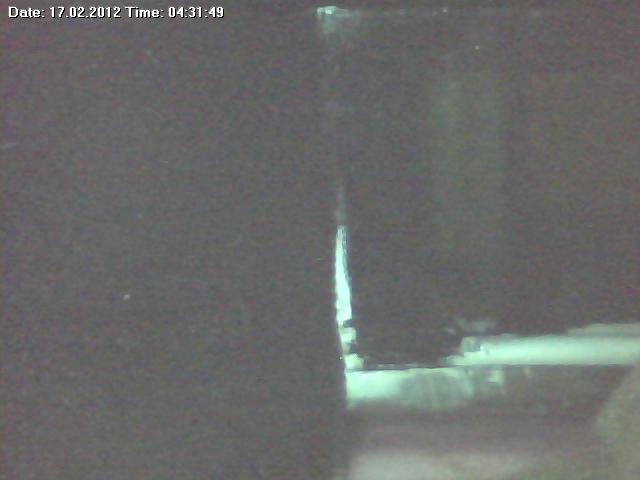

Supplement: Supplementary file 1 — Supplementary material [file mmc1.zip › Supplementary files/Supplementary Figure 1172.jpg]

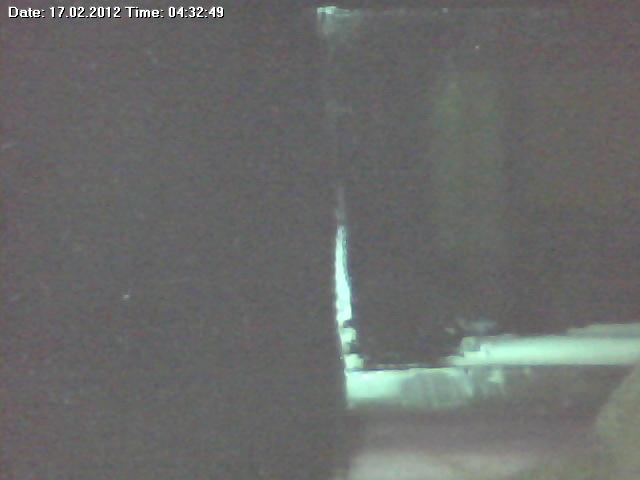

Supplement: Supplementary file 1 — Supplementary material [file mmc1.zip › Supplementary files/Supplementary Figure 1173.jpg]

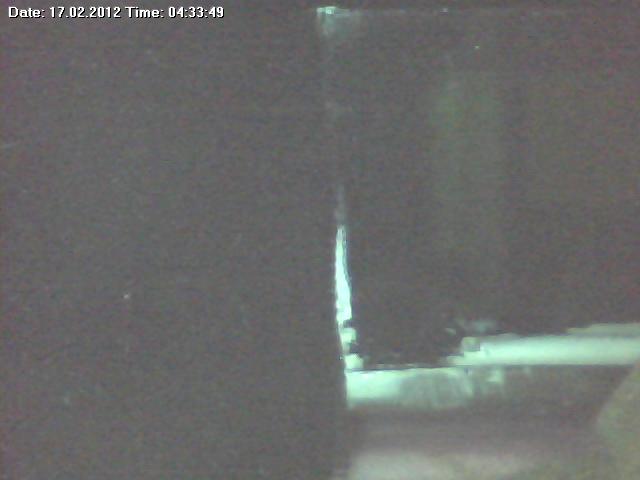

Supplement: Supplementary file 1 — Supplementary material [file mmc1.zip › Supplementary files/Supplementary Figure 1174.jpg]

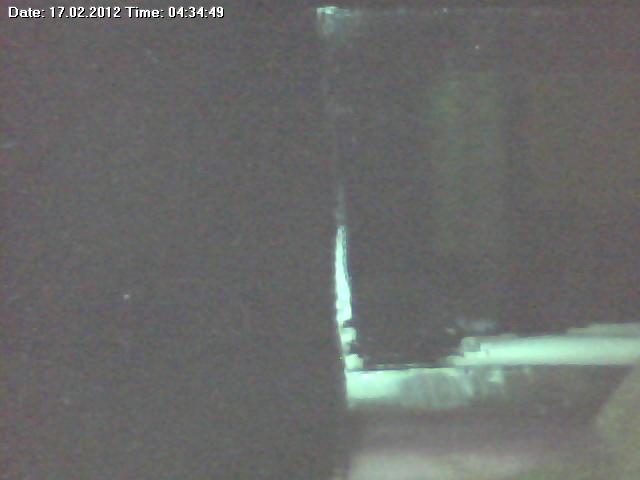

Supplement: Supplementary file 1 — Supplementary material [file mmc1.zip › Supplementary files/Supplementary Figure 1175.jpg]

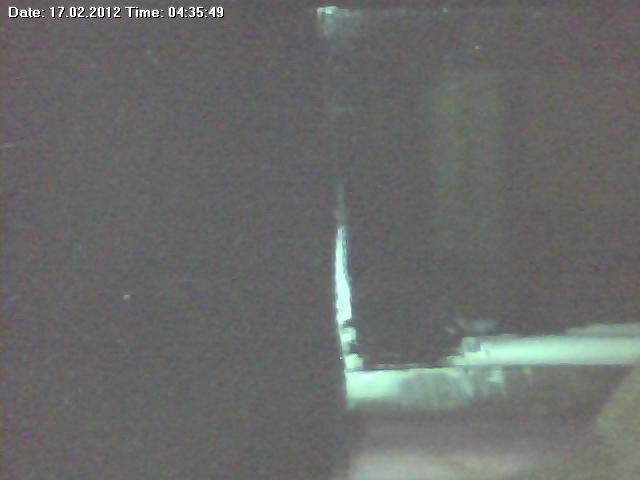

Supplement: Supplementary file 1 — Supplementary material [file mmc1.zip › Supplementary files/Supplementary Figure 1176.jpg]

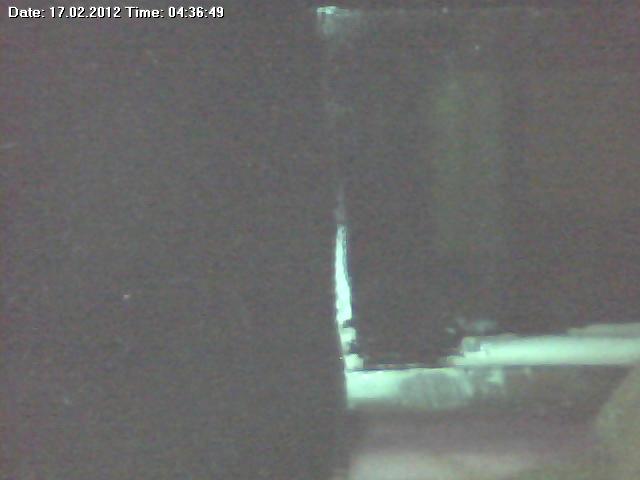

Supplement: Supplementary file 1 — Supplementary material [file mmc1.zip › Supplementary files/Supplementary Figure 1177.jpg]

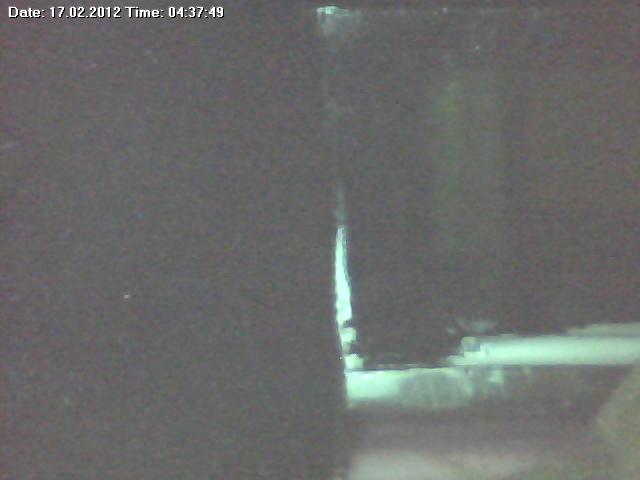

Supplement: Supplementary file 1 — Supplementary material [file mmc1.zip › Supplementary files/Supplementary Figure 1178.jpg]
