# Supplementary figures and images for: Experimental data showing the thermal behavior of a flat roof with phase change material (part 1 of 2)
Source: Data Brief. 2015 Oct 22;5:476–80. doi: 10.1016/j.dib.2015.09.019 (PMC4631864; doi:10.1016/j.dib.2015.09.019)

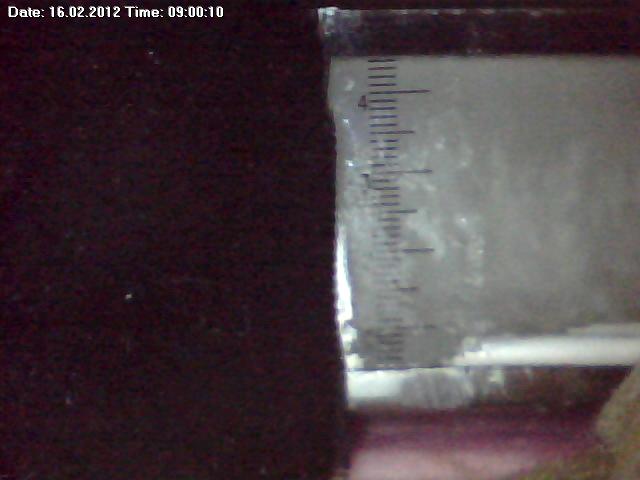

Supplement: Supplementary file 1 — Supplementary material [file mmc1.zip › Supplementary files/Supplementary Figure 1.jpg]

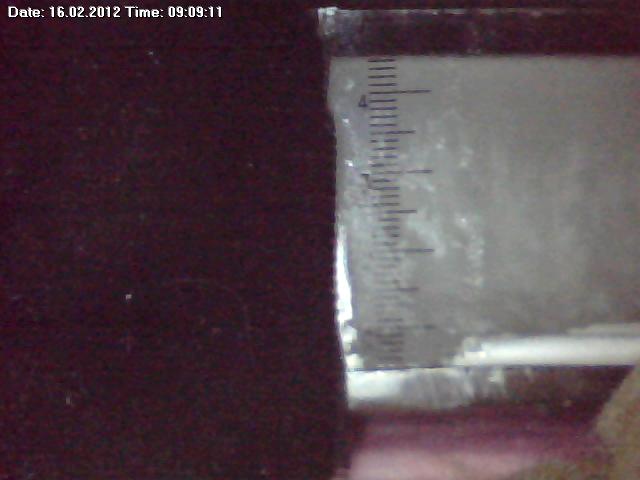

Supplement: Supplementary file 1 — Supplementary material [file mmc1.zip › Supplementary files/Supplementary Figure 10.jpg]

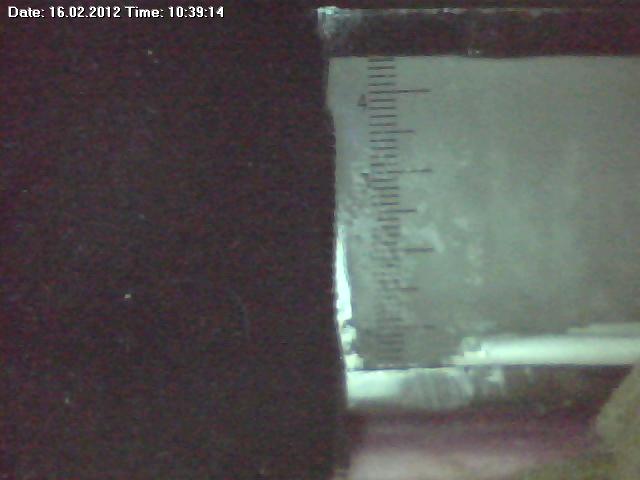

Supplement: Supplementary file 1 — Supplementary material [file mmc1.zip › Supplementary files/Supplementary Figure 100.jpg]

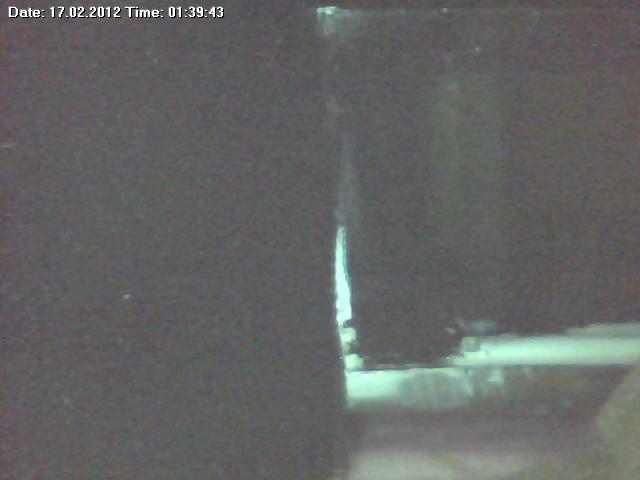

Supplement: Supplementary file 1 — Supplementary material [file mmc1.zip › Supplementary files/Supplementary Figure 1000.jpg]

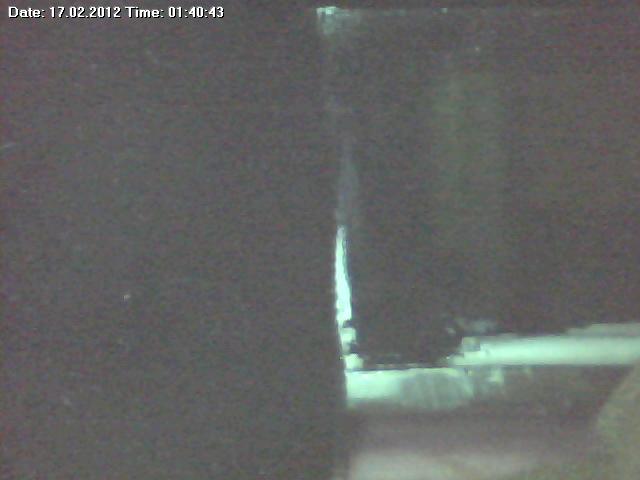

Supplement: Supplementary file 1 — Supplementary material [file mmc1.zip › Supplementary files/Supplementary Figure 1001.jpg]

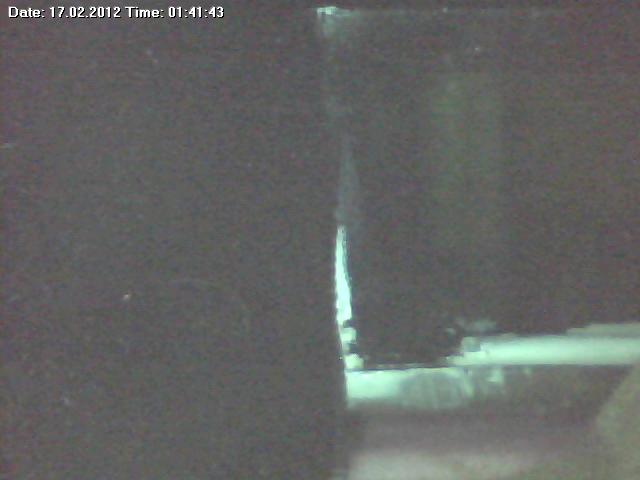

Supplement: Supplementary file 1 — Supplementary material [file mmc1.zip › Supplementary files/Supplementary Figure 1002.jpg]

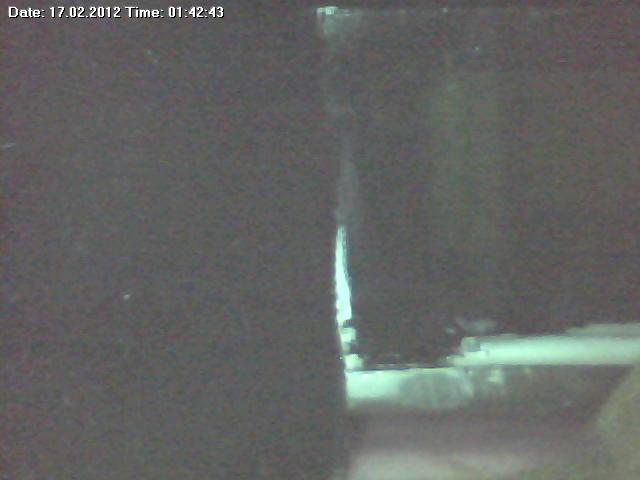

Supplement: Supplementary file 1 — Supplementary material [file mmc1.zip › Supplementary files/Supplementary Figure 1003.jpg]

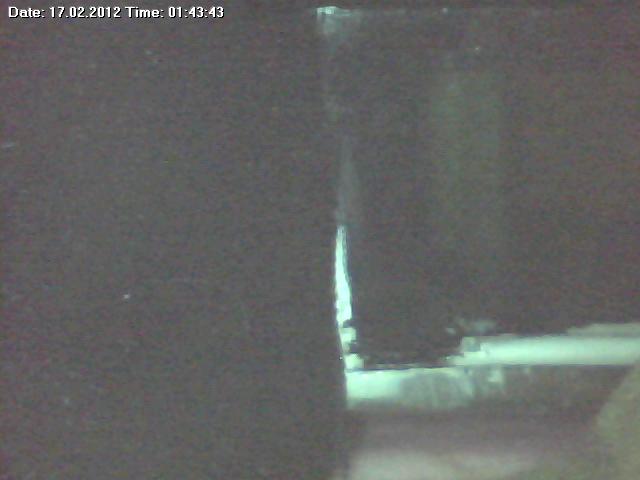

Supplement: Supplementary file 1 — Supplementary material [file mmc1.zip › Supplementary files/Supplementary Figure 1004.jpg]

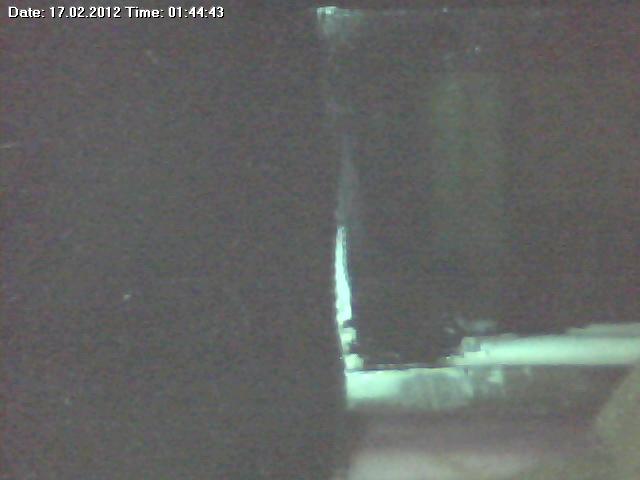

Supplement: Supplementary file 1 — Supplementary material [file mmc1.zip › Supplementary files/Supplementary Figure 1005.jpg]

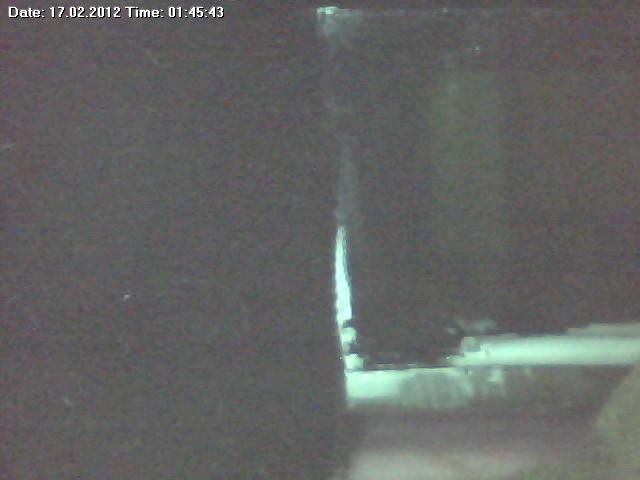

Supplement: Supplementary file 1 — Supplementary material [file mmc1.zip › Supplementary files/Supplementary Figure 1006.jpg]

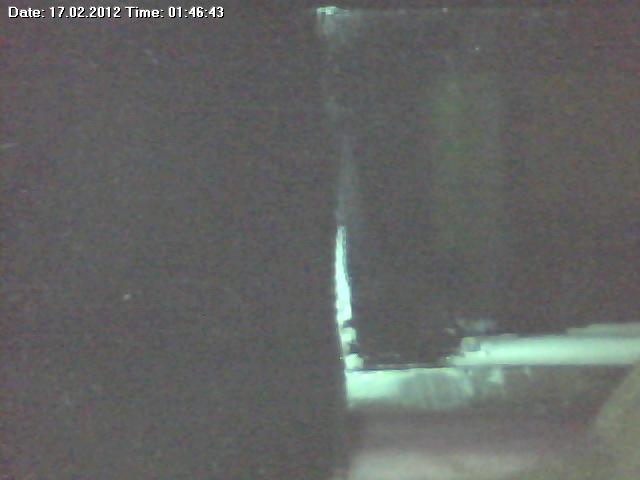

Supplement: Supplementary file 1 — Supplementary material [file mmc1.zip › Supplementary files/Supplementary Figure 1007.jpg]

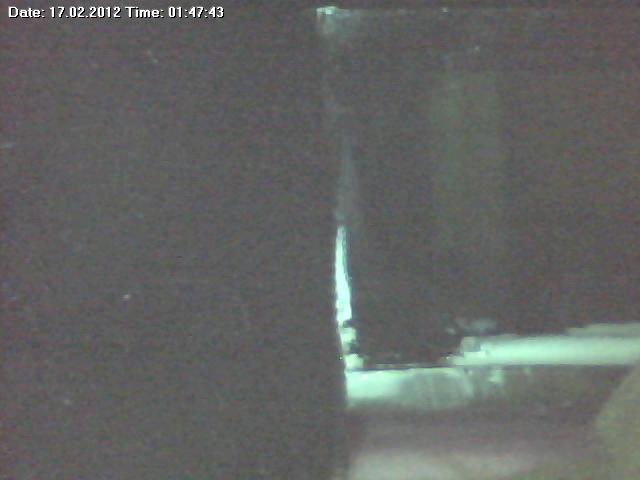

Supplement: Supplementary file 1 — Supplementary material [file mmc1.zip › Supplementary files/Supplementary Figure 1008.jpg]

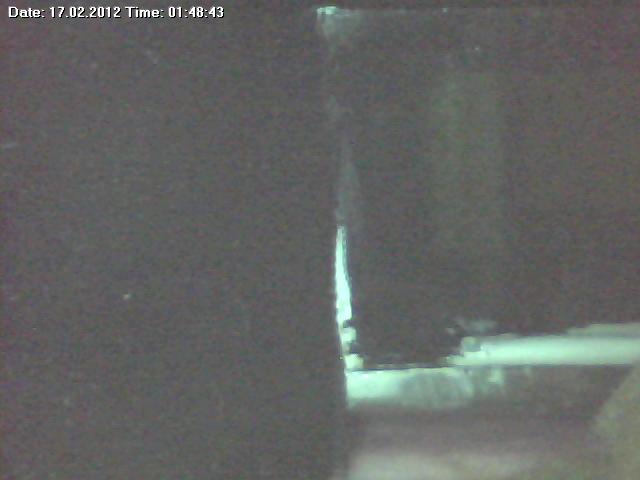

Supplement: Supplementary file 1 — Supplementary material [file mmc1.zip › Supplementary files/Supplementary Figure 1009.jpg]

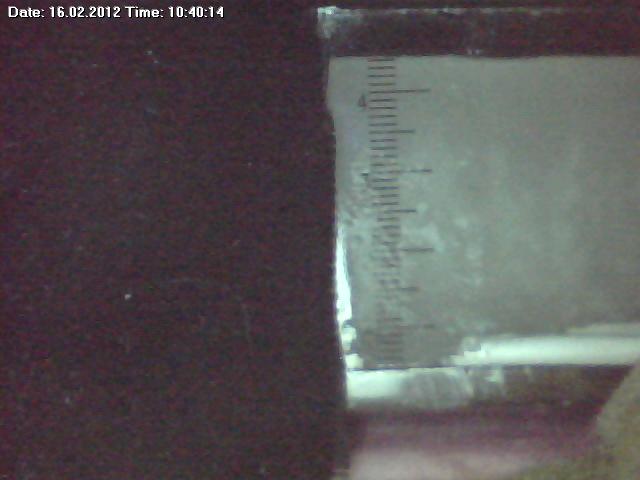

Supplement: Supplementary file 1 — Supplementary material [file mmc1.zip › Supplementary files/Supplementary Figure 101.jpg]

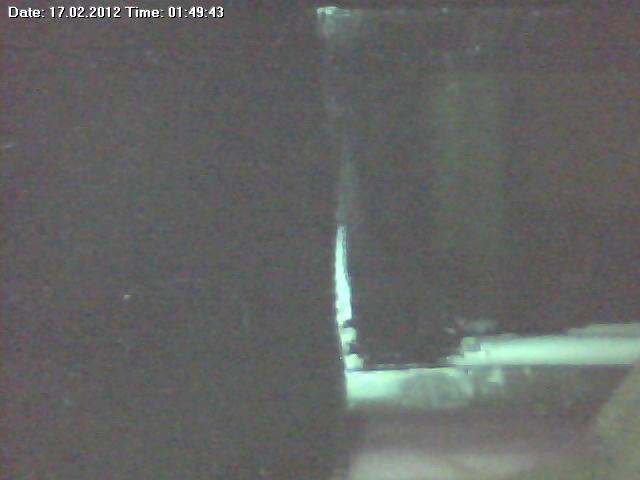

Supplement: Supplementary file 1 — Supplementary material [file mmc1.zip › Supplementary files/Supplementary Figure 1010.jpg]

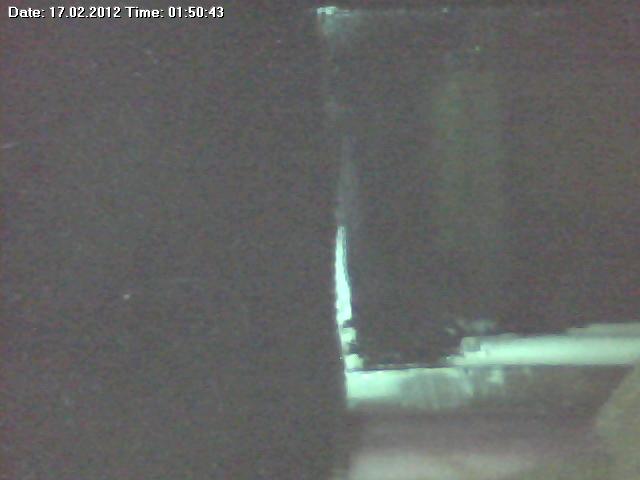

Supplement: Supplementary file 1 — Supplementary material [file mmc1.zip › Supplementary files/Supplementary Figure 1011.jpg]

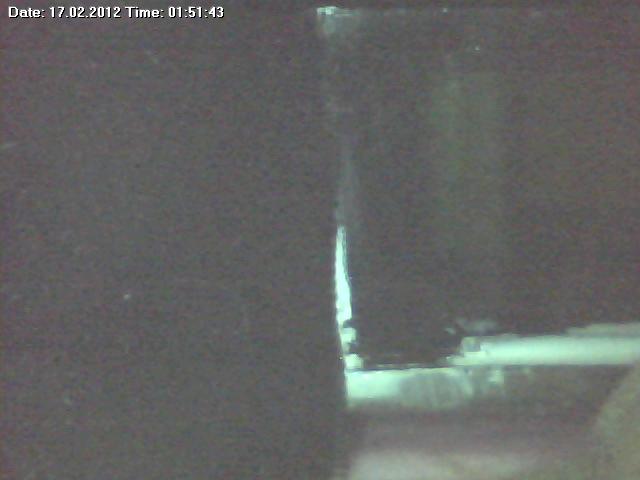

Supplement: Supplementary file 1 — Supplementary material [file mmc1.zip › Supplementary files/Supplementary Figure 1012.jpg]

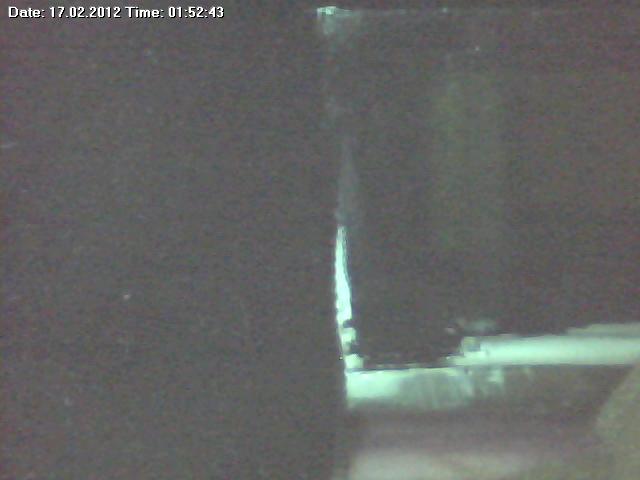

Supplement: Supplementary file 1 — Supplementary material [file mmc1.zip › Supplementary files/Supplementary Figure 1013.jpg]

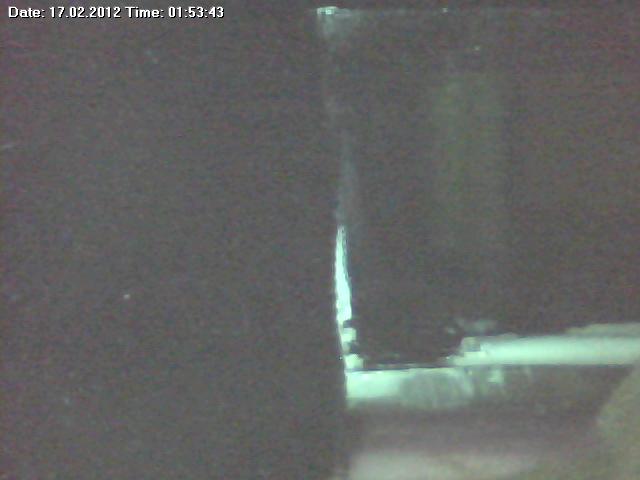

Supplement: Supplementary file 1 — Supplementary material [file mmc1.zip › Supplementary files/Supplementary Figure 1014.jpg]

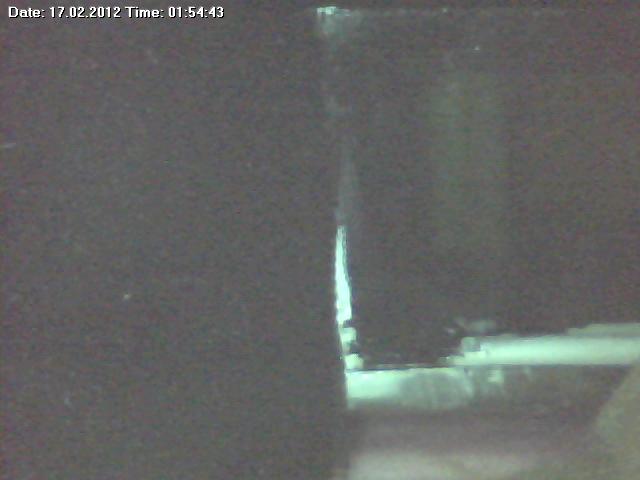

Supplement: Supplementary file 1 — Supplementary material [file mmc1.zip › Supplementary files/Supplementary Figure 1015.jpg]

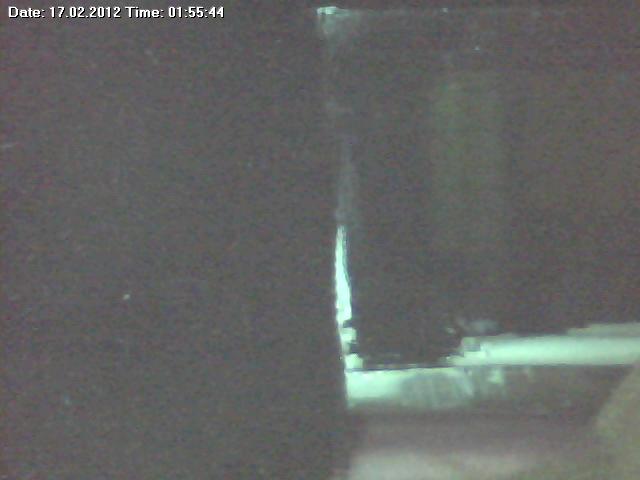

Supplement: Supplementary file 1 — Supplementary material [file mmc1.zip › Supplementary files/Supplementary Figure 1016.jpg]

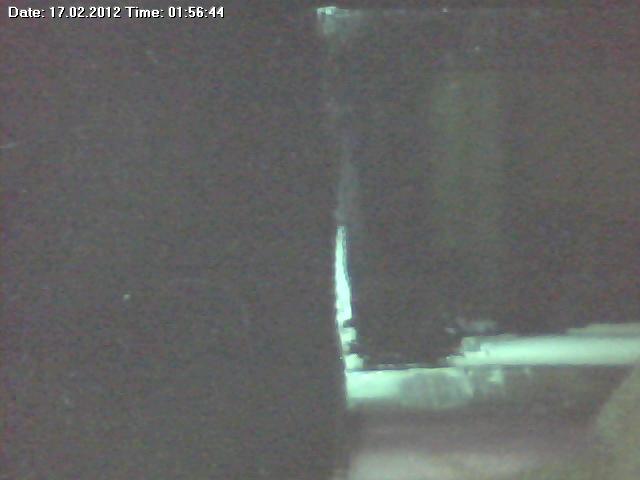

Supplement: Supplementary file 1 — Supplementary material [file mmc1.zip › Supplementary files/Supplementary Figure 1017.jpg]

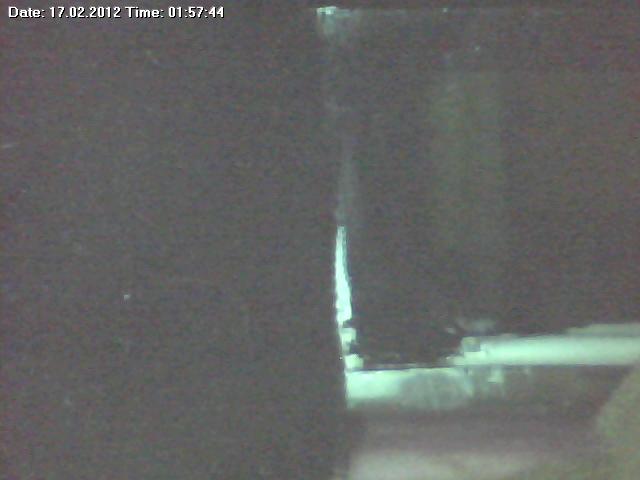

Supplement: Supplementary file 1 — Supplementary material [file mmc1.zip › Supplementary files/Supplementary Figure 1018.jpg]

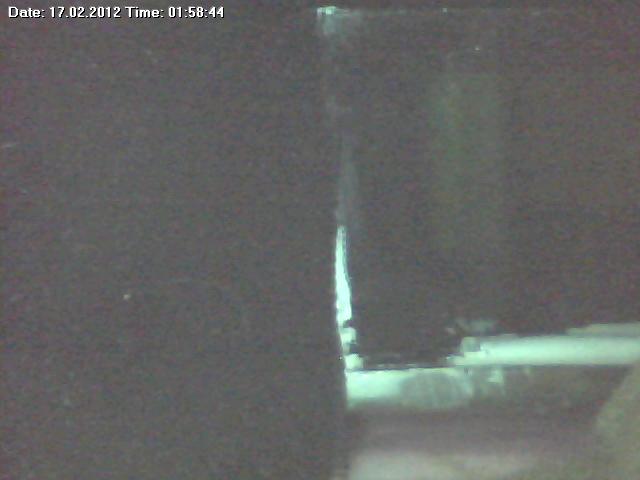

Supplement: Supplementary file 1 — Supplementary material [file mmc1.zip › Supplementary files/Supplementary Figure 1019.jpg]

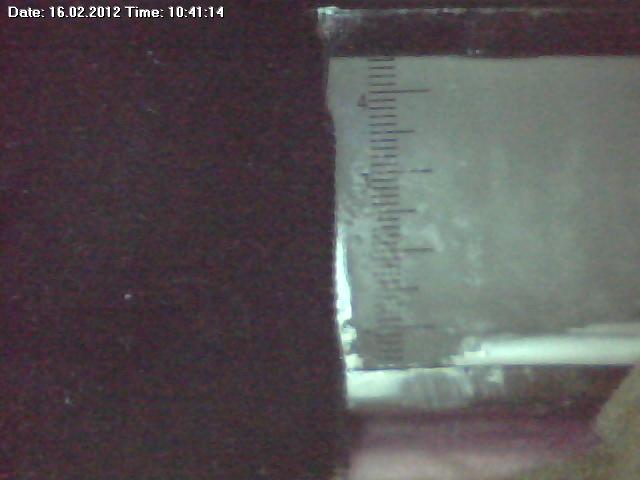

Supplement: Supplementary file 1 — Supplementary material [file mmc1.zip › Supplementary files/Supplementary Figure 102.jpg]

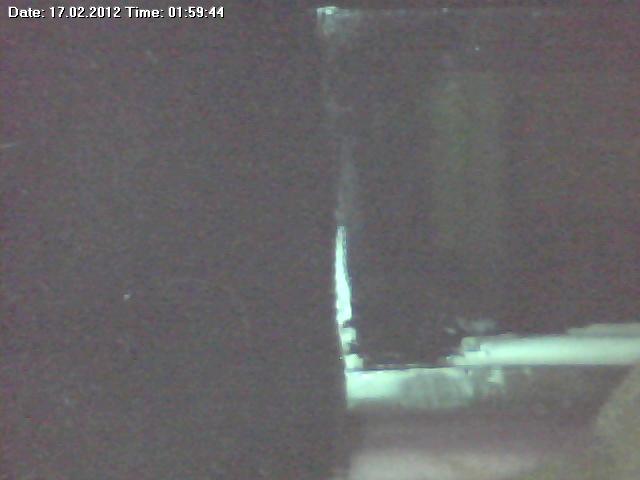

Supplement: Supplementary file 1 — Supplementary material [file mmc1.zip › Supplementary files/Supplementary Figure 1020.jpg]

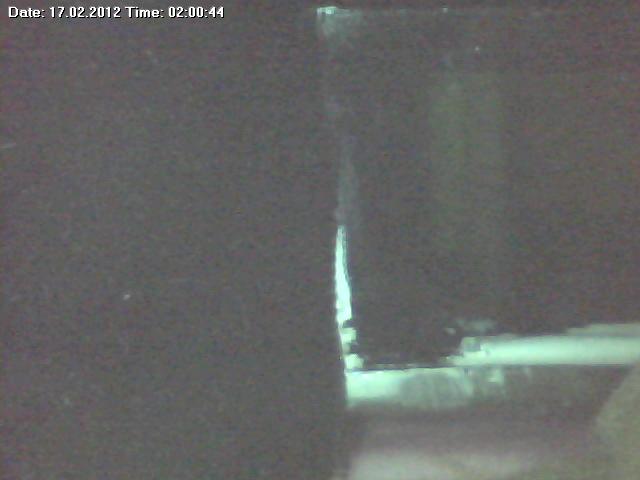

Supplement: Supplementary file 1 — Supplementary material [file mmc1.zip › Supplementary files/Supplementary Figure 1021.jpg]

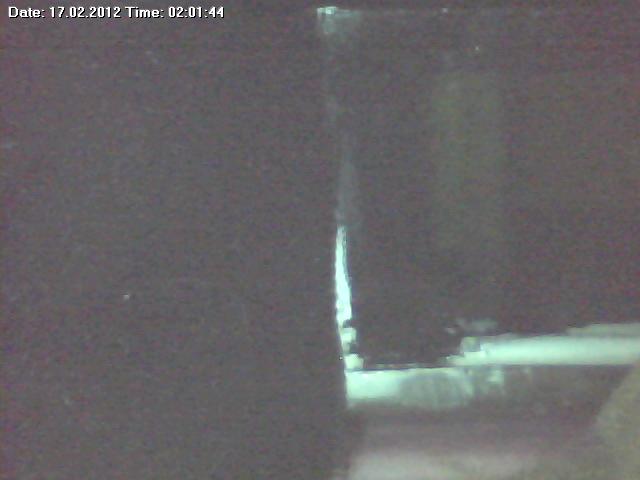

Supplement: Supplementary file 1 — Supplementary material [file mmc1.zip › Supplementary files/Supplementary Figure 1022.jpg]

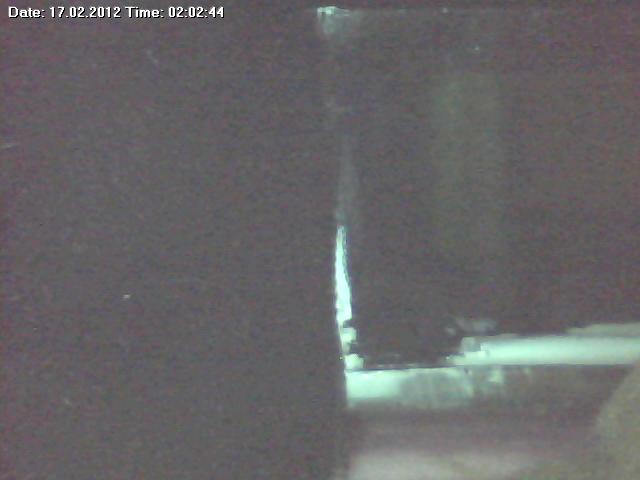

Supplement: Supplementary file 1 — Supplementary material [file mmc1.zip › Supplementary files/Supplementary Figure 1023.jpg]

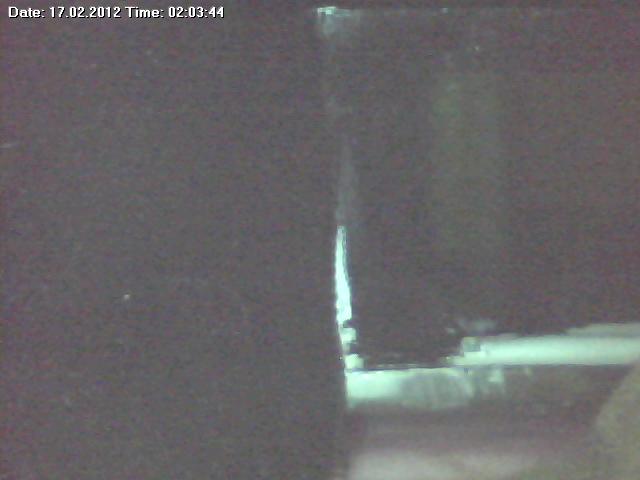

Supplement: Supplementary file 1 — Supplementary material [file mmc1.zip › Supplementary files/Supplementary Figure 1024.jpg]

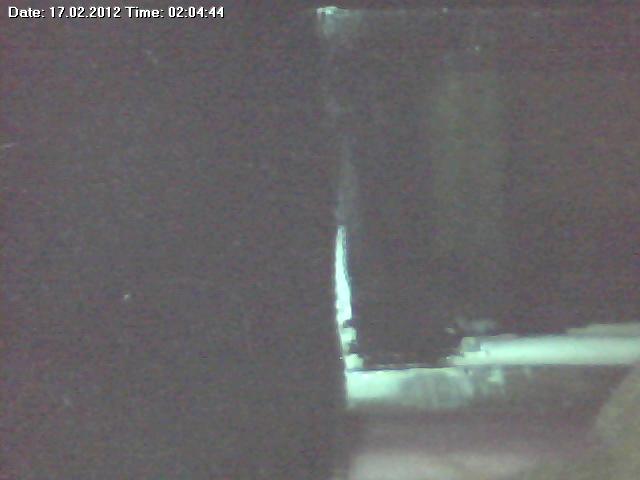

Supplement: Supplementary file 1 — Supplementary material [file mmc1.zip › Supplementary files/Supplementary Figure 1025.jpg]

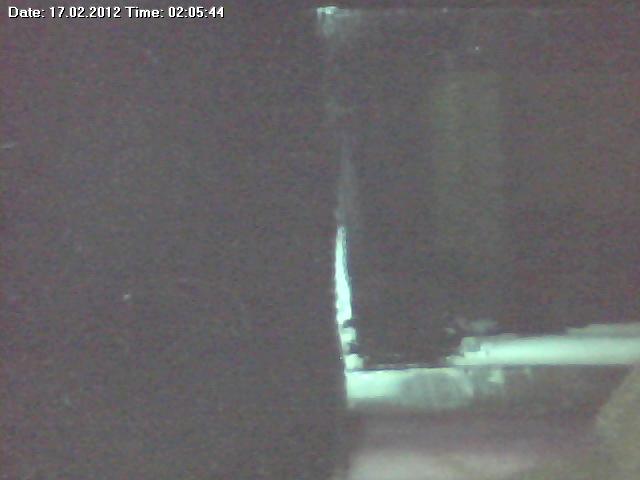

Supplement: Supplementary file 1 — Supplementary material [file mmc1.zip › Supplementary files/Supplementary Figure 1026.jpg]

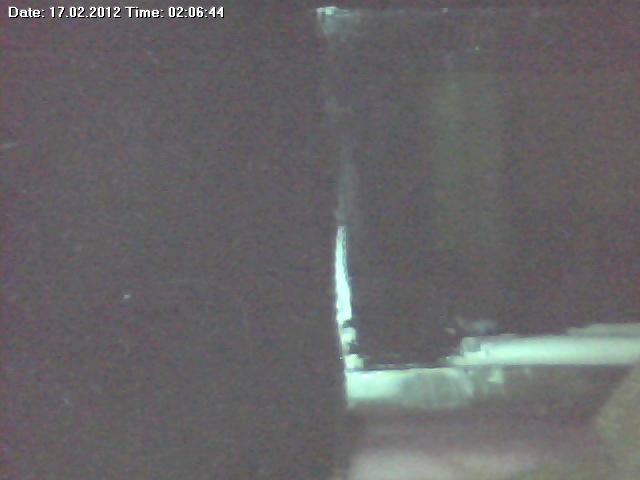

Supplement: Supplementary file 1 — Supplementary material [file mmc1.zip › Supplementary files/Supplementary Figure 1027.jpg]

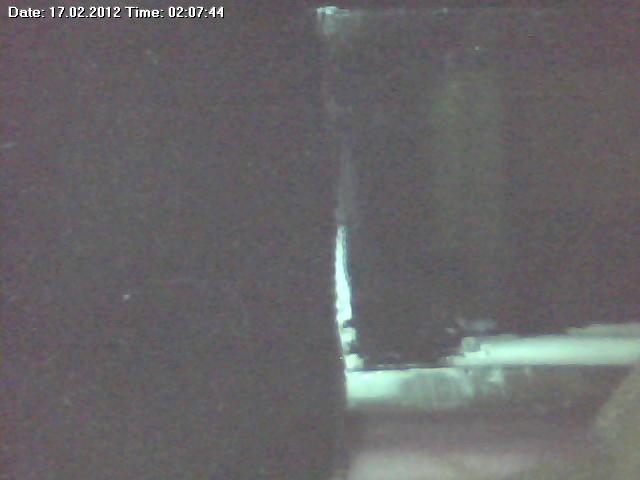

Supplement: Supplementary file 1 — Supplementary material [file mmc1.zip › Supplementary files/Supplementary Figure 1028.jpg]

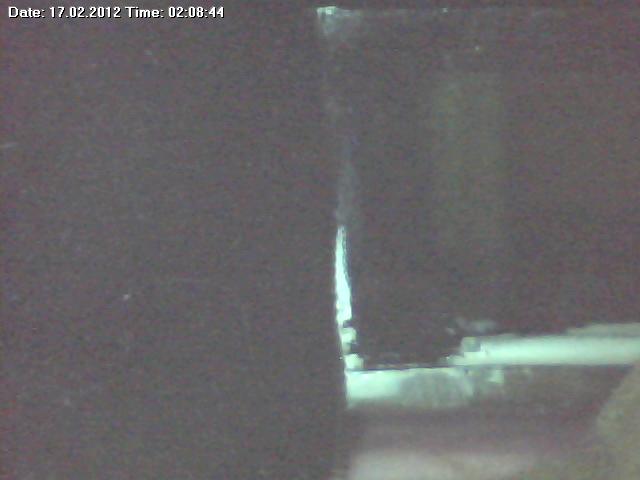

Supplement: Supplementary file 1 — Supplementary material [file mmc1.zip › Supplementary files/Supplementary Figure 1029.jpg]

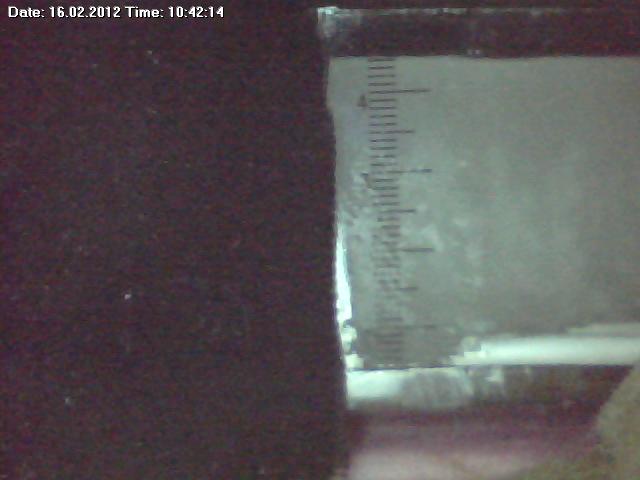

Supplement: Supplementary file 1 — Supplementary material [file mmc1.zip › Supplementary files/Supplementary Figure 103.jpg]

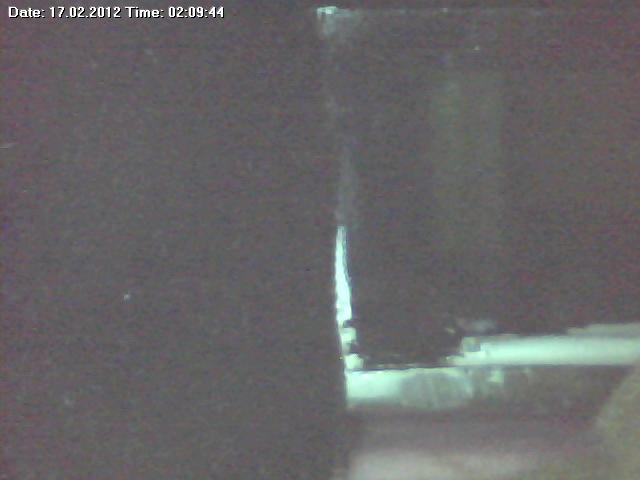

Supplement: Supplementary file 1 — Supplementary material [file mmc1.zip › Supplementary files/Supplementary Figure 1030.jpg]

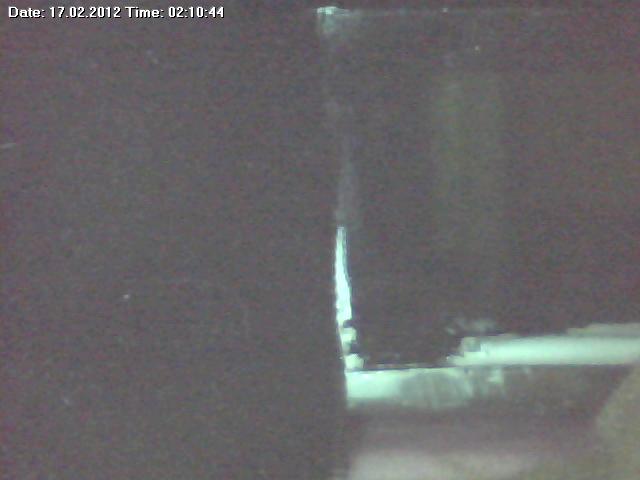

Supplement: Supplementary file 1 — Supplementary material [file mmc1.zip › Supplementary files/Supplementary Figure 1031.jpg]

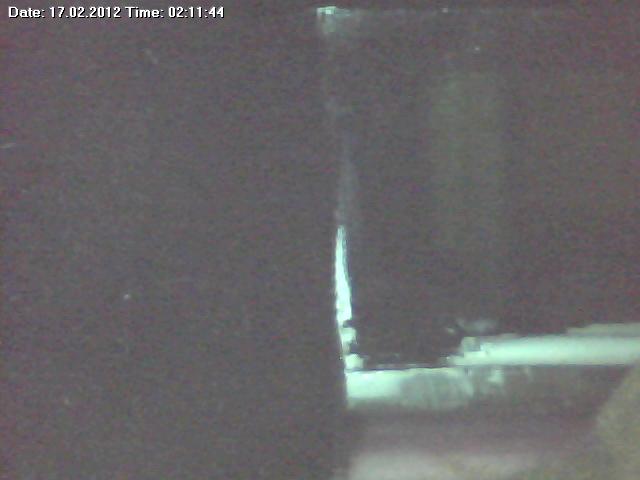

Supplement: Supplementary file 1 — Supplementary material [file mmc1.zip › Supplementary files/Supplementary Figure 1032.jpg]

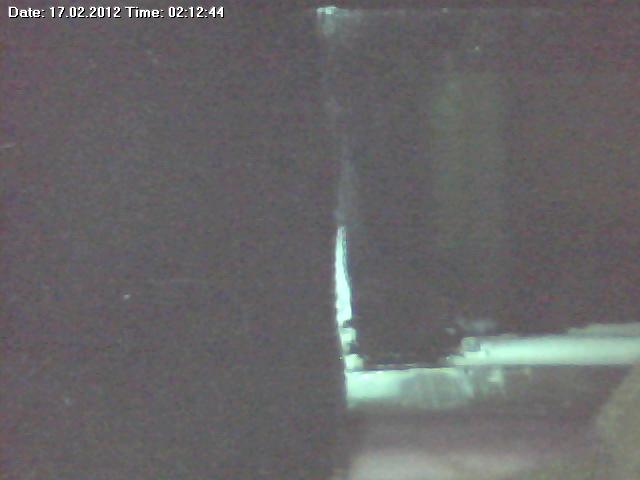

Supplement: Supplementary file 1 — Supplementary material [file mmc1.zip › Supplementary files/Supplementary Figure 1033.jpg]

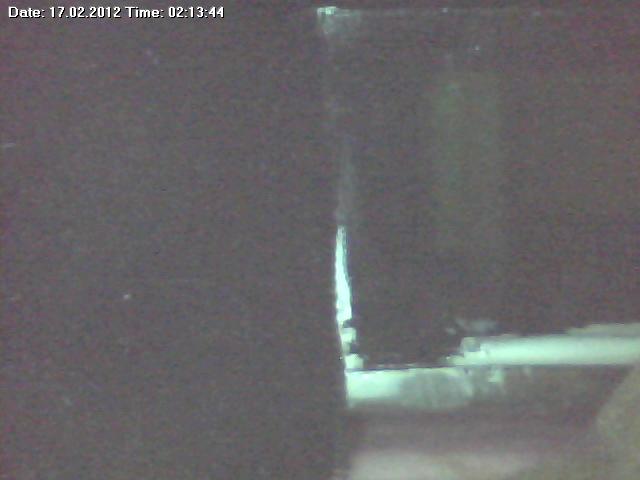

Supplement: Supplementary file 1 — Supplementary material [file mmc1.zip › Supplementary files/Supplementary Figure 1034.jpg]

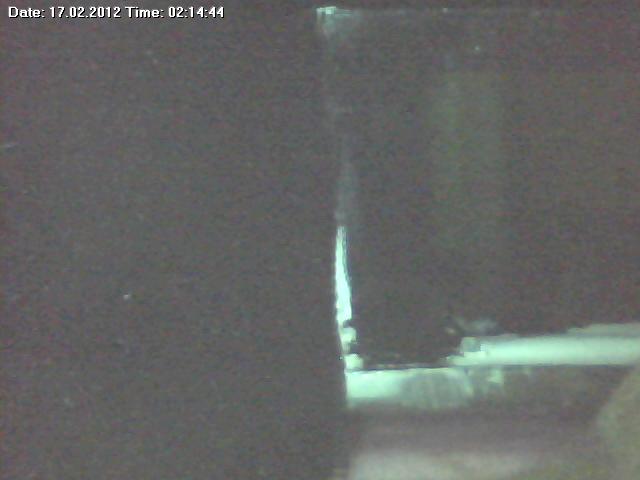

Supplement: Supplementary file 1 — Supplementary material [file mmc1.zip › Supplementary files/Supplementary Figure 1035.jpg]

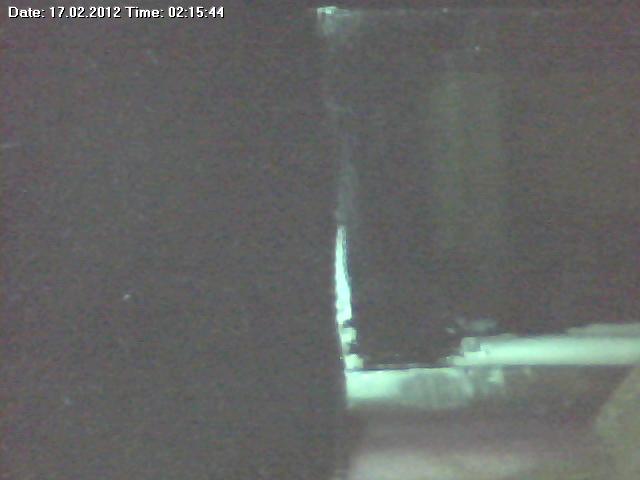

Supplement: Supplementary file 1 — Supplementary material [file mmc1.zip › Supplementary files/Supplementary Figure 1036.jpg]

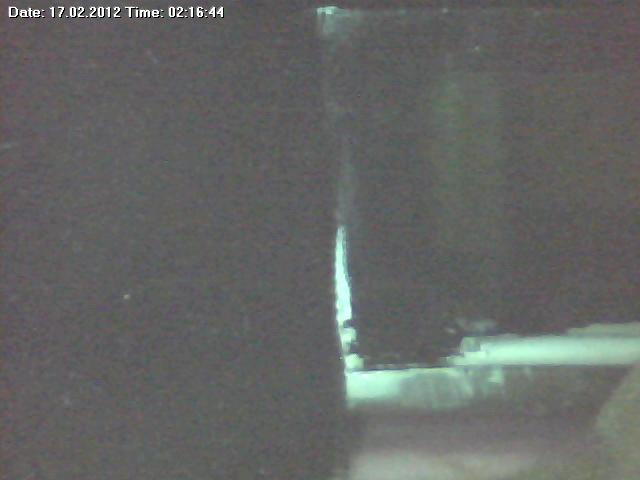

Supplement: Supplementary file 1 — Supplementary material [file mmc1.zip › Supplementary files/Supplementary Figure 1037.jpg]

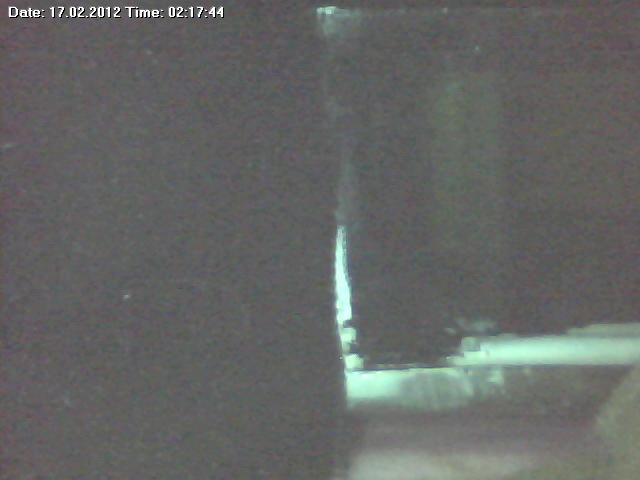

Supplement: Supplementary file 1 — Supplementary material [file mmc1.zip › Supplementary files/Supplementary Figure 1038.jpg]

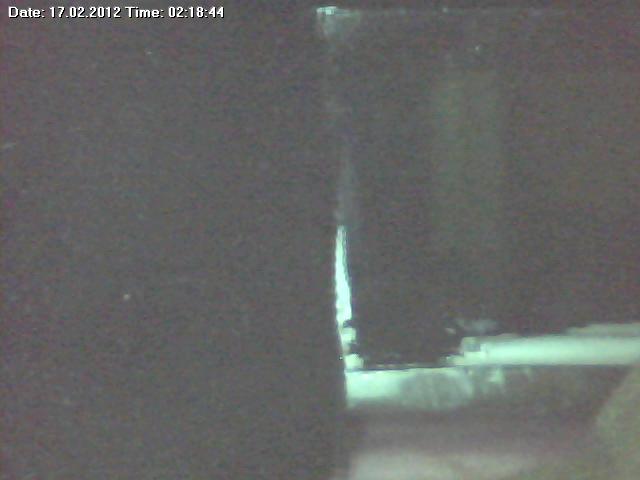

Supplement: Supplementary file 1 — Supplementary material [file mmc1.zip › Supplementary files/Supplementary Figure 1039.jpg]

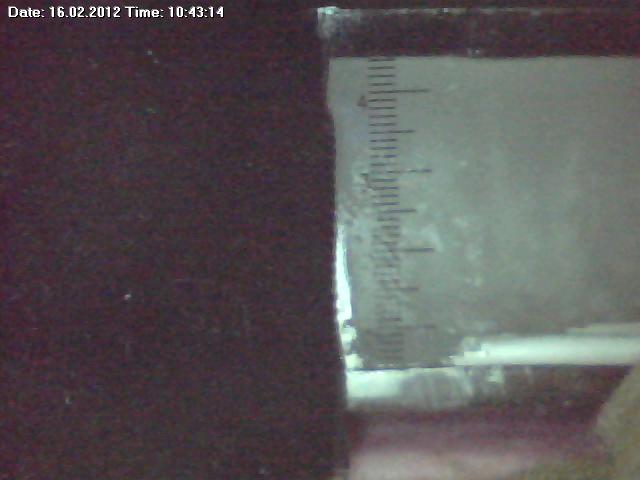

Supplement: Supplementary file 1 — Supplementary material [file mmc1.zip › Supplementary files/Supplementary Figure 104.jpg]

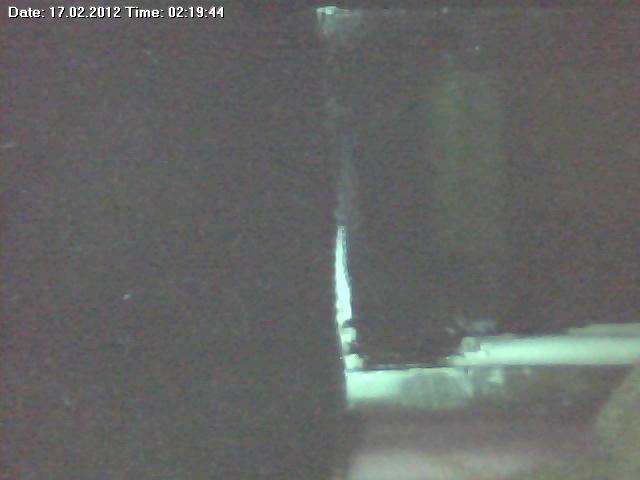

Supplement: Supplementary file 1 — Supplementary material [file mmc1.zip › Supplementary files/Supplementary Figure 1040.jpg]

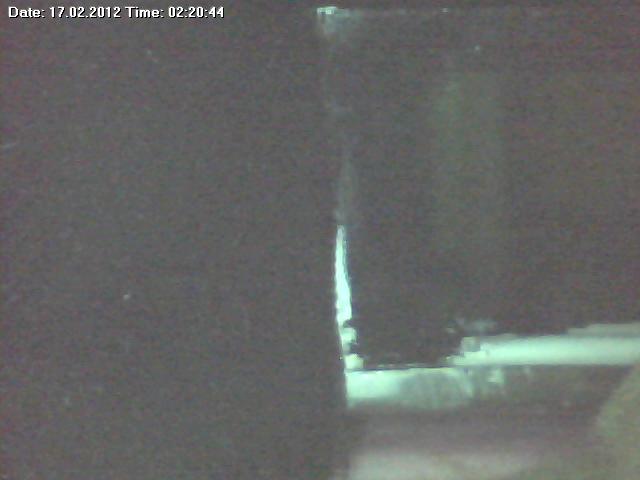

Supplement: Supplementary file 1 — Supplementary material [file mmc1.zip › Supplementary files/Supplementary Figure 1041.jpg]

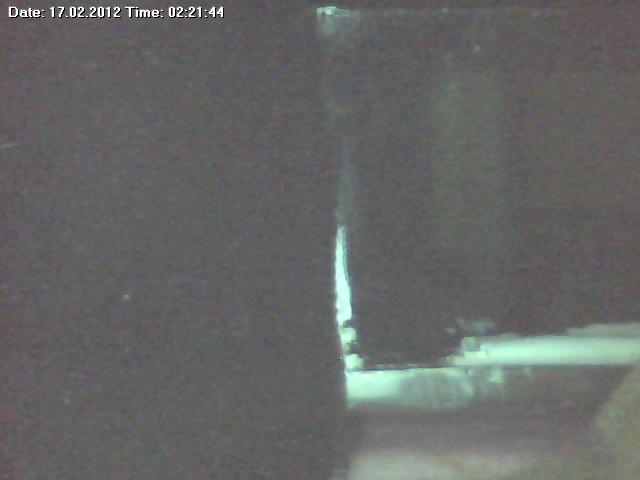

Supplement: Supplementary file 1 — Supplementary material [file mmc1.zip › Supplementary files/Supplementary Figure 1042.jpg]

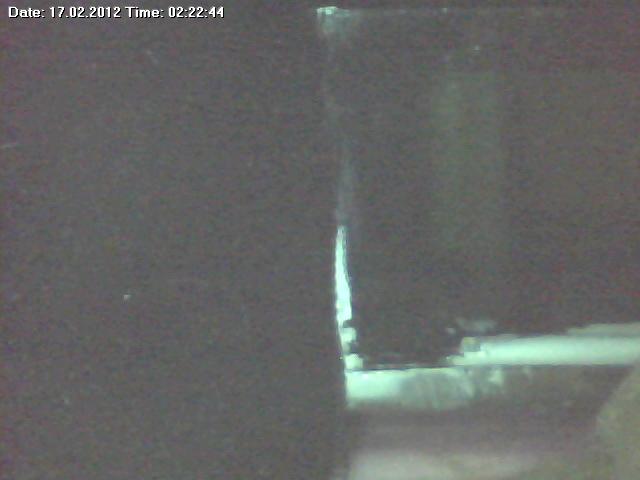

Supplement: Supplementary file 1 — Supplementary material [file mmc1.zip › Supplementary files/Supplementary Figure 1043.jpg]

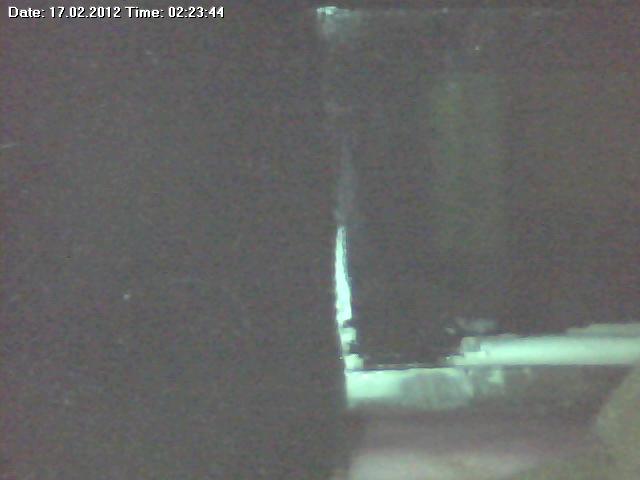

Supplement: Supplementary file 1 — Supplementary material [file mmc1.zip › Supplementary files/Supplementary Figure 1044.jpg]

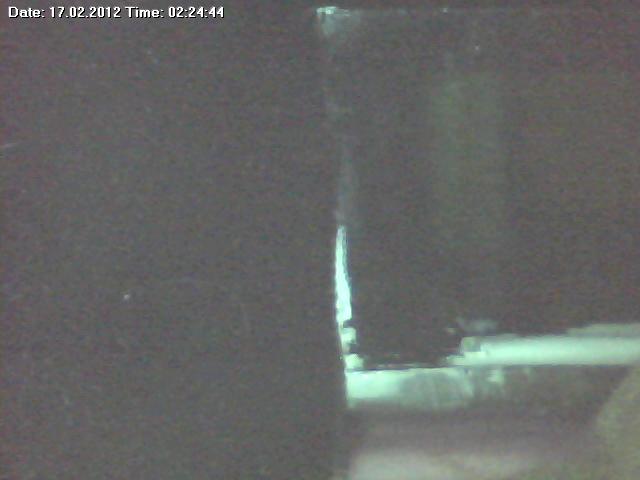

Supplement: Supplementary file 1 — Supplementary material [file mmc1.zip › Supplementary files/Supplementary Figure 1045.jpg]

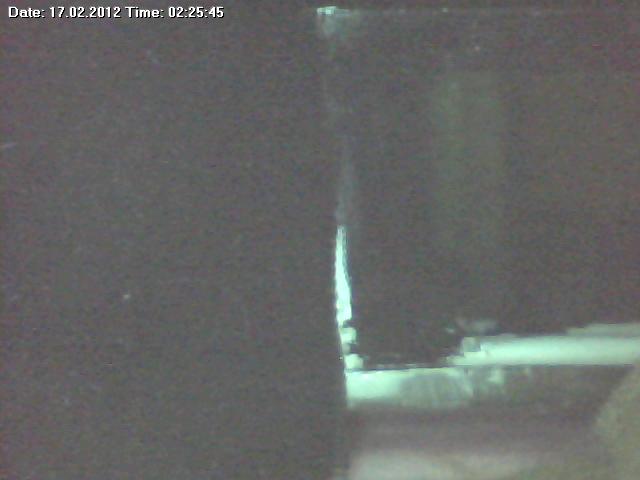

Supplement: Supplementary file 1 — Supplementary material [file mmc1.zip › Supplementary files/Supplementary Figure 1046.jpg]

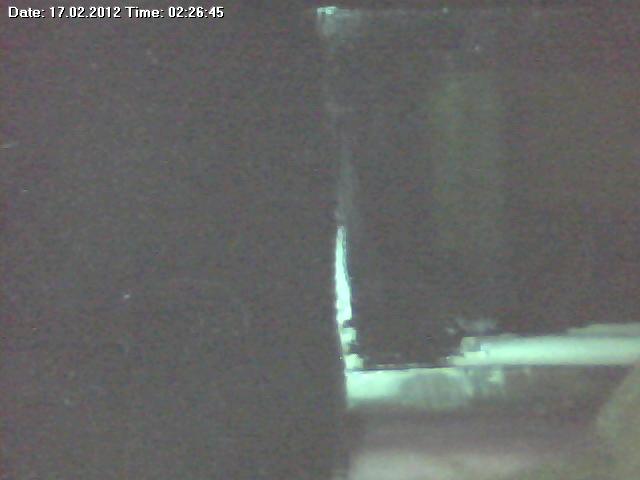

Supplement: Supplementary file 1 — Supplementary material [file mmc1.zip › Supplementary files/Supplementary Figure 1047.jpg]

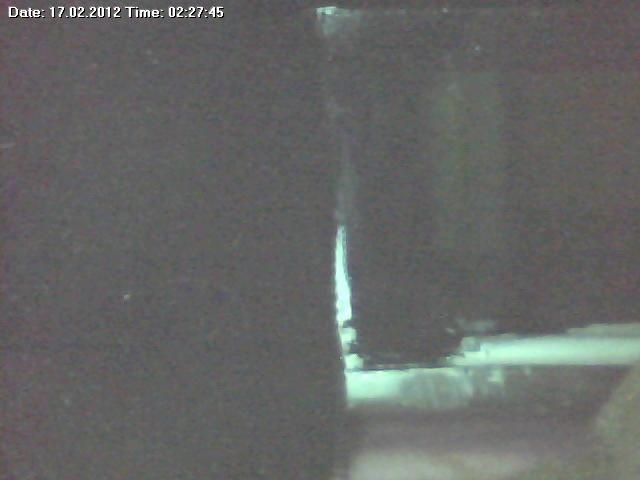

Supplement: Supplementary file 1 — Supplementary material [file mmc1.zip › Supplementary files/Supplementary Figure 1048.jpg]

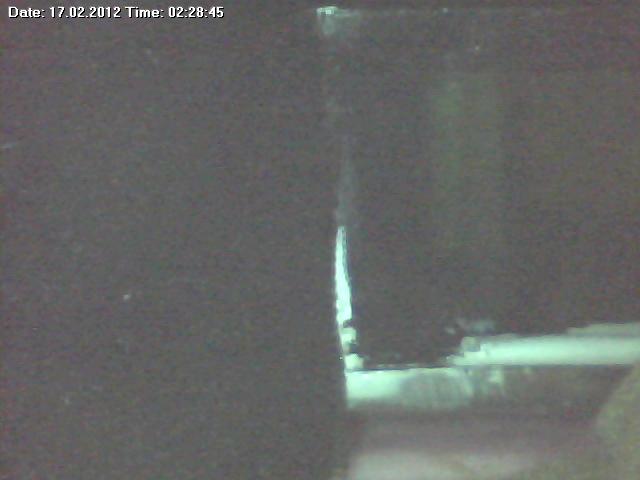

Supplement: Supplementary file 1 — Supplementary material [file mmc1.zip › Supplementary files/Supplementary Figure 1049.jpg]

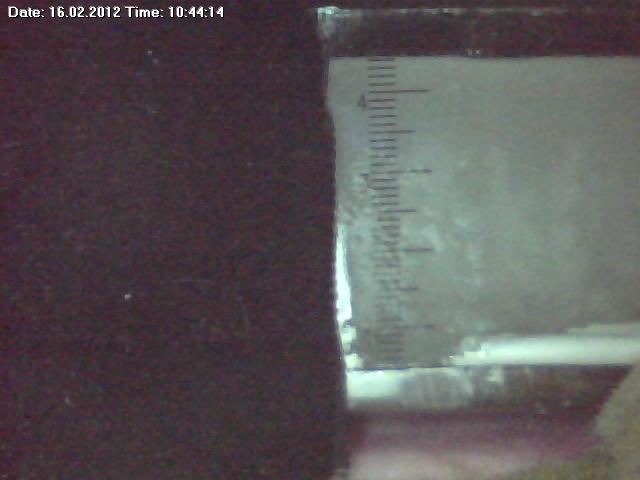

Supplement: Supplementary file 1 — Supplementary material [file mmc1.zip › Supplementary files/Supplementary Figure 105.jpg]

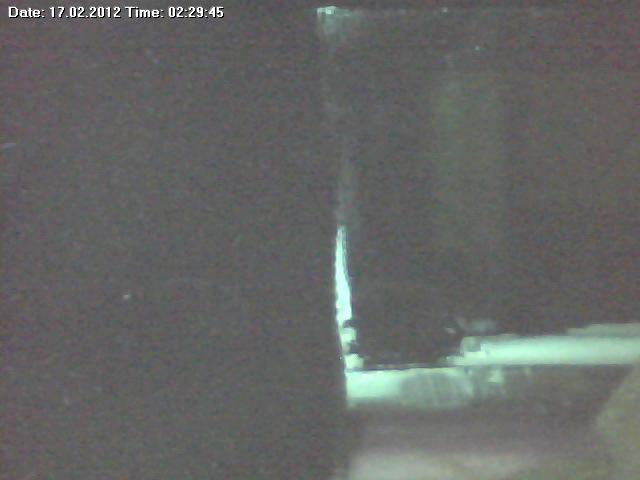

Supplement: Supplementary file 1 — Supplementary material [file mmc1.zip › Supplementary files/Supplementary Figure 1050.jpg]

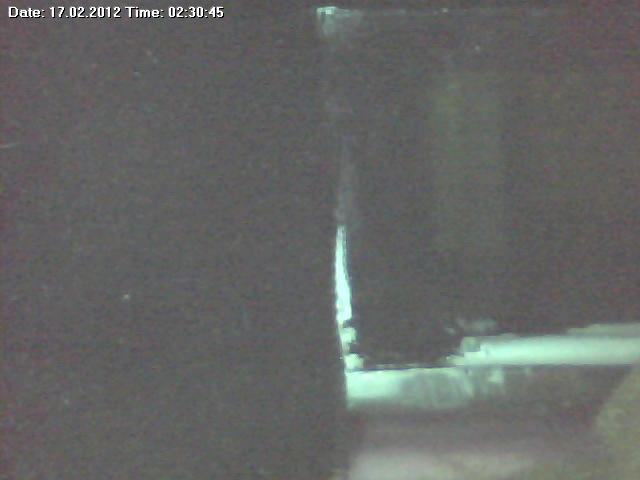

Supplement: Supplementary file 1 — Supplementary material [file mmc1.zip › Supplementary files/Supplementary Figure 1051.jpg]

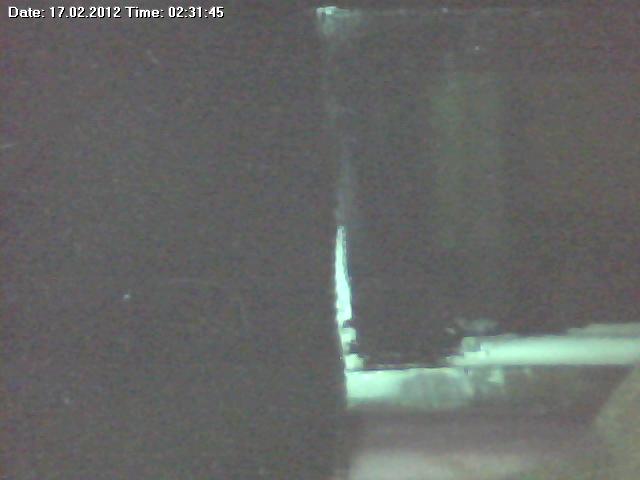

Supplement: Supplementary file 1 — Supplementary material [file mmc1.zip › Supplementary files/Supplementary Figure 1052.jpg]

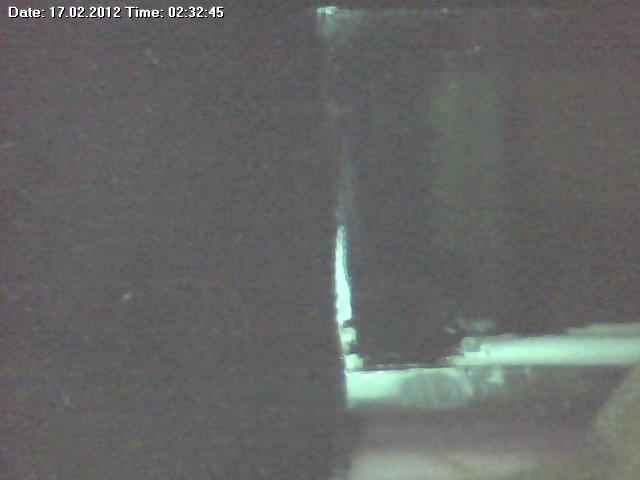

Supplement: Supplementary file 1 — Supplementary material [file mmc1.zip › Supplementary files/Supplementary Figure 1053.jpg]

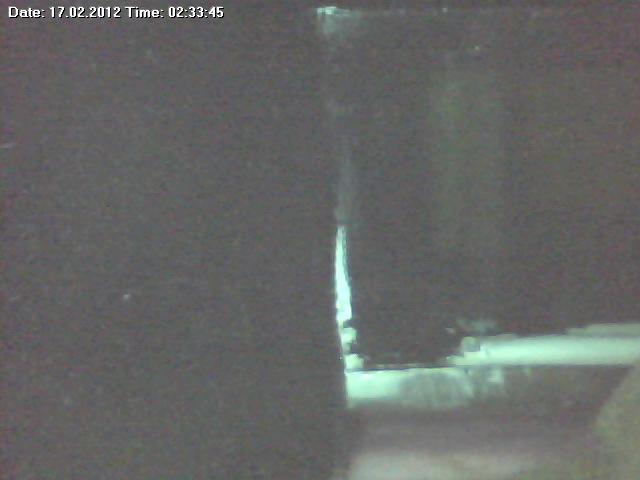

Supplement: Supplementary file 1 — Supplementary material [file mmc1.zip › Supplementary files/Supplementary Figure 1054.jpg]

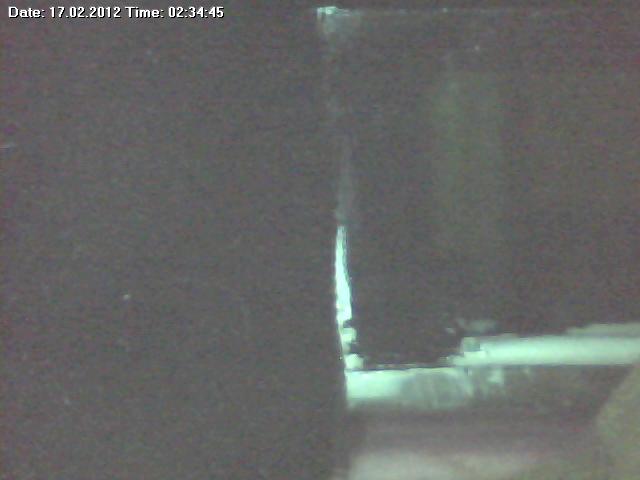

Supplement: Supplementary file 1 — Supplementary material [file mmc1.zip › Supplementary files/Supplementary Figure 1055.jpg]

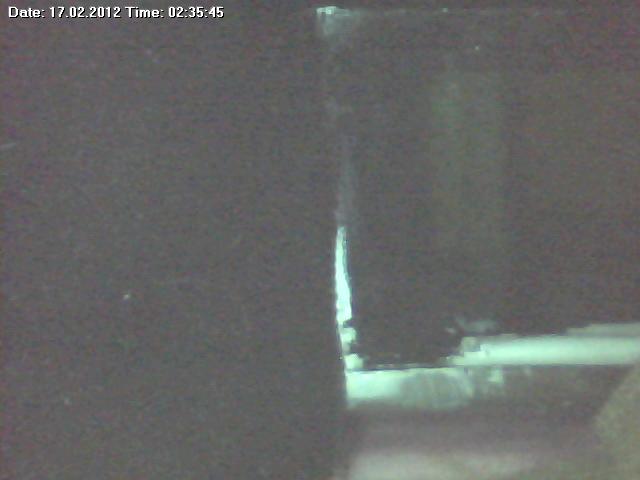

Supplement: Supplementary file 1 — Supplementary material [file mmc1.zip › Supplementary files/Supplementary Figure 1056.jpg]

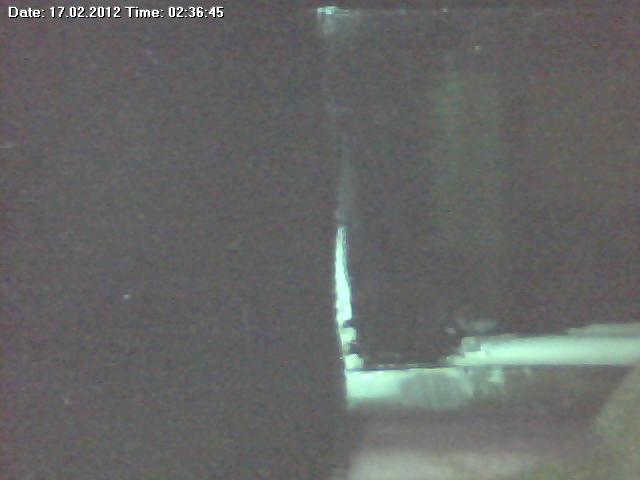

Supplement: Supplementary file 1 — Supplementary material [file mmc1.zip › Supplementary files/Supplementary Figure 1057.jpg]

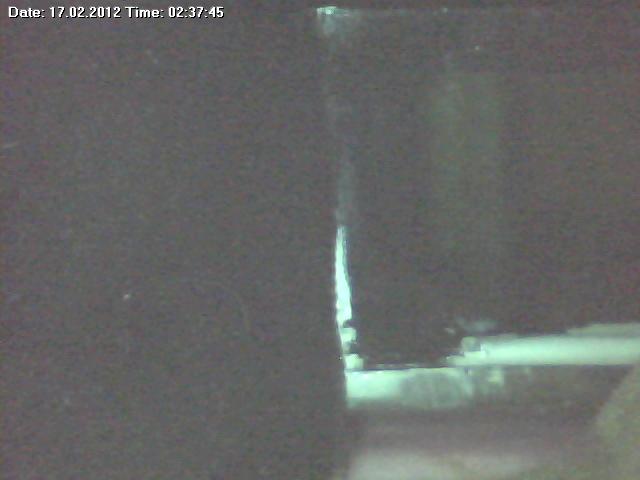

Supplement: Supplementary file 1 — Supplementary material [file mmc1.zip › Supplementary files/Supplementary Figure 1058.jpg]

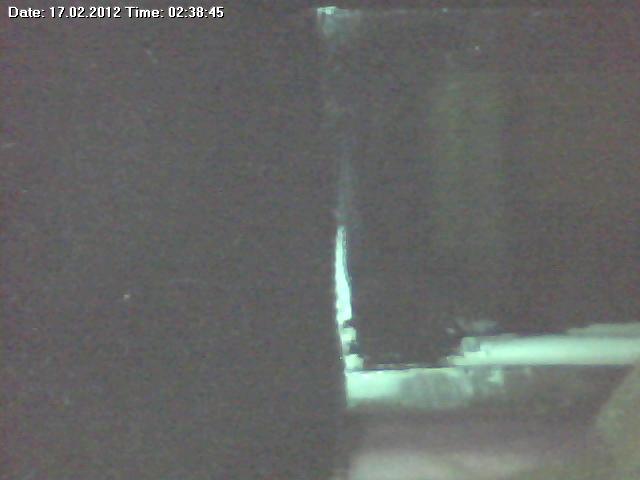

Supplement: Supplementary file 1 — Supplementary material [file mmc1.zip › Supplementary files/Supplementary Figure 1059.jpg]

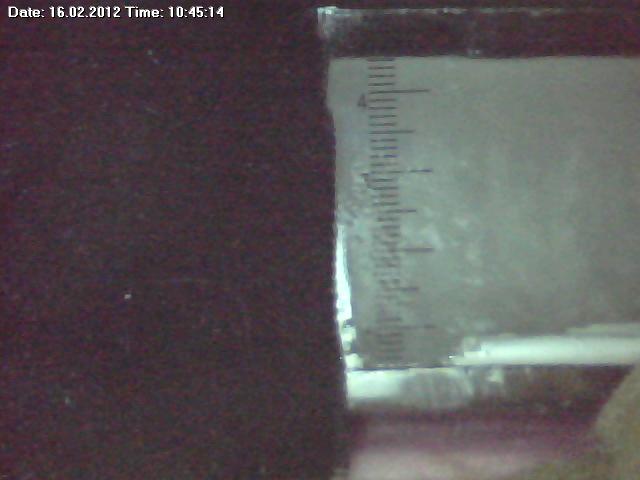

Supplement: Supplementary file 1 — Supplementary material [file mmc1.zip › Supplementary files/Supplementary Figure 106.jpg]

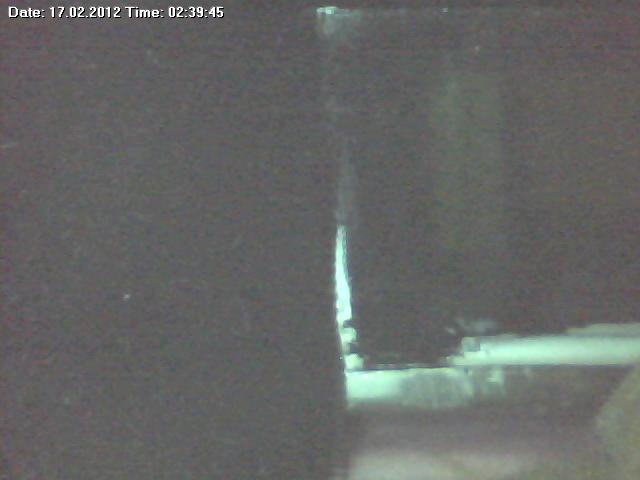

Supplement: Supplementary file 1 — Supplementary material [file mmc1.zip › Supplementary files/Supplementary Figure 1060.jpg]

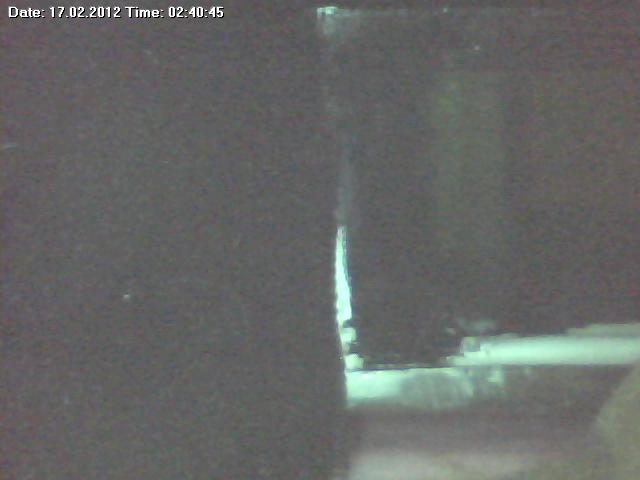

Supplement: Supplementary file 1 — Supplementary material [file mmc1.zip › Supplementary files/Supplementary Figure 1061.jpg]

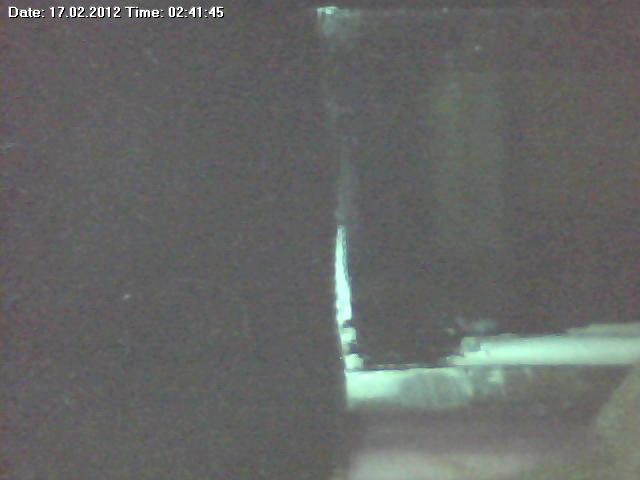

Supplement: Supplementary file 1 — Supplementary material [file mmc1.zip › Supplementary files/Supplementary Figure 1062.jpg]

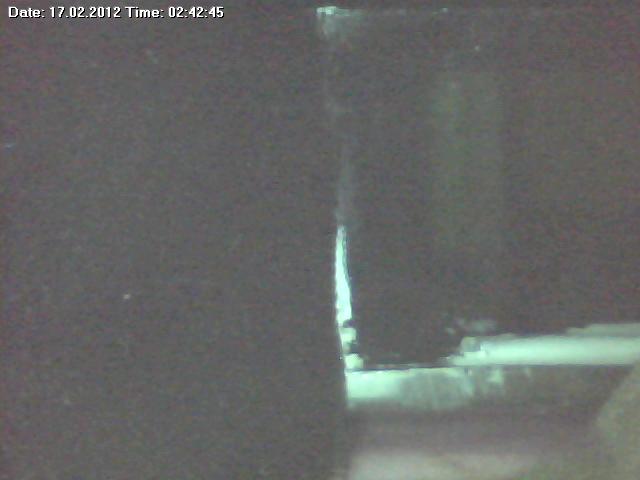

Supplement: Supplementary file 1 — Supplementary material [file mmc1.zip › Supplementary files/Supplementary Figure 1063.jpg]

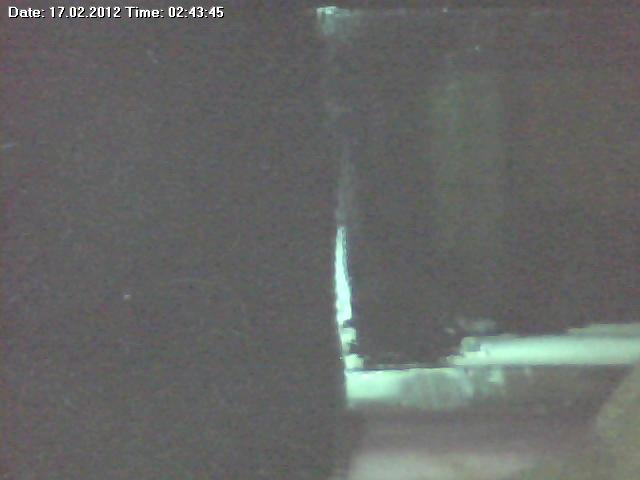

Supplement: Supplementary file 1 — Supplementary material [file mmc1.zip › Supplementary files/Supplementary Figure 1064.jpg]

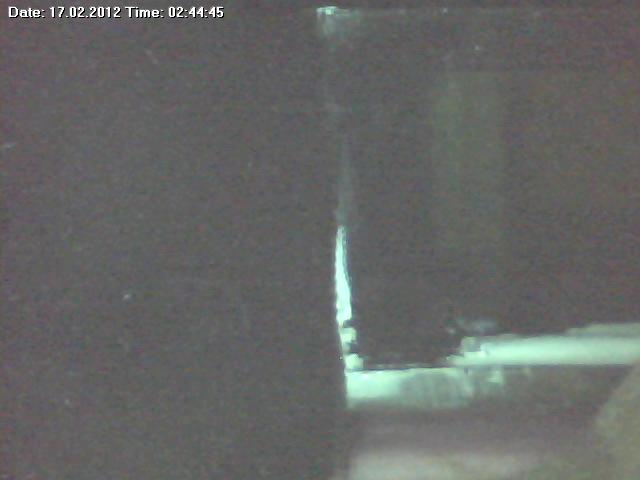

Supplement: Supplementary file 1 — Supplementary material [file mmc1.zip › Supplementary files/Supplementary Figure 1065.jpg]

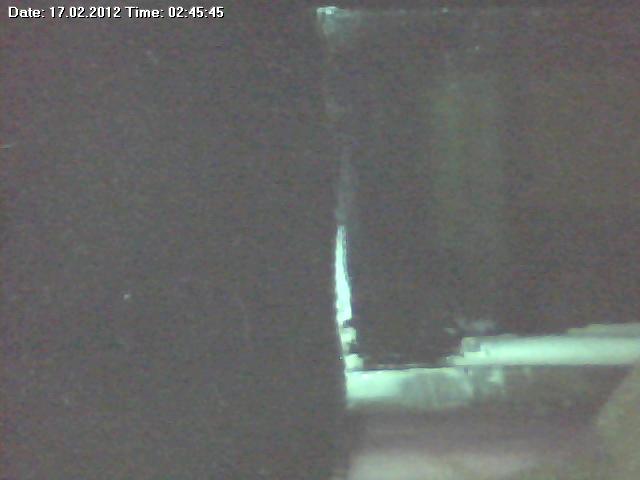

Supplement: Supplementary file 1 — Supplementary material [file mmc1.zip › Supplementary files/Supplementary Figure 1066.jpg]

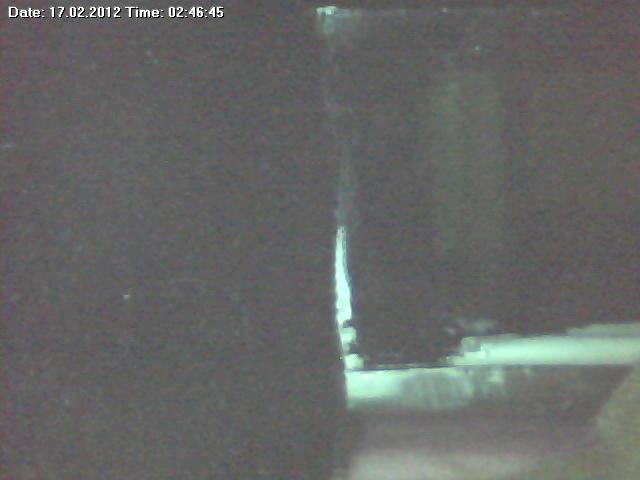

Supplement: Supplementary file 1 — Supplementary material [file mmc1.zip › Supplementary files/Supplementary Figure 1067.jpg]

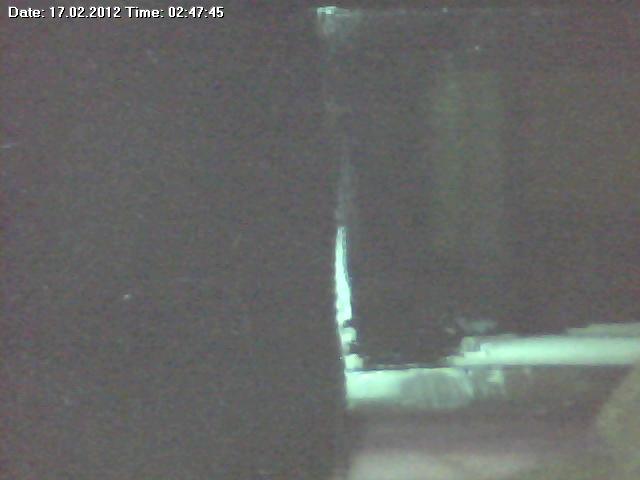

Supplement: Supplementary file 1 — Supplementary material [file mmc1.zip › Supplementary files/Supplementary Figure 1068.jpg]

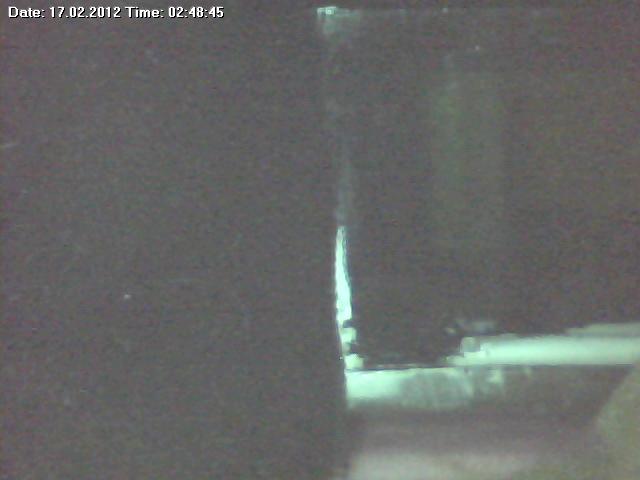

Supplement: Supplementary file 1 — Supplementary material [file mmc1.zip › Supplementary files/Supplementary Figure 1069.jpg]

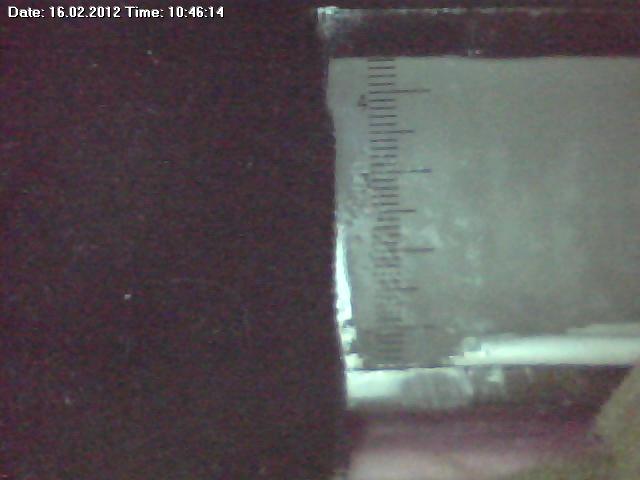

Supplement: Supplementary file 1 — Supplementary material [file mmc1.zip › Supplementary files/Supplementary Figure 107.jpg]

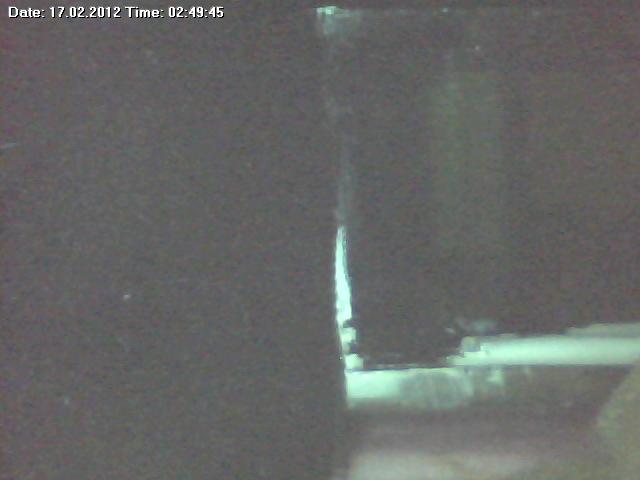

Supplement: Supplementary file 1 — Supplementary material [file mmc1.zip › Supplementary files/Supplementary Figure 1070.jpg]

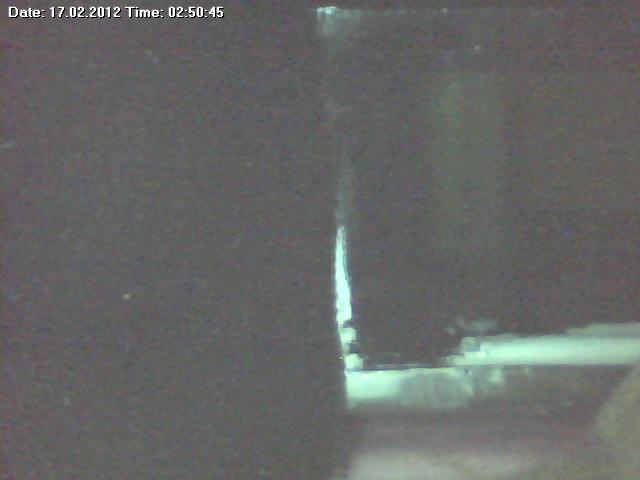

Supplement: Supplementary file 1 — Supplementary material [file mmc1.zip › Supplementary files/Supplementary Figure 1071.jpg]

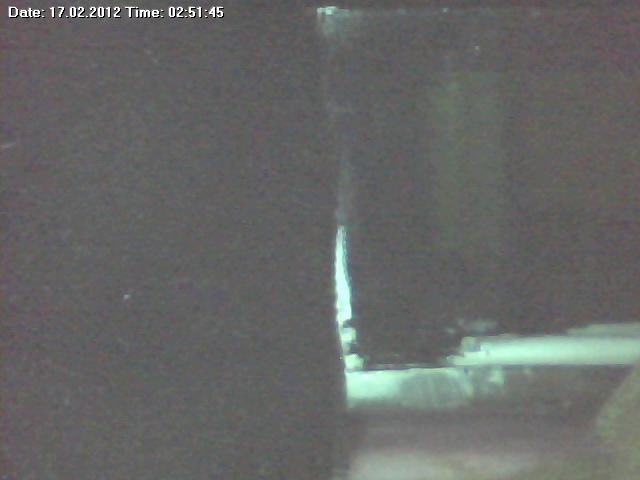

Supplement: Supplementary file 1 — Supplementary material [file mmc1.zip › Supplementary files/Supplementary Figure 1072.jpg]

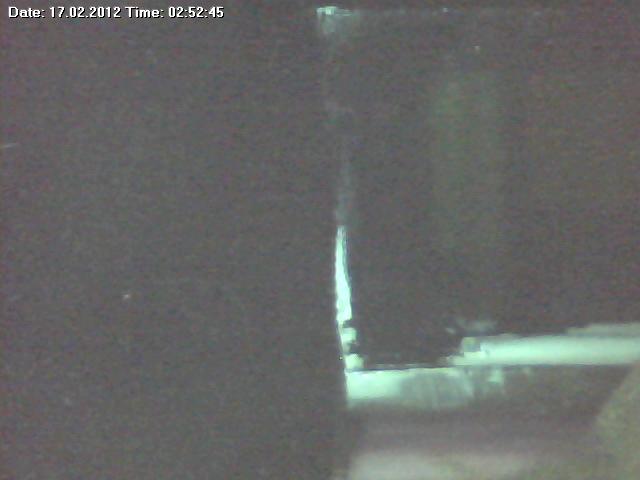

Supplement: Supplementary file 1 — Supplementary material [file mmc1.zip › Supplementary files/Supplementary Figure 1073.jpg]

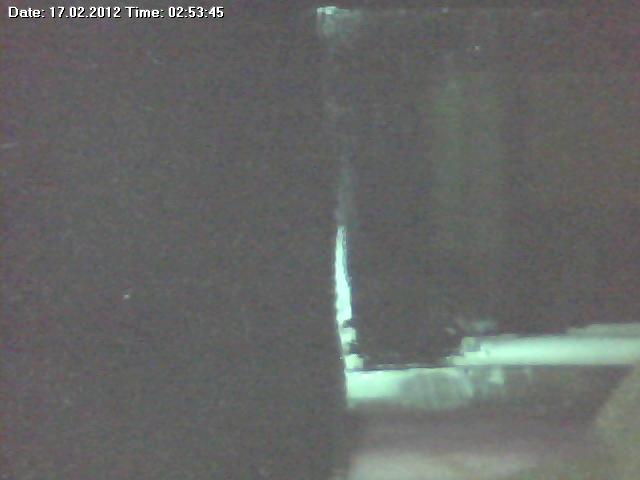

Supplement: Supplementary file 1 — Supplementary material [file mmc1.zip › Supplementary files/Supplementary Figure 1074.jpg]

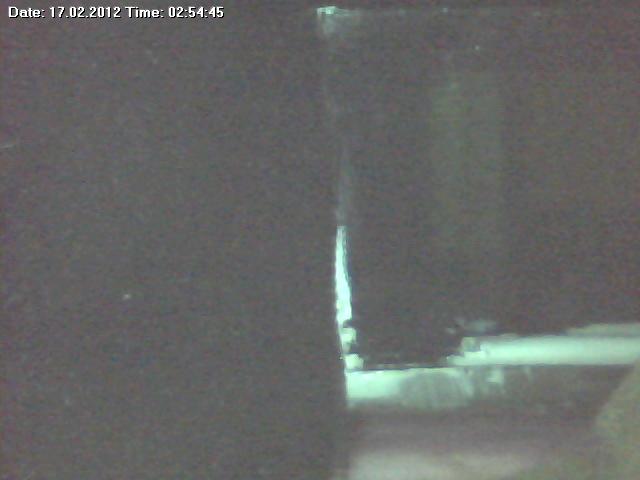

Supplement: Supplementary file 1 — Supplementary material [file mmc1.zip › Supplementary files/Supplementary Figure 1075.jpg]

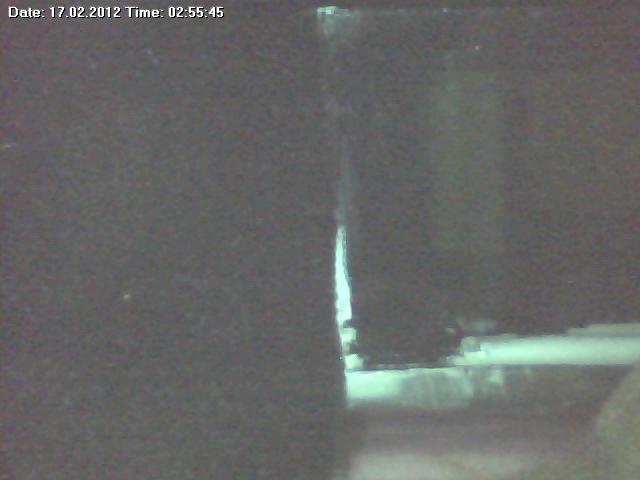

Supplement: Supplementary file 1 — Supplementary material [file mmc1.zip › Supplementary files/Supplementary Figure 1076.jpg]

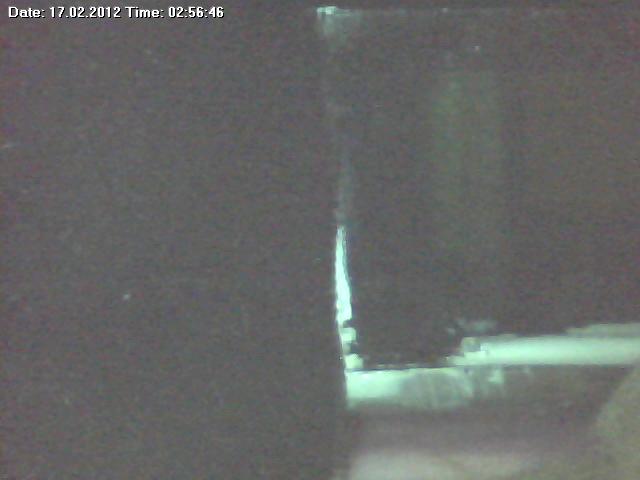

Supplement: Supplementary file 1 — Supplementary material [file mmc1.zip › Supplementary files/Supplementary Figure 1077.jpg]

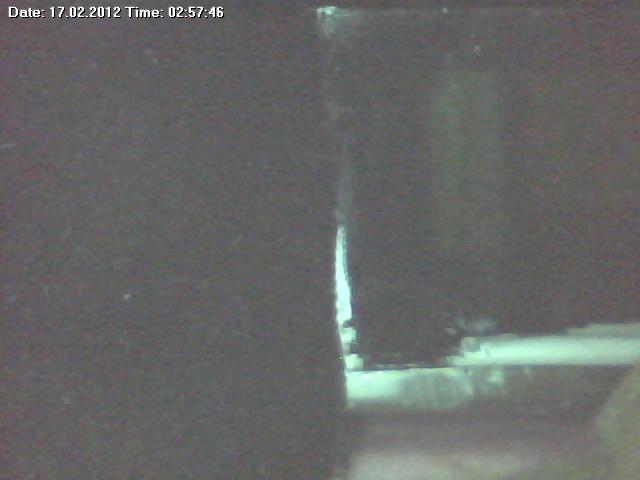

Supplement: Supplementary file 1 — Supplementary material [file mmc1.zip › Supplementary files/Supplementary Figure 1078.jpg]

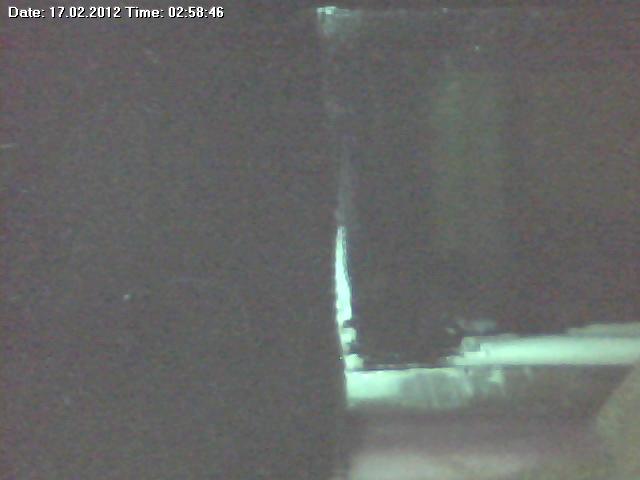

Supplement: Supplementary file 1 — Supplementary material [file mmc1.zip › Supplementary files/Supplementary Figure 1079.jpg]

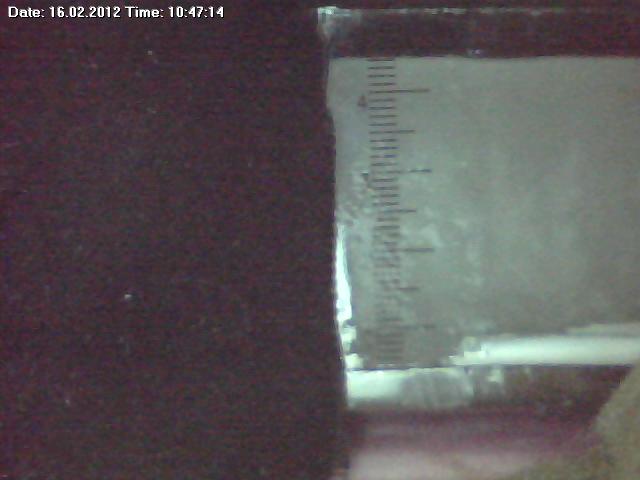

Supplement: Supplementary file 1 — Supplementary material [file mmc1.zip › Supplementary files/Supplementary Figure 108.jpg]

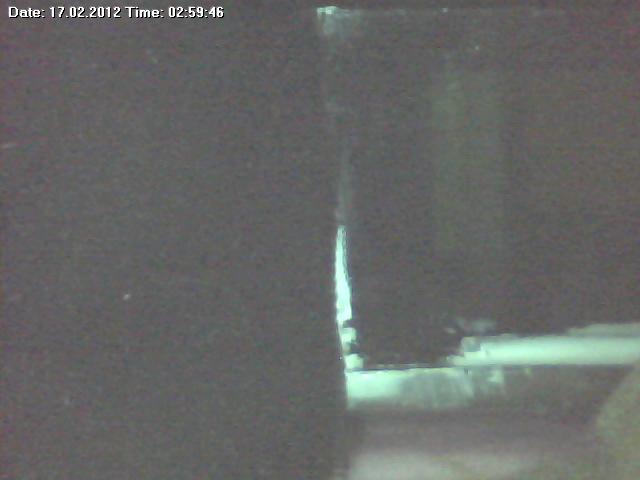

Supplement: Supplementary file 1 — Supplementary material [file mmc1.zip › Supplementary files/Supplementary Figure 1080.jpg]

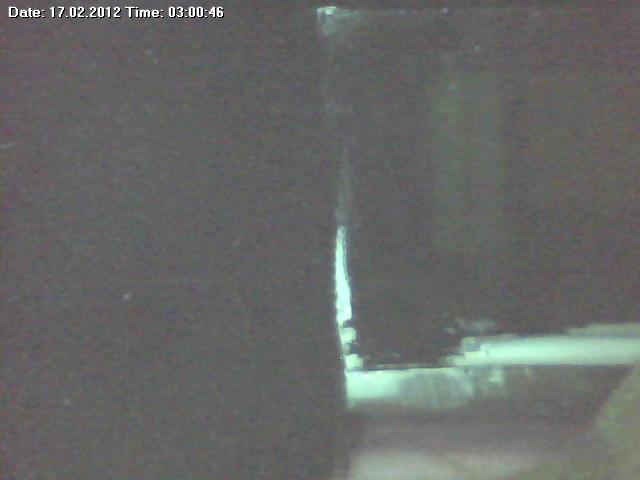

Supplement: Supplementary file 1 — Supplementary material [file mmc1.zip › Supplementary files/Supplementary Figure 1081.jpg]

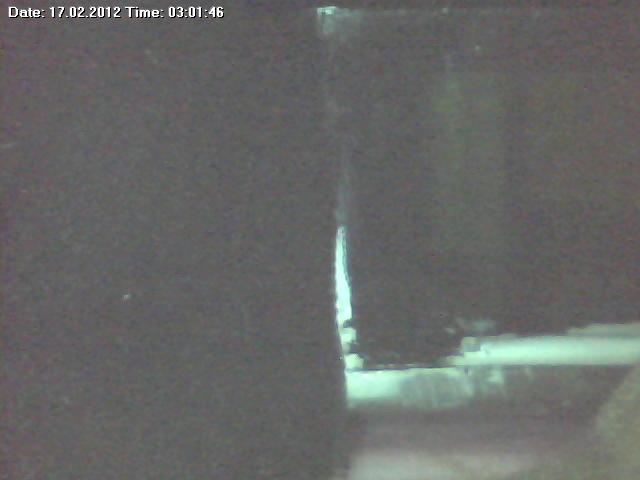

Supplement: Supplementary file 1 — Supplementary material [file mmc1.zip › Supplementary files/Supplementary Figure 1082.jpg]

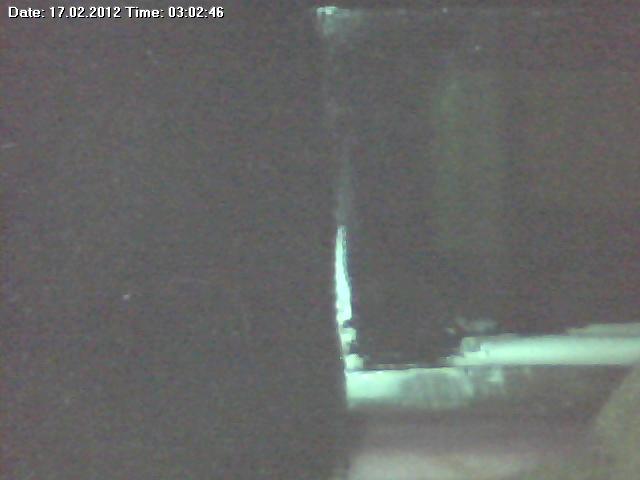

Supplement: Supplementary file 1 — Supplementary material [file mmc1.zip › Supplementary files/Supplementary Figure 1083.jpg]

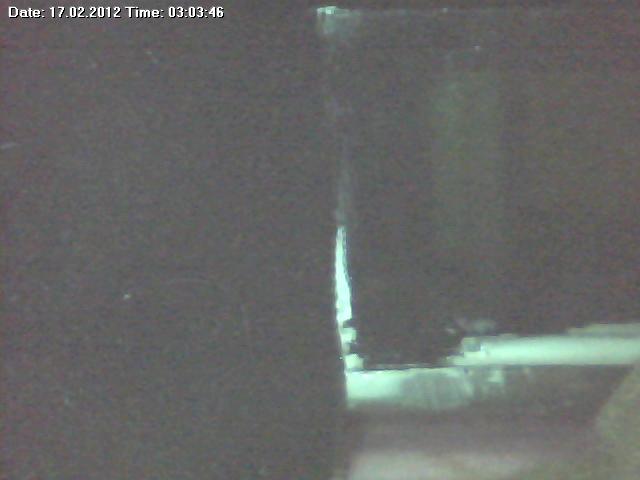

Supplement: Supplementary file 1 — Supplementary material [file mmc1.zip › Supplementary files/Supplementary Figure 1084.jpg]

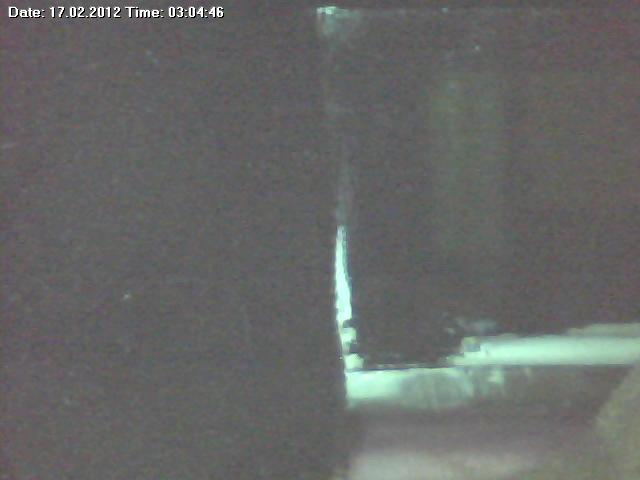

Supplement: Supplementary file 1 — Supplementary material [file mmc1.zip › Supplementary files/Supplementary Figure 1085.jpg]

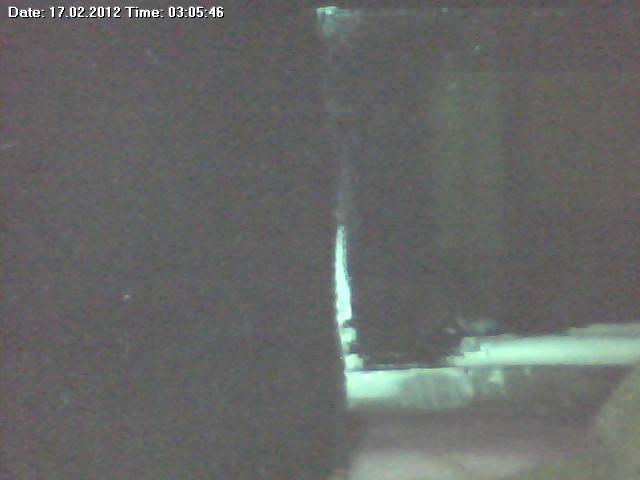

Supplement: Supplementary file 1 — Supplementary material [file mmc1.zip › Supplementary files/Supplementary Figure 1086.jpg]

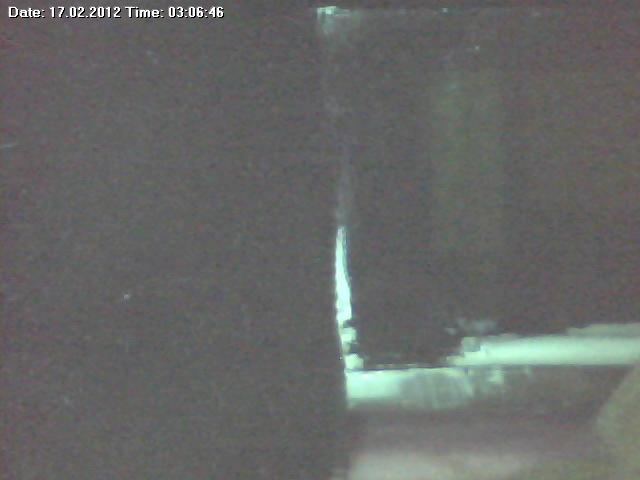

Supplement: Supplementary file 1 — Supplementary material [file mmc1.zip › Supplementary files/Supplementary Figure 1087.jpg]

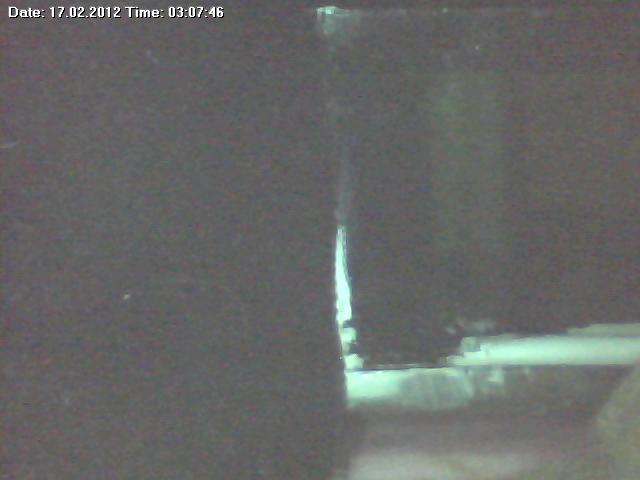

Supplement: Supplementary file 1 — Supplementary material [file mmc1.zip › Supplementary files/Supplementary Figure 1088.jpg]
